# Key lifestyles and health outcomes across 16 prevalent chronic diseases: A network analysis of an international observational study

**DOI:** 10.7189/jogh-14-04068

**Published:** 2024-04-12

**Authors:** Jiaying Li, Daniel Yee Tak Fong, Kris Yuet Wan Lok, Janet Yuen Ha Wong, Mandy Man Ho, Edmond Pui Hang Choi, Vinciya Pandian, Patricia M Davidson, Wenjie Duan, Marie Tarrant, Jung Jae Lee, Chia-Chin Lin, Oluwadamilare Akingbade, Khalid M Alabdulwahhab, Mohammad Shakil Ahmad, Mohamed Alboraie, Meshari A Alzahrani, Anil S Bilimale, Sawitree Boonpatcharanon, Samuel Byiringiro, Muhammad Kamil Che Hasan, Luisa Clausi Schettini, Walter Corzo, Josephine M De Leon, Anjanette S De Leon, Hiba Deek, Fabio Efficace, Mayssah A El Nayal, Fathiya El-Raey, Eduardo Ensaldo-Carrasco, Pilar Escotorin, Oluwadamilola Agnes Fadodun, Israel Opeyemi Fawole, Yong-Shian Shawn Goh, Devi Irawan, Naimah Ebrahim Khan, Binu Koirala, Ashish Krishna, Cannas Kwok, Tung Thanh Le, Daniela Giambruno Leal, Miguel Ángel Lezana-Fernández, Emery Manirambona, Leandro Cruz Mantoani, Fernando Meneses-González, Iman Elmahdi Mohamed, Madeleine Mukeshimana, Chinh Thi Minh Nguyen, Huong Thi Thanh Nguyen, Khanh Thi Nguyen, Son Truong Nguyen, Mohd Said Nurumal, Aimable Nzabonimana, Nagla Abdelrahim Mohamed Ahmed Omer, Oluwabunmi Ogungbe, Angela Chiu Yin Poon, Areli Reséndiz-Rodriguez, Busayasachee Puang-Ngern, Ceryl G Sagun, Riyaz Ahmed Shaik, Nikhil Gauri Shankar, Kathrin Sommer, Edgardo Toro, Hanh Thi Hong Tran, Elvira L Urgel, Emmanuel Uwiringiyimana, Tita Vanichbuncha, Naglaa Youssef

**Affiliations:** 1 School of Nursing, Li Ka Shing Faculty of Medicine, University of Hong Kong, Hong Kong SAR, China; 2 School of Nursing and Health Studies, Hong Kong Metropolitan University, Hong Kong SAR, China; 3 School of Nursing, Johns Hopkins University, Baltimore, Maryland, USA; 4 Vice-Chancellor and Principal, University of Wollongong, Wollongong, Australia; 5 Department of Social Work, East China University of Science and Technology, Shanghai, China; 6 School of Nursing, The University of British Columbia, Kelowna, British Columbia, Canada; 7 The Nethersole School of Nursing, The Chinese University of Hong Kong, Hong Kong SAR, China; 8 Institute of Nursing Research, Osogbo, Osun State, Nigeria; 9 College of Medicine, Majmaah University, Al Majmaah, Saudi Arabia; 10 Department of Family & Community Medicine, College of Medicine, Majmaah University, Al Majmaah, Saudi Arabia; 11 Department of Internal Medicine, Al-Azhar University, Cairo, Egypt; 12 Department of Urology, College of Medicine, Majmaah University, Al Majmaah, Saudi Arabia; 13 School of Public Health, JSS Medical College, JSS AHER, Mysuru, India; 14 Department of Statistics, Chulalongkorn Business School, Bangkok, Thailand; 15 Kulliyyah of Nursing, International Islamic University, Kuantan, Malaysia; 16 Italian Association against Leukemia, Lymphoma and Myeloma (AIL), Rome, Italy; 17 Diálogos Guatemala, Guatemala, Guatemala; 18 School of Nursing, Centro Escolar University, Manila, Philippines; 19 Nursing Department, Faculty of Health Science, Beirut Arab University, Beirut, Lebanon; 20 Italian Group for Adult Hematologic Diseases (GIMEMA), Data Center and Health Outcomes Research Unit, Rome, Italy; 21 Department of Psychology, Beirut Arab University, Beirut, Lebanon; 22 Department of hepatogastroenterology and infectious diseases, Damietta faculty of medicine, Al-Azhar University, Cairo, Egypt; 23 Ergonomics Research Center (ECR), University of Guadalajara, Jalisco, Mexico; 24 Laboratory of Applied Prosocial Research, Department of Basic, Developmental and Educational Psychology, Autonomous University of Barcelona, Barcelona, Spain; 25 Faculty of Health Sciences, University of Lethbridge, Lethbridge, Alberta, Canada; 26 Faculty of Nursing, Ladoke Akintola University of Technology, Ogbomosho, Nigeria; 27 Alice Lee Centre for Nursing Studies, National University of Singapore, Singapore; 28 School of Nursing, Wijaya Husada Health Institute, Bogor, Indonesia; 29 Department of Optometry, University of Kwazulu-Natal, Durban, South Africa; 30 Ecove, Ghaziabad, India; 31 School of Nursing, Paramedicine and Health Care Science, Charles Sturt University, New South Wales, Australia; 32 Nam Dinh University of Nursing, Nam Dinh, Vietnam; 33 Pontificia Universidad Católica de Valparaíso, School of Social Work, Valparaíso, Chile; 34 Research Department, National Commission for Medical Arbitration, Mexico, Mexico; 35 College of Medicine and Health Sciences, University of Rwanda, Kigali, Rwanda; 36 Department of Physiotherapy, Faculty of Science and Technology, São Paulo State University (UNESP), Presidente Prudente, Brazil; 37 Pharmacology and Toxicology Department, Faculty of Pharmacy, Benghazi University, Benghazi, Libya; 38 School of Nursing and Midwifery, College of Medicine and Health Sciences, University of Rwanda, Kigali, Rwanda; 39 Center for Language Enhancement, College of Arts and Social Sciences, University of Rwanda, Huye, Rwanda; 40 Faculty of Medicine, Alzaiem Alazhari University, Khartoum North, Sudan; 41 Faculty of Health Sciences and Sports, Macao Polytechnic University, Macao; 42 National Autonomous University of Mexico, Mexico; 43 Mental Health and Learning division, Wrexham Maelor Hospital, Wrexham, Wales, UK; 44 Medical-surgical Nursing Department, Faculty of Nursing, Cairo University, Cairo, Egypt

## Abstract

**Background:**

Central and bridge nodes can drive significant overall improvements within their respective networks. We aimed to identify them in 16 prevalent chronic diseases during the coronavirus disease 2019 (COVID-19) pandemic to guide effective intervention strategies and appropriate resource allocation for most significant holistic lifestyle and health improvements.

**Methods:**

We surveyed 16 512 adults from July 2020 to August 2021 in 30 territories. Participants self-reported their medical histories and the perceived impact of COVID-19 on 18 lifestyle factors and 13 health outcomes. For each disease subgroup, we generated lifestyle, health outcome, and bridge networks. Variables with the highest centrality indices in each were identified central or bridge. We validated these networks using nonparametric and case-dropping subset bootstrapping and confirmed central and bridge variables' significantly higher indices through a centrality difference test.

**Findings:**

Among the 48 networks, 44 were validated (all correlation-stability coefficients >0.25). Six central lifestyle factors were identified: less consumption of snacks (for the chronic disease: anxiety), less sugary drinks (cancer, gastric ulcer, hypertension, insomnia, and pre-diabetes), less smoking tobacco (chronic obstructive pulmonary disease), frequency of exercise (depression and fatty liver disease), duration of exercise (irritable bowel syndrome), and overall amount of exercise (autoimmune disease, diabetes, eczema, heart attack, and high cholesterol). Two central health outcomes emerged: less emotional distress (chronic obstructive pulmonary disease, eczema, fatty liver disease, gastric ulcer, heart attack, high cholesterol, hypertension, insomnia, and pre-diabetes) and quality of life (anxiety, autoimmune disease, cancer, depression, diabetes, and irritable bowel syndrome). Four bridge lifestyles were identified: consumption of fruits and vegetables (diabetes, high cholesterol, hypertension, and insomnia), less duration of sitting (eczema, fatty liver disease, and heart attack), frequency of exercise (autoimmune disease, depression, and heart attack), and overall amount of exercise (anxiety, gastric ulcer, and insomnia). The centrality difference test showed the central and bridge variables had significantly higher centrality indices than others in their networks (*P* < 0.05).

**Conclusion:**

To effectively manage chronic diseases during the COVID-19 pandemic, enhanced interventions and optimised resource allocation toward central lifestyle factors, health outcomes, and bridge lifestyles are paramount. The key variables shared across chronic diseases emphasise the importance of coordinated intervention strategies.

Noncommunicable diseases (NCDs), also known as chronic diseases, are a major global concern, causing nearly 74% of all deaths and accounting for 41 million fatalities annually [1]. Their impact is most pronounced in low- and middle-income countries, where 86% of premature deaths and 77% of NCD-related fatalities occur [1]. The escalating issue of NCDs, exacerbated by global aging, extends its repercussions from individuals (diminished health status, functional limitations, and elevated mortality rates) to health care systems and economies [2].

Unhealthy lifestyle choices play a pivotal role in the development and progression of NCDs. Their influence extends from underlying mechanisms, such as inflammation, oxidative stress, metabolic dysfunction, insulin resistance, and hormonal imbalances, to the effectiveness of treatments and symptom management [3–5]. For example, individuals with cardiovascular diseases, such as hypertension and high cholesterol, are at a heightened risk of heart attacks and strokes when leading a sedentary lifestyle. This is because physical inactivity creates conditions favourable for atherosclerosis [4]. Likewise, consuming sugary drinks can exacerbate complications in type 2 diabetes [5]. This highlights that all chronic diseases can be impacted by unhealthy lifestyles, but specific diseases may exhibit particular vulnerabilities to certain aspects of lifestyle.

Addressing the profound challenges of NCDs, especially in resource-limited developing countries, necessitates strategic prioritisation of pivotal lifestyle factors and health outcomes. Modifying these critical areas could holistically optimise health metrics, achieving notable cost-efficacy. Indeed, the rationale behind such strategic prioritisation is rooted in the theory of network analysis. Theoretically and statistically, central nodes in a network significantly influence the entire system, while bridge nodes act as connectors and have a significant impact on adjacent networks [6]. Identifying and modifying the central lifestyle factors, central health outcomes, and bridge lifestyles that connect strongly with health outcomes can potentiate sweeping positive shifts, amplifying efficiency and overall outcomes. Empirical evidence attests to the intricate interplay between lifestyle factors and health outcomes: for instance, diet alterations not only influence health directly but cascade effects onto related lifestyle domains like physical activity, thereby modulating health [7]. Such interlinkages align seamlessly with the tenets of network analysis. A previous study has identified central lifestyle and health outcomes, as well as bridge lifestyles, within the general population [8]. However, the unique vulnerabilities inherent to different chronic diseases caution against a blanket approach. Discerning these propensities is essential for nuanced disease understanding and orchestrating synergistic interventions across varied chronic diseases.

This study, anchored in network theory, assesses the interplay between lifestyle factors and health outcomes in chronic diseases. We hypothesise that each chronic disease has distinct central lifestyle factors, health outcomes, and bridge lifestyles. Therefore, we aim to identify these elements across 16 prevalent chronic diseases, providing a foundation for enhanced interventions and efficient resource allocation. This approach aims to facilitate cost-effective, comprehensive improvements in health and lifestyle, particularly in resource-constrained settings.

## METHODS

### Study design and settings

This was an international cross-sectional study. It was conducted across six regions defined by the World Health Organization (WHO) and included a diverse sample from 30 territories. Participants were primarily recruited through online platforms to ensure a wide reach and enable voluntary participation in their preferred language. Detailed information is available in the published protocol for a comprehensive understanding of the study design [9].

### Participants and sample size

We recruited participants aged 18 or older using convenience and snowball sampling methods. The required sample size for each network – lifestyle (18 nodes), health outcome (13 nodes), and bridge (31 nodes) – was calculated based on the maximum number of edges, which were 153, 78, and 465, respectively. As a result, the respective networks required sample sizes of 459, 234, and 1134 participants, in accordance with the guideline of at least three participants per parameter [10,11]. Furthermore, a topological overlap check was conducted, resulting in the removal of certain nodes prior to network estimation. This adjustment often resulted in the actually required sample sizes lower than initially estimated. The study's sample sizes varied from 217 to 1509 across different chronic diseases, with most meeting these revised requirements. Importantly, in network analysis, centrality measures can also be considered reliable even if the sample size does not meet the initial requirements, provided they pass the stability test by case-dropping subset bootstrap [10]. To ensure reliability, we conducted stability test for all the 48 networks and reported only those network results that passed this stability test.

### Measures

#### Socio-demographics

The sociodemographic variables considered in this study encompassed gender, age, country, marital status, highest education attained, employment, perceived social rank, and whether the participant was a practicing health professional.

#### Measurement of chronic diseases

Participants were asked to self-report their medical history by indicating the presence of chronic illnesses, including hypertension, high cholesterol, pre-diabetes/hyperglycaemia, diabetes, fatty liver disease, heart attack, stroke, chronic obstructive pulmonary disease (COPD), cancer, gastric ulcer, irritable bowel syndrome, insomnia, depression, anxiety, eczema, nephropathy, autoimmune disease, Parkinson disease, hearing problems, and epilepsy. Participants were also provided an option to indicate if they did not have any chronic diseases.

#### Measurement of lifestyle factors and health outcomes

The development of our questionnaire, designed to evaluate 18 lifestyle factors and 13 health outcomes, involved a comprehensive literature review and collaborative discussions with a team of public health professionals, nurses, and nutritionists in Hong Kong. This process produced an initial English version focused on meeting the study's objectives and ensuring face validity. Rigorous reviews refined the questionnaire for clarity, eliminated redundancies, and structured the flow of questions for ease of completion. Cultural relevance was ensured through consultations with experts in various countries, followed by translation into multiple languages using a standard forward-backward process. Single items were used to assess the lifestyle factors and health outcomes, and thus formal psychometric evaluation was not applicable. Nevertheless, we pilot tested the questionnaire with a minimum of 10 participants per country to ensure the consistency in data interpretation and comprehension across different cultural contexts [9].

Participants were asked to rate the impact of COVID-19 on 18 lifestyle factors and 13 general health outcomes using a 5-point Likert scale (1 = substantially reduced, 3 = no change, 5 = substantially increased). To maintain a consistent alignment of item directions reflecting a healthier lifestyle and improved health outcomes, certain items were re-coded in reverse by adding a ‘less’ prefix.

### Data collection

We collected data anonymously using online survey platforms and offline electronic forms (in PDF format), accommodating regions with restricted internet access. Participation was encouraged by donating one Hong Kong dollar to the Red Cross for each completed questionnaire. The response rate was calculated at 75.2%.

### Validation and rigor

To improve internal validity, a validation question was included, asking participants: ‘Where does the sun rise every day?’ In Nigeria, another validation question ‘Where is your STATE capital?’ was used for better cultural relevance. Prior to administering the questionnaires in a country, a pilot study was conducted involving at least ten participants.

### Statistical analysis

Data collected for our study were transferred to a Microsoft Excel database for stringent quality control, involving removing incomplete or duplicate responses to maintain data integrity. We also checked data inconsistencies, discarding responses misaligned with validation questions, such as incorrect answers about sunset times or capital cities. Responses where participants selected 'none' for chronic diseases yet reported specific conditions, or those indicating all listed diseases, were identified as inconsistent and excluded. This thorough data cleaning was crucial to enhance data set accuracy and ensure the validity of our analysis. Subsequent analysis was conducted using R Statistical Software (v4.1.1; R Core Team 2021). The network analyses covered four domains, including topological overlap assessment, network estimation, network stability, and computation of centrality and bridge centrality indices.

#### Checking topological overlap

To avoid artificial relationships caused by similar variables within a network, we used the goldbricker function from the networktools R package to compare correlations and identify unique variables. We used a significance proportion of 0.25 for inclusion and a 0.05 level of significance to determine statistical significance [11].

#### Network estimation

We obtained three networks for each chronic disease group: one comprising all remained lifestyles, one comprising all remained health outcomes, and a bridge network connecting the two. Partial correlation analysis was employed to estimate pairwise associations while controlling for the confounding effects of all other nodes. The least absolute shrinkage and selection operator (LASSO) method was applied to shrink edges and set small correlations to zero, while the extended Bayesian Information Criteria (EBIC) was used to select a related turning parameter and create a more interpretable and sparser network [10]. R packages of bootnet and qgraph were utilised to estimate and visualise the network respectively [10]. In network visualisation, nodes represented network items, while edges depicted their relationships. The thickness of the edges reflected the strength of association, with blue indicating positive associations and red indicating negative associations.

#### Network stability

We assessed the stability of edges and centrality using the bootnet package [10]. Edge weight stability was assessed using 95% confidence intervals (CIs), generated by nonparametric bootstrapping. Narrower CIs signified a network of higher credibility [10]. On the other hand, centrality stability was measured by the Correlation Stability Coefficient (CS-C), obtained by case-dropping subset bootstrap. Acceptable stability was indicated by a CS-C value above 0.25, and preferably surpassing 0.5 [10].

#### Central node, centrality, bridge node, and bridge centrality

A central node is a crucial node within a network that holds significant influence or plays an important role due to its connections with other nodes [12]. Bridge nodes in network analysis serve to connect different clusters of nodes in a network that would otherwise be disconnected [13]. To identify these nodes, centrality measures – (bridge) strength, betweenness, closeness, and expected influence were usually adopted. Bridge strength refers to the degree a bridge/connection between two clusters of nodes is supported by alternative paths. Betweenness centrality quantifies how often a node lies on the shortest path between two other nodes. Closeness centrality of node refers to the shortest distance of the node to all other nodes in the network. Expected influence predicts how much direct and indirect influence a node would have on others. Owing to the instability of betweenness and closeness measures [6], and the presence of negative edges in our networks, we adopted expected influence for determining central nodes and bridge expected influence for bridge nodes.

The 'qgraph' package in R was used to calculate the expected influence index, which considers both positive and negative edges, identifying central nodes with the highest values. Bridge nodes were determined using the 'networktools' package in R, based on the bridge expected influence (one-step) index that aggregates a node's edges to all nodes in other networks [12]. Differences in centrality between two nodes were assessed using Wilcoxon tests, drawing from 1000 bootstrapped indices via bootnet package in R. Holm-Bonferroni corrections addressed multiple comparisons.

## RESULTS

### Demographics and item description

**Figure 1:**
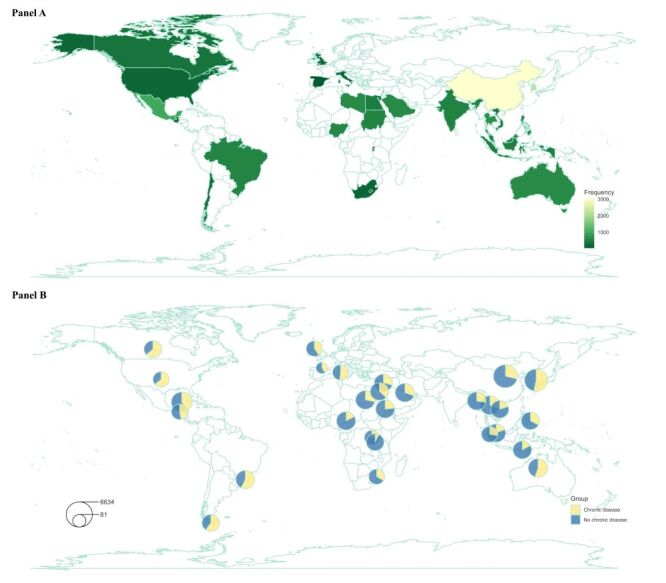
Geographical distribution of the (**Panel A**) overall sample, with (**Panel B**) breakdown by chronic disease (n = 16 512).

Out of the initial 19 145 responses, 16 512 were eligible for analysis after excluding blank or incomplete responses (n = 1940), duplicates (n = 116), inconsistent responses (n = 450), responses from outside the 30 participating countries (n = 126), and those lacking age or gender information (n = 1). The geographical distribution of all participants and a breakdown by chronic disease in each territory are shown in **Figure 1**. Among the validated responses, 5928 individuals (35.9%) reported having at least one chronic disease. The sample in this study generally shows great representativeness, with comparisons of age and gender distribution with those of the respective populations (Figure S1 in the **Online Supplementary Document****)**. Sociodemographic information and details on lifestyle and health outcomes, including abbreviations used in the network structure, means, and standard deviations (SDs), are presented in **Table 1**. This is complemented by a description of means and SDs across different chronic disease subgroups (Table S1 in the **Online Supplementary Document**).

**Table 1:** Demographics and measurement descriptives for respondents with chronic diseases (n = 5928)

Variables	no (%)
**Demographics**	
Age in years	
*18–24*	1067 (18.0)
*25–29*	595 (10.0)
*30–34*	535 (9.0)
*35–39*	613 (10.3)
*40–44*	554 (9.3)
*45–49*	535 (9.0)
*50–54*	592 (10.0)
*55–59*	435 (7.3)
*60–64*	532 (9.0)
*> = 65*	470 (7.9)
Gender	
*Male*	2333 (39.4)
*Female*	3551 (59.9)
*Non-binary*	44 (0.7)
Country	
*Australia*	352 (5.9)
*Brazil*	317 (5.3)
*Burundi*	27 (0.5)
*Canada*	235 (4.0)
*Chile*	204 (3.4)
*Egypt*	172 (2.9)
*Guatemala*	108 (1.8)
*Hong Kong*	689 (11.6)
*India*	145 (2.4)
*Indonesia*	79 (1.3)
*Italy*	105 (1.8)
*Lebanon*	129 (2.2)
*Libya*	183 (3.1)
*Macau*	65 (1.1)
*Mainland China*	127 (2.1)
*Malaysia*	100 (1.7)
*Mexico*	419 (7.1)
*Nigeria*	102 (1.7)
*Philippines*	151 (2.5)
*Republic Of Sudan*	127 (2.1)
*Rwanda*	32 (0.5)
*Saudi Arabia*	194 (3.3)
*Singapore*	66 (1.1)
*South Africa*	66 (1.1)
*South Korea*	1187 (20.0)
*Spain*	21 (0.4)
*Thailand*	221 (3.7)
*UK*	86 (1.5)
*USA*	136 (2.3)
*Vietnam*	83 (1.4)
Marital status	
*Married/cohabitation/common-law*	3207 (54.1)
*Separated/divorced/widowed*	442 (7.5)
*Single*	2279 (38.4)
Education	
*Primary or below*	201 (3.4)
*Secondary*	863 (14.6)
*Associate degree*	561 (9.5)
*College*	879 (14.8)
*Bachelor*	2081 (35.1)
*Graduate*	1236 (20.9)
*Missing data*	107 (1.8)
Employment	
*Job seeking*	314 (5.3)
*Laid off*	72 (1.2)
*Not in workforce*	483 (8.1)
*Retired*	462 (7.8)
*Self-employed*	538 (9.1)
*Student*	1019 (17.2)
*Working (> = 40 h/week)*	2010 (33.9)
*Working (1–39 h/week)*	1030 (17.4)
Lifestyles and health outcomes*	**Mean (SD)**
Food types in daily meals (L1)	3.00 (0.89)
Consumption of fruits and vegetables (L2)	3.13 (0.91)
Less consumption of frozen food/food products (L3)	3.07 (0.97)
Less consumption of snacks (L4)	2.99 (1.02)
Less soft drinks/juices/other sugary drinks (L5)	2.82 (1.05)
Having a meal at home (L6)	3.95 (0.97)
Cooking at home (L7)	3.90 (0.96)
Less eating takeout food (L8)	3.01 (1.20)
Taking alternative medicine or natural health products (L9)	2.96 (0.88)
Taking oral supplements/vitamins (L10)	3.17 (0.91)
Less smoking tobacco (L11)	2.65 (0.96)
Less alcohol consumption (L12)	2.66 (0.99)
Less duration of sitting (L13)	3.76 (0.96)
Less duration of screen time (L14)	3.83 (0.95)
Frequency of exercise (L15)	2.72 (1.12)
Duration of exercise (L16)	2.70 (1.10)
Type of exercise (L17)	2.68 (1.06)
Overall amount of exercise (L18)	2.68 (1.11)
Lose weight (H1)	3.25 (0.92)
Appetite (H2)	3.12 (0.85)
Physical health (H3)	2.82 (0.84)
Sleep quality (H4)	2.72 (0.98)
Quality of life (H5)	2.56 (0.99)
Less mental burden (H6)	3.50 (1.12)
Less emotional distress (H7)	3.47 (1.09)
Family disputes (H8)	3.14 (0.88)
Social support provided (H9)	3.06 (0.90)
Social support received (H10)	2.93 (0.87)
Social activities (H11)	2.18 (1.05)
Income (H12)	2.60 (0.94)
Less economic burden (H13)	3.27 (1.04)

L – lifestyle, H – health outcome

*Scored on a 5-point Likert scale: 1 = substantially reduced; 3 = no change; 5 = substantially increased.

### Non-redundant items in all networks across chronic disease subgroups

The number of non-redundant items ranged between 13 and 17 in the lifestyle networks, and between 10 and 13 in the health outcome networks. Redundant pairs were identified during the Goldbricker analysis, and one item from each pair was removed based on the explained rationale (Table S2 in the **Online Supplementary Document****)**.

### Lifestyle networks across chronic disease subgroups

#### Network stability

To ensure reliable estimates, the accuracy of edge weight in the lifestyle networks of chronic disease subgroups was verified through a bootstrapped 95% CI analysis (Figure S2 in the **Online Supplementary Document**). The CS-C values for expected influence, computed using a case-dropping subset bootstrap procedure, ranged from 0.52 to 0.75. These values exceeded the 0.25 threshold, indicating the interpretability of all lifestyle networks across 16 chronic diseases (**Table 2**, Figure S3 in the **Online Supplementary Document**).

**Table 2:** Summary of network measures across chronic disease subgroups (n = 5928)

Chronic diseases, n	Lifestyle network						Health outcome network							Bridge network			
	**Nonzero/total edges, n (%)**	Largest edge (PC-C*)	**Second largest edge (PC-C)**	**Third largest edge (PC-C)**	**CS-C† value**	**Central lifestyle**	**Nonzero/total edges, n (%)**	**Largest edge (PC-C)**	**Second largest edge (PCC)**	**Third largest edge (PC-C)**	**CS-C value**	**Central health outcome**	**Nonzero/total edges (%)**		**CS-C value**	**Bridge lifestyle**	**Bridge edge (PC–C)**
Anxiety, 1159	79/120 (65.8)	L18-L17 (0.81)	L7-L6 (0.65)	L14-L13 (0.61)	0.75	L4	46/78 (59.0%)	H7-H6 (0.66)	H2-H1 (0.47)	H5-H4 (0.41)	0.75	H5	141/406 (34.7%)		0.59	L18	L18-H3 (0.11)
Autoimmune disease, 532	53/120 (44.2)	L14-L13 (0.66)	L7-L6 (0.57)	L18-L16 (0.50)	0.75	L18	24/66 (36.4%)	H10-H9 (0.38)	H2-H1 (0.37)	H8-H7 (0.34)	0.75	H5	116/378 (30.7%)		0.28	L15	L15-H5 (0.04)
Cancer, 292	16/105 (15.2)	L14-L13 (0.43)	L12-L11 (0.34)	L7-L6 (0.26)	0.52	L5	21/66 (31.8%)	H2-H1 (0.35)	H10-H9 (0.30)	H5-H3 (0.26)	0.44	H5	42/351 (12.0%)		0.13	NA‡	NA
Chronic obstructive pulmonary disease, 340	33/91 (36.3)	L18-L15 (0.76)	L7-L6 (0.69)	L12-L11 (0.55)	0.75	L11	13/45 (28.9%)	H7-H6 (0.51)	H2-H1 (0.28)	H8-H7 (0.25)	0.75	H7	39/276 (14.1%)		0.13	NA	NA
Depression, 981	53/105 (50.5)	L18-L15 (0.78)	L7-L6 (0.62)	L14-L13 (0.60)	0.75	L15	34/78 (43.6%)	H7-H6 (0.70)	H2-H1 (0.45)	H10-H9 (0.34)	0.75	H5	136/378 (36.0%)		0.59	L15	L15-H3 (0.14)
Diabetes, 617	59/136 (43.4)	L7-L6 (0.63)	L12-L11 (0.59)	L14-L13 (0.56)	0.75	L18	35/66 (53.0%)	H2-H1 (0.50)	H8-H7 (0.35)	H5-H4 (0.34)	0.75	H5	151/406 (37.2%)		0.36	L2	L2-H9 (0.15)
Eczema, 681	65/153 (42.5)	L7-L6 (0.67)	L14-L13 (0.59)	L12-L11 (0.49)	0.75	L18	27/78 (34.6%)	H7-H6 (0.67)	H2-H1 (0.39)	H10-H9 (0.30)	0.75	H7	138/465 (29.7%)		0.36	L13	L13-H11 (0.10)
Fatty liver disease, 542	54/91 (59.3)	L16-L15 (0.60)	L12-L11 (0.53)	L5-L4 (0.42)	0.75	L15	35/78 (44.9%)	H7-H6 (0.69)	H2-H1 (0.41)	H10-H9 (0.36)	0.75	H7	116/351 (33.1%)		0.36	L13	L13-H1 (0.10); L13-H11 (0.10)
Gastric ulcer, 579	48/91 (52.8)	L7-L6 (0.68)	L12-L11 (0.60)	L5-L4 (0.35)	0.67	L5	44/78 (56.4%)	H7-H6 (0.68)	H2-H1 (0.40)	H10-H9 (0.39)	0.75	H7	103/351 (29.4%)		0.44	L18	L18-H3 (0.14)
Hearing problems, 217	25/105 (23.8)	L14-L13 (0.46)	L18-L16 (0.44)	L12-L11 (0.41)	0.67	L18	21/66 (31.8%)	H2-H1 (0.35)	H5-H4 (0.25)	H8-H7 (0.24)	0.21	NA	44/351 (12.5%)		0.05	NA	NA
Heart attack, 533	53/120 (44.2)	L14-L13 (0.66)	L7-L6 (0.57)	L18-L16 (0.50)	0.75	L18	28/78 (35.9%)	H7-H6 (0.65)	H10-H9 (0.38)	H2-H1 (0.37)	0.75	H7	109/406 (26.9%)		0.28	L15 = L13	L15-H5 (0.05); L13-H8 (0.08)
High cholesterol, 1283	73/120 (60.8)	L14-L13 (0.62)	L18-L15 (0.58)	L12-L11 (0.52)	0.75	L18	41/78 (52.6%)	H7-H6 (0.65)	H5-H4 (0.37)	H2-H1 (0.36)	0.75	H7	172/406 (42.4%)		0.44	L2	L2-H9 (0.11)
Hypertension, 1509	84/120 (70.0)	L18-L15 (0.83)	L7-L6 (0.67)	L14-L13 (0.62)	0.75	L5	41/78 (52.6%)	H7-H6 (0.63)	H2-H1 (0.46)	H10-H9 (0.40)	0.75	H7	175/406 (43.1%)		0.52	L2	L2-H3 (0.10)
Insomnia, 722	68/120 (56.7)	L18-L17 (0.75)	L7-L6 (0.60)	L14-L13 (0.58)	0.52	L5	41/78 (52.6%)	H7-H6 (0.66)	H2-H1 (0.43)	H5-H4 (0.36)	0.75	H7	122/406 (30.1%)		0.52	L18 = L2	L18-H3 (0.10); L2-H3 (0.15)
Irritable bowel syndrome, 533	53/120 (44.2)	L14-L13 (0.66)	L7-L6 (0.57)	L18-L16 (0.49)	0.75	L16	24/66 (36.4%)	H10-H9 (0.38)	H2-H1 (0.37)	H8-H7 (0.34)	0.75	H5	114/378 (30.2%)		0.21	NA	NA
Pre-diabetes, 378	36/91 (39.6)	L14-L13 (0.59)	L5-L4 (0.47)	L12-L11 (0.43)	0.52	L5	22/78 (28.2%)	H7-H6 (0.54)	H2-H1 (0.37)	H10-H9 (0.32)	0.67	H7	83/351 (23.7%)		0.21	NA	NA

L – lifestyle, H – health outcome, NA – not applicable

*PC-C: partial correlation coefficient

†CS-C: correlation stability coefficient.

#### Network structure and central lifestyle

**Figure 2:**
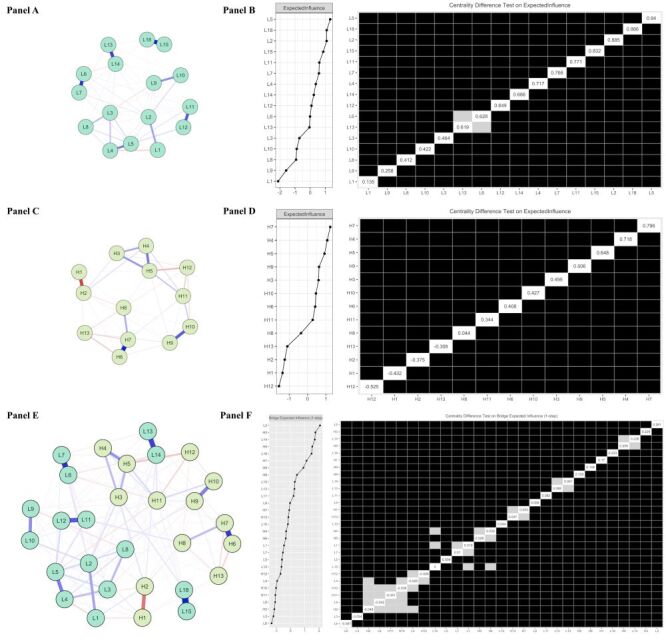
Network structure and centrality difference test of lifestyles (**Panel A** and **Panel B**), health outcomes (**Panel C** and **Panel D**), and combined (**Panel E** and **Panel F**) in patients with hypertension. *The abbreviations of nodes in Panels A, C, and E can be found in **Table 1**. In Panels B, D, and F, a grey cell indicates that there is no significant difference between the corresponding two variables. A dark cell indicates that there is a significant difference between the corresponding two variables at 5% level of significance. A white cell displays the value of the expected influence or bridge expected influence.

Using hypertension as an example, in lifestyle network structure, edges represent partial correlations between nodes (**Figure 2**, panel A). Of the 120 edges, 70% were nonzero, indicating robust connectivity. The top three edges (partial correlation coefficient) were lifestyle 18-Lifestyle 15 (L18-L15) = 0.83), L7-L6 = 0.67, and L14-L13 = 0.62 (**Table 2****)**. Additionally, the most central node was identified as less sugary drinks (L5), confirming its centrality through the centrality index plot and centrality bootstrapped difference test (*P* < 0.05) (**Figure 2**, panel B) Regarding the other 15 chronic diseases, Figure S4 in the **Online Supplementary Document** displays the network structures, centrality index plots, and centrality bootstrapped difference tests for other chronic diseases. **Table 2** summarises the percentage of nonzero edges (ranging from 23.8% to 70.0%), along with the top three largest edges and significant central lifestyles. For all chronic diseases, the partial correlation coefficients for all edges are detailed in Table S3 in the **Online Supplementary Document**.

### Health outcome networks across chronic disease subgroups

#### Network stability

Narrow CIs indicated high precision of estimated edge weights across chronic diseases (Figure S5 in the **Online Supplementary Document**). The CS-C values ranged from 0.21 to 0.75 (**Table 2**, Figure S6 in the **Online Supplementary Document**). Only the hearing problems subgroup fell below the threshold of 0.25, resulting in 15 interpretable health outcome networks.

#### Network structure and central health outcome

In network structure for hypertension, 52.6% (41 out of 78) of edges being nonzero (**Figure 2**, panel C). The top three largest edges (partial correlation coefficient) were health outcome 7-health outcome 6 (H7-H6) = 0.63, H2-H1 = 0.46, and H10-H9 = 0.40 (**Table 2**). Among the health outcomes, less emotional distress (H7) stood out with the highest expected influence, as confirmed by the centrality index plot and centrality bootstrapped difference test (*P* < 0.05) (**Figure 2**, panel D). Regarding the other 14 chronic diseases, Figure S4 in the **Online Supplementary Document** presents network structures, centrality index plots, and centrality bootstrapped difference tests for other chronic diseases. **Table 2** summarises the percentages of nonzero edges (ranging from 28.2 to 59.0%), the top three edges with their partial correlation coefficients, and the central health outcome that significantly differs from others in each network. The partial correlation matrix for other edges across all chronic diseases is provided in Table S4 in the **Online Supplementary Document**.

### Bridge networks across chronic disease subgroups

#### Network stability

The precision of estimated edge weights is demonstrated, showing narrow CIs and high accuracy (Figure S7 in the **Online Supplementary Document**). The CS-C values for bridge networks ranged from 0.13 to 0.75 (**Table 2**, Figure S8 in the **Online Supplementary Document****)**. Among the chronic diseases, cancer, COPD, hearing problems, irritable bowel syndrome, and pre-diabetes fell below the 0.25 threshold, resulting in 11 interpretable bridge networks.

#### Network structure and bridge lifestyle

In the hypertension network structure (**Figure 2**, panel E), 175 out of 406 edges were nonzero (**Table 2**). Among the 18 lifestyles, consumption of fruit and vegetables (L2) demonstrated the highest bridge expected influence. This influence was significantly higher than that of all other lifestyle nodes, as confirmed by the centrality bootstrapped difference test (*P* < 0.05) (**Figure 2**, panel F). Thus, L2 exerted the most significant influence over the 13 health outcome nodes, with the corresponding bridge edge being L2-H3 (physical health). Regarding the other 10 chronic diseases, Figure S4 in the **Online Supplementary Document** presents the network structures, bridge centrality index plots, and centrality bootstrapped difference tests for other chronic diseases. **Table 2** summarises the percentage of nonzero edges (12.0–43.1%) and identifies the bridge lifestyles along with their corresponding bridge edges. The partial correlation matrix for the remaining edges across all 11 diseases can be found in Table S5 in the **Online Supplementary Document**.

**Figure 3:**
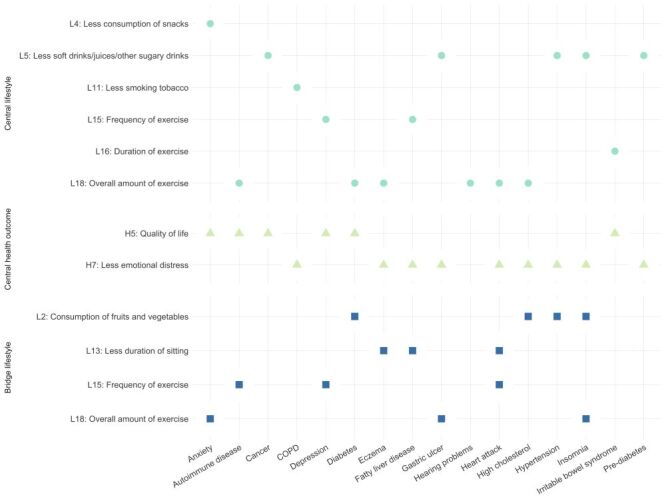
Central lifestyles, central health outcomes, and bridge lifestyles by chronic diseases. COPD – chronic obstructive pulmonary disease

In summary, **Figure 3** depicts the distribution of central lifestyles, central health outcomes, and bridge lifestyles across all chronic diseases.

## DISCUSSION

This study uniquely advances NCD management by identifying central lifestyle factors, central health outcomes, and bridge lifestyle factors as targeted points for interventions and resource allocation. Our meticulous analysis has identified six noteworthy central lifestyles, two distinct central health outcomes, and four remarkable bridge lifestyles, offering profound insights to bolster strategic planning, resource allocation, and intervention design in chronic disease management. Importantly, modifications to the central lifestyle are anticipated to yield comprehensive improvements across all lifestyles, which could, in turn enhance all lifestyle-related health outcomes, including mortality. Central health outcomes suggest alternative avenues for intervention that extend beyond traditional lifestyle modifications, encompassing aspects such as health care service utilisation, to comprehensively improve the health outcomes we examined. Bridge lifestyles delineate specific pathways through which lifestyle adjustments can optimise the overall health outcomes we included. Furthermore, the presence of shared central or bridge variables across various chronic diseases highlights opportunities for collaborative strategies.

While a previous study identified sugary drinks and fruit or vegetable consumption as central lifestyles in the general population [8], our research across 16 chronic disease groups revealed six distinct central lifestyle factors. This variation likely stems from the unique needs of individuals with chronic diseases. For instance, fruit consumption might reduce sugary drink intake generally, but this link may not apply to diabetics who are already advised against sugary drinks. The reduction of sugary drink consumption has emerged as a central lifestyle change for managing conditions such as hypertension, pre-diabetes, cancer, gastric ulcers, and insomnia. It exacerbates metabolic disorders and contributes to weight gain, both crucial factors in hypertension development [14]. Sugary drinks also contribute to obesity, a notable risk factor for cancer, with their high glycaemic index and chemical compounds potentially influencing carcinogenesis [15]. The link between sugar intake and insomnia is associated with increased inflammation and magnesium depletion, which impact sleep [16]. The specific mechanism by which sugary drinks affect gastric ulcers is less documented and warrants further study. Conversely, for conditions like high cholesterol, diabetes, heart attacks, eczema, autoimmune diseases, and hearing issues, exercise is key. It improves cholesterol metabolism by reducing body fat and weight, enhancing hormone and enzyme activity, and increasing 'good' high-density lipoprotein cholesterol [17]. Exercise also benefits cardiovascular health by reducing heart attack risks through antiatherogenic adaptations and myocardial regeneration [18], and aids autoimmune disease management by modulating immune cell functions and reducing inflammation [19]. Additionally, cardiovascular fitness, improved by exercise, is positively correlated with better hearing through enhanced inner ear blood circulation [20].

Interestingly, central lifestyles differ between pre-diabetes and diabetes. Reducing sugary drinks is central for pre-diabetes, possibly because it aids in stabilising blood glucose levels and helps prevent progression to diabetes [21]. Conversely, exercise emerges as the central lifestyle for diabetes. This might be because individuals with diabetes are often under strict dietary regimes, and exercise aids in glucose uptake, lowers blood glucose levels, reduces insulin dependence, and improves overall glycaemic control [22].

Four additional central lifestyle changes were identified: reduced snack consumption for anxiety, less smoking for COPD, more frequent exercise for depression and fatty liver disease, and longer exercise duration for irritable bowel syndrome. Frequent savoury snacking correlates with psychological health issues through cognitive failures, impacting anxiety levels [23]. Quitting smoking, which damages lung airways and impairs function, helps prevent worsening of COPD symptoms and improves overall lung health [24]. Regular exercise influences brain structure and function, especially in areas linked to depression, leading to improvements in regions like the hippocampus and alleviating depressive symptoms [25]. Exercise improves liver metabolism by enhancing insulin resistance and increasing fatty acid oxidation, simultaneously reducing hepatic fat accumulation and fatty acid synthesis [26]. Physical activity, by influencing bowel movements and colon transit, can significantly improve gastrointestinal symptoms for irritable bowel syndrome patients [27].

To optimise holistic lifestyle improvements in chronic diseases, our study underscores the need for enhanced interventions or more resource allocation on their central lifestyle. For anxiety management, community education and health care dietary counselling can reduce snack consumption. Taxing sugary drinks and promoting healthier alternatives are advised for conditions like cancer and hypertension. Smoking cessation in COPD should be supported by public policy and health care. Enhancing exercise frequency, duration, and overall activity is crucial in diseases like depression, irritable bowel syndrome, autoimmune disorders, diabetes, eczema, heart attack, and high cholesterol, utilising community exercise programmes, workplace wellness initiatives, patient education, and urban planning for active lifestyles.

Our study revealed that in chronic diseases, less emotional distress and quality of life varied in centrality, contrasting with their co-dominance in the general population [8]. This variation likely stems from the specific challenges and impacts of each chronic disease on individuals' overall well-being. Notably, hypertension, high cholesterol, pre-diabetes, fatty liver disease, heart attack, COPD, gastric ulcer, insomnia, and eczema shared the common central health outcome of emotional distress. Such diseases often involve physiological changes or symptoms directly leading to emotional distress, stemming from fears of disease progression, recurrence, breathing difficulties, chronic pain, or skin-related concerns [22, 28–33]. Conversely, diabetes, cancer, irritable bowel syndrome, depression, anxiety, and autoimmune disease all shared the central health outcome of quality of life. Patients with these conditions often experience pronounced impairments in physical functioning due to symptoms, treatment side effects, fatigue, or functional limitations, all of which significantly affect their daily activities and overall well-being [34–37].

While overall amount of exercise was identified as the bridge lifestyle in the general population [8], our chronic disease-focused study reveals four unique bridge lifestyles, highlighting the diverse lifestyle-health outcome connections specific to each chronic disease. Consumption of fruits and vegetables, which are nutrient-dense and abundant in vitamins, minerals, fibre, and antioxidants, emerged as a bridge lifestyle for conditions like hypertension, high cholesterol, diabetes, and insomnia. These nutrients offer multiple benefits, notably in cardiovascular health, blood pressure regulation, and glycaemic control [38], thus aiding individuals with metabolic disorders. In addition, reducing sitting hours is identified as the bridge lifestyle for those with fatty liver disease, heart attack, and eczema. The detrimental effects of prolonged sitting, such as impaired glucose metabolism and weakened cardiovascular health, underline this [39–41]. Furthermore, exercise frequency serves as a bridge lifestyle for heart attack, depression, and autoimmune disease, while the overall amount of exercise acts as the bridge lifestyle for gastric ulcer, insomnia, and anxiety. Exercise offers benefits such as improved cardiovascular fitness, endorphin release, neurotransmitter modulation, immune system regulation, inflammation reduction, and enhanced immune function, improving general health outcomes for individuals with these chronic diseases [42]. As a lifestyle-specific approach to improve the 13 health outcomes we studied, these bridge lifestyles also warrant enhanced intervention and resource allocation along with central lifestyle.

To optimise holistic health outcomes, our study recommends focusing on central health outcomes and bridge lifestyles. For conditions such as COPD, eczema, fatty liver disease, gastric ulcer, heart attack, high cholesterol, hypertension, insomnia, and pre-diabetes, strategies like stress management, psychological counselling, and mindfulness can reduce emotional distress. Public health strategies should provide health care providers with training in mental health education and stress reduction techniques, enhancing support services and programmes. Quality of life improvements in anxiety, autoimmune disease, cancer, depression, diabetes, and irritable bowel syndrome require multi-disciplinary approaches focusing on symptom management and work-life balance. Bridge lifestyle interventions, such as increasing fruit and vegetable intake for managing diabetes, high cholesterol, hypertension, and insomnia, and advocating for reduced sitting duration and more exercise in conditions like eczema, fatty liver disease, heart attack, autoimmune disease, depression, anxiety, gastric ulcer, and insomnia, are crucial. These interventions align with the central lifestyle recommendations discussed earlier.

### Limitations

Our study acknowledges several limitations. First, the online recruitment approach, while facilitating international representation, may lead to selection bias. This potentially results in the underrepresentation of individuals with low socio-economic status and limited digital literacy. Additionally, the overrepresentation of female participants and underrepresentation of those over 65-year-old in our sample could also impact the generalisability of our findings. These factors suggest that our findings might not fully capture the diverse relationships between lifestyle and health outcomes across all demographics. Future studies should employ stratified sampling and incorporate offline data collection techniques to enhance demographic representativeness and ensure more robust validation of the findings. Second, our study's reliance on self-reported data poses a potential risk of information bias. While we mitigated this through validation questions and stringent data quality controls, it remains an inherent limitation of our methodology. Future research could enhance validity by utilising medical records and more rigorous lifestyle assessments, such as wearable technology or 24-hour recall, to corroborate our findings. Third, the cross-sectional design precludes us from accounting for the dynamics or evolution of networks over time. While the relationships and interactions between lifestyle factors and health outcomes would not frequently fluctuate, the key nodes may change over time. Further research utilising time-series data are needed to validate the permanence or phase-specific nature of the central positions of key nodes. Fourthly, our study, while offering initial insights into the relationships between lifestyle factors and health outcomes in chronic diseases, is exploratory in nature. It's important to note that our cross-sectional approach primarily provides associative, not causal, insights. The complexities and multifactorial aspects of chronic diseases mean that our findings should be interpreted cautiously. We acknowledge that further research, including longitudinal studies and randomised controlled trials, is essential to validate and deepen our understanding of these relationships. Lastly, the small sample sizes in several chronic disease groups restricted the interpretability of the networks and limited our ability to make robust comparisons, particularly regarding bridge lifestyles. Future research with larger sample sizes would help overcome this limitation.

## CONCLUSIONS

This study's exploration of central lifestyle, central health outcome, and bridge lifestyle across 16 chronic diseases provides initial insights into their potential roles in holistic health and lifestyle improvements. These key variables highlight the need for enhanced interventions and resource allocation. Moreover, recognising the shared key variables across chronic diseases can steer collaborative efforts in developing synergistic strategies. Specifically, in the most stringent scenario, when central lifestyle, central health outcome, and bridge lifestyle are targeted simultaneously, we can manage three disease groups, ie, hypertension and insomnia, gastric ulcer and insomnia, or eczema and heart attack.

## Additional material


Online Supplementary Document
