# Supplementary material for: Key lifestyles and health outcomes across 16 prevalent chronic diseases: A network analysis of an international observational study
Source: J Glob Health. 2024 Apr 12;14:04068. doi: 10.7189/jogh-14-04068 (PMC11010581; doi:10.7189/jogh-14-04068)
Supplement: Online Supplementary Document [file jogh-14-04068-s001.pdf]

ONLINE SUPPLEMENTARY DOCUMENT

Title: Key Lifestyles and Health Outcomes across 16 Prevalent Chronic Diseases: A Network Analysis of an International Observational Study

Authors: Jiaying Li, Daniel Yee Tak Fong, Kris Yuet Wan Lok, Janet Yuen Ha Wong, Mandy Man Ho, Edmond Pui Hang Choi, Vinciya Pandian, Patricia M Davidson, Wenjie Duan, Marie Tarrant, Jung Jae Lee, Chia-Chin Lin, Oluwadamilare Akingbade, Khalid M Alabdulwahhab, Mohammad Shakil Ahmad, Mohamed Alboraie, Meshari A Alzahrani, Anil S Bilimale, Sawitree Boonpatcharanon, Samuel Byiringiro, Muhammad Kamil Che Hasan, Luisa Clausi Schettini, Walter Corzo, Josephine M. De Leon, Anjanette S. De Leon, Hiba Deek, Fabio Efficace, Mayssah A El Nayal, Fathiya El-Raey, Eduardo Ensaldo-Carrasco, Pilar Escotorin, Oluwadamilola Agnes Fadodun, Israel Opeyemi Fawole, Yong-Shian Shawn Goh, Devi Irawan, Naimah Ebrahim Khan, Binu Koirala, Ashish Krishna, Cannas Kwok, Tung Thanh Le, Daniela Giambruno Leal, Miguel Ángel Lezana-Fernández, Emery Manirambona, Leandro Cruz Mantoani, Fernando Meneses-González, Iman Elmahdi Mohamed, Madeleine Mukeshimana, Chinh Thi Minh Nguyen, Huong Thi Thanh Nguyen, Khanh Thi Nguyen, Son Truong Nguyen, Mohd Said Nurumal, Aimable Nzabonimana, Nagla Abdelrahim Mohamed Ahmed Omer, Oluwabunmi Ogungbe, Angela Chiu Yin Poon, Areli Reséndiz-Rodriguez, Busayasachee Puang-Ngern, Ceryl G Sagun, Riyaz Ahmed Shaik, Nikhil Gauri Shankar, Kathrin Sommer, Edgardo Toro, Hanh Thi Hong Tran, Elvira L Urgel, Emmanuel Uwiringiyimana, Tita Vanichbuncha, Naglaa Youssef

Summary of supplementary material

STable or SFigure	Pages
**Table S1.** Description of means and standard deviations of lifestyle and health outcomes in each subgroup of chronic disease.	2-3
**Table S2.** Paired redundant items and removal reasons in Goldbricker analysis for each chronic disease subgroup.	4-5
**Table S3.** Partial correlation matrix of variables in lifestyles network across chronic disease subgroups.	6-13
**Table S4.** Partial correlation matrix of variables in health outcomes network across chronic disease subgroups.	14-21
**Table S5.** Partial correlation matrix of variables in bridge network of lifestyles and health outcomes across chronic disease subgroups.	22-39
**Figure S1.** Age and gender distribution of sample versus population across countries and overall	40-41
**Figure S2.** Bootstrapped confidence intervals of edge weights for the lifestyles network across chronic disease subgroups.	42-43
**Figure S3.** The stability of expected influence centrality index in lifestyles network across chronic disease subgroups using case-dropping bootstrap.	44-45
**Figure S4.** Network structure and centrality difference test of lifestyle (A and B), health outcomes (C and D), and combined two (E and F) across chronic disease subgroups.	46-61
**Figure S*5*.** Bootstrapped confidence intervals of edge weights for the health outcome network across chronic disease subgroups.	62-63
**Figure S6.** The stability of expected influence centrality index in health outcomes network across chronic disease subgroups using case-dropping bootstrap.	64-65
**Figure S7.** Bootstrapped confidence intervals of edge weights for the bridge network across chronic disease subgroups.	66-69
**Figure S8.** The stability of bridge expected influence centrality index in bridge network across chronic disease subgroups using case-dropping bootstrap.	70-71

Table S1. Description of means and standard deviations of lifestyle and health outcomes in each subgroup of chronic disease.

Lifestyles and health outcomes*	Mean (standard deviation)															
	Hypertension (n = 1509)	High cholesterol (n = 1283)	Pre-diabetes (n = 378)	Diabetes (n = 617)	Fatty liver disease (n = 542)	Heart attack (n = 533)	COPD (n = 340)	Cancer (n = 292)	Gastric ulcer (n = 579)	Irritable bowel syndrome (n = 533)	Insomnia (n = 722)	Depression (n = 981)	Anxiety (n = 1159)	Eczema (n = 681)	Autoimmune disease (n = 532)	Hearing problems (n = 217)
L1: Food types in daily meals	3.00(0.84)	2.97(0.85)	2.97(0.92)	2.97(0.81)	2.94(0.95)	3.00(0.93)	3.01(0.84)	2.92(0.85)	2.97(0.97)	3.00(0.93)	2.99(1.00)	3.03(0.99)	3.09(1.01)	2.90(0.84)	3.00(0.93)	2.97(0.80)
L2: Consumption of fruits and vegetables	3.14(0.85)	3.14(0.84)	3.15(0.98)	3.16(0.89)	3.17(0.88)	3.11(0.93)	3.12(0.92)	3.02(0.92)	3.12(0.97)	3.11(0.93)	3.14(1.01)	3.03(1.01)	3.11(1.01)	3.11(0.86)	3.11(0.93)	3.09(0.98)
L3: Less consumption of frozen food/food products	3.07(0.92)	3.16(0.92)	3.05(1.07)	3.04(0.94)	3.13(0.97)	2.97(0.99)	3.07(0.92)	3.07(0.95)	3.10(1.01)	2.97(0.99)	3.03(1.08)	3.08(1.00)	2.99(1.03)	3.23(0.94)	2.97(0.99)	3.05(0.99)
L4: Less consumption of snacks	2.90(0.94)	2.96(0.97)	2.89(1.06)	2.87(1.01)	2.88(1.03)	2.97(1.09)	2.99(1.08)	3.06(1.00)	2.97(1.01)	2.98(1.09)	3.03(1.09)	3.16(1.09)	3.11(1.14)	3.13(0.96)	2.98(1.09)	2.82(1.03)
L5: Less soft drinks/juices/other sugary drinks	2.74(0.97)	2.79(0.97)	2.72(1.12)	2.67(1.07)	2.72(1.02)	2.83(1.09)	2.75(1.08)	2.76(1.02)	2.85(1.07)	2.82(1.09)	2.93(1.14)	2.99(1.14)	2.92(1.17)	2.93(0.96)	2.83(1.09)	2.66(1.06)
L6: Having a meal at home	3.85(0.96)	3.93(0.92)	3.95(1.04)	3.79(0.94)	4.01(0.96)	4.07(0.98)	4.03(0.96)	3.93(0.94)	3.98(0.93)	4.07(0.98)	4.02(0.96)	4.04(1.00)	4.05(1.01)	4.07(0.91)	4.07(0.98)	3.80(0.95)
L7: Cooking at home	3.82(0.93)	3.87(0.91)	3.92(0.96)	3.84(0.90)	3.91(0.96)	3.92(1.05)	3.99(0.93)	3.99(0.87)	3.88(0.97)	3.92(1.05)	4.00(0.95)	3.99(1.01)	3.96(1.04)	3.96(0.91)	3.92(1.05)	3.85(0.96)
L8: Less eating takeout food	3.01(1.13)	3.08(1.13)	2.97(1.19)	2.94(1.13)	3.09(1.27)	2.90(1.26)	2.80(1.25)	3.04(1.20)	3.11(1.23)	2.90(1.26)	3.03(1.26)	3.05(1.26)	2.97(1.27)	3.10(1.24)	2.90(1.26)	2.90(1.18)
L9: Taking alternative medicine or natural health products	2.94(0.82)	2.92(0.81)	3.10(0.89)	2.85(0.82)	3.00(0.91)	3.03(0.96)	3.03(0.93)	2.87(0.85)	2.89(0.97)	3.03(0.96)	2.99(0.96)	2.99(0.94)	3.12(0.95)	2.95(0.79)	3.03(0.96)	2.95(0.85)
L10: Taking oral supplements/vitamins	3.15(0.83)	3.16(0.85)	3.25(0.88)	3.11(0.85)	3.23(0.91)	3.21(0.98)	3.17(0.99)	3.20(0.94)	3.15(0.98)	3.21(0.98)	3.21(0.98)	3.24(0.95)	3.31(0.97)	3.19(0.81)	3.21(0.98)	3.14(0.90)
L11: Less smoking tobacco	2.66(0.93)	2.68(0.91)	2.62(0.97)	2.71(0.92)	2.57(0.97)	2.65(0.99)	2.64(0.95)	2.73(0.88)	2.53(1.03)	2.65(0.99)	2.61(1.08)	2.75(1.03)	2.69(1.08)	2.72(0.83)	2.64(0.99)	2.67(0.94)
L12: Less alcohol consumption	2.65(0.94)	2.68(0.92)	2.81(0.98)	2.63(0.96)	2.53(1.02)	2.66(1.03)	2.71(1.00)	2.85(0.92)	2.58(1.03)	2.66(1.03)	2.65(1.08)	2.77(1.06)	2.72(1.10)	2.71(0.96)	2.66(1.03)	2.69(0.91)
L13: Less duration of sitting	3.61(0.90)	3.69(0.89)	3.85(1.00)	3.57(0.90)	3.70(0.93)	3.94(0.97)	3.96(0.94)	3.93(0.83)	3.63(0.98)	3.93(0.97)	3.94(0.98)	4.02(0.98)	4.05(0.97)	3.79(0.88)	3.94(0.97)	3.63(0.92)
L14: Less duration of screen time	3.65(0.88)	3.76(0.87)	3.89(0.99)	3.60(0.91)	3.82(0.89)	4.01(0.93)	3.95(0.97)	4.01(0.91)	3.74(0.99)	4.01(0.93)	3.98(0.96)	4.12(0.93)	4.15(0.95)	3.91(0.88)	4.01(0.93)	3.68(0.93)
L15: Frequency of exercise	2.70(1.05)	2.66(1.05)	2.53(1.10)	2.77(1.03)	2.51(1.09)	2.63(1.19)	2.70(1.15)	2.81(1.12)	2.76(1.14)	2.63(1.19)	2.66(1.24)	2.59(1.19)	2.62(1.26)	2.72(1.11)	2.63(1.20)	2.72(1.06)
L16: Duration of exercise	2.67(1.03)	2.65(1.02)	2.51(1.09)	2.76(1.02)	2.49(1.10)	2.62(1.18)	2.68(1.17)	2.78(1.10)	2.72(1.15)	2.62(1.18)	2.64(1.19)	2.58(1.19)	2.62(1.23)	2.71(1.07)	2.62(1.19)	2.69(1.02)
L17: Type of exercise	2.66(0.98)	2.64(0.97)	2.60(1.10)	2.73(0.95)	2.48(1.05)	2.64(1.16)	2.68(1.13)	2.80(1.10)	2.70(1.08)	2.64(1.16)	2.63(1.15)	2.59(1.17)	2.61(1.21)	2.68(1.05)	2.64(1.17)	2.66(0.98)
L18: Overall amount of exercise	2.64(1.02)	2.60(1.01)	2.53(1.06)	2.71(0.96)	2.49(1.10)	2.59(1.20)	2.64(1.13)	2.72(1.13)	2.73(1.13)	2.58(1.20)	2.60(1.20)	2.58(1.23)	2.59(1.26)	2.70(1.11)	2.58(1.20)	2.67(0.99)

H1: Lose weight	3.21(0.82)	3.30(0.80)	3.28(0.97)	3.14(0.88)	3.25(0.91)	3.22(0.99)	3.21(1.04)	3.32(0.88)	3.21(0.93)	3.22(0.99)	3.30(1.02)	3.33(1.06)	3.36(1.06)	3.22(0.89)	3.22(0.99)	3.22(0.87)
H2: Appetite	3.05(0.72)	3.13(0.72)	3.16(0.89)	3.01(0.77)	3.09(0.88)	3.18(0.93)	3.19(0.95)	3.17(0.82)	3.05(0.92)	3.18(0.93)	3.13(0.99)	3.18(1.01)	3.25(1.04)	3.12(0.83)	3.18(0.93)	3.10(0.81)
H3: Physical health	2.84(0.74)	2.81(0.74)	2.80(0.86)	2.85(0.77)	2.71(0.83)	2.80(0.90)	2.77(0.96)	2.71(0.87)	2.85(0.87)	2.80(0.90)	2.71(0.94)	2.67(0.96)	2.73(1.01)	2.82(0.80)	2.80(0.90)	2.80(0.80)
H4: Sleep quality	2.73(0.86)	2.73(0.85)	2.69(0.97)	2.73(0.85)	2.57(0.88)	2.68(1.06)	2.77(1.06)	2.65(0.93)	2.76(0.99)	2.68(1.06)	2.44(1.14)	2.55(1.13)	2.54(1.17)	2.69(0.91)	2.68(1.06)	2.72(0.90)
H5: Quality of life	2.58(0.90)	2.53(0.89)	2.61(0.96)	2.62(0.89)	2.39(0.92)	2.55(1.14)	2.55(1.06)	2.45(0.95)	2.60(1.03)	2.55(1.14)	2.35(1.06)	2.36(1.08)	2.43(1.13)	2.40(0.95)	2.55(1.14)	2.53(0.93)
H6: Less mental burden	3.35(1.03)	3.32(1.06)	3.62(1.10)	3.35(0.97)	3.35(1.13)	3.66(1.16)	3.54(1.13)	3.56(1.10)	3.33(1.16)	3.66(1.15)	3.58(1.30)	3.81(1.23)	3.93(1.23)	3.48(1.13)	3.66(1.16)	3.45(1.10)
H7: Less emotional distress	3.33(0.97)	3.29(1.01)	3.66(1.10)	3.31(0.94)	3.32(1.07)	3.58(1.16)	3.54(1.14)	3.55(1.09)	3.29(1.15)	3.58(1.16)	3.60(1.27)	3.84(1.21)	3.97(1.16)	3.43(1.09)	3.58(1.16)	3.42(1.08)
H8: Family disputes	3.06(0.78)	3.04(0.75)	3.19(0.86)	3.08(0.83)	2.96(0.81)	3.13(0.94)	3.19(0.90)	3.21(0.84)	3.07(0.93)	3.13(0.94)	3.30(1.02)	3.35(0.98)	3.38(1.01)	3.09(0.80)	3.13(0.94)	3.02(0.84)
H9: Social support provided	3.05(0.83)	3.03(0.81)	3.07(0.95)	3.00(0.83)	3.06(0.82)	3.10(0.96)	3.07(0.96)	2.98(0.92)	3.07(0.94)	3.10(0.96)	3.02(1.05)	2.98(1.07)	3.03(1.10)	3.05(0.85)	3.10(0.96)	2.96(0.93)
H10: Social support received	2.94(0.81)	2.96(0.81)	2.90(0.93)	2.93(0.83)	2.94(0.83)	2.84(0.94)	2.91(0.91)	2.98(0.90)	2.93(0.93)	2.84(0.94)	2.75(1.03)	2.77(1.04)	2.73(1.04)	2.94(0.87)	2.84(0.94)	2.87(0.90)
H11: Social activities	2.26(1.01)	2.20(0.99)	1.94(0.97)	2.33(0.98)	2.15(1.01)	2.02(1.14)	2.12(1.12)	2.02(1.00)	2.40(1.07)	2.02(1.14)	2.09(1.04)	1.97(1.08)	1.92(1.09)	2.12(0.97)	2.02(1.14)	2.21(1.03)
H12: Income	2.60(0.92)	2.62(0.90)	2.57(0.95)	2.67(0.89)	2.58(0.94)	2.62(0.99)	2.53(0.98)	2.66(0.86)	2.65(1.02)	2.62(0.99)	2.46(1.02)	2.46(1.01)	2.51(1.01)	2.56(0.90)	2.62(0.99)	2.73(0.84)
H13: Less economic burden	3.21(1.01)	3.17(0.99)	3.33(1.03)	3.26(0.97)	3.21(1.10)	3.30(1.07)	3.24(1.13)	3.16(1.01)	3.21(1.14)	3.30(1.07)	3.35(1.18)	3.40(1.13)	3.47(1.10)	3.26(1.02)	3.30(1.07)	3.12(0.95)

*Scored on a 5-point Likert scale: 1=Substantially reduced; 3= No change; 5=Substantially increased.

Table S2. Paired redundant items and removal reasons in Goldbricker analysis for each chronic disease subgroup.

(a) Paired redundant items and the removal.

Subgroup of chronic disease	Item redundancy for lifestyle items					Item redundancy for health outcome items		
	Round 1	Round 2	Round 3	Round 4	Round 5	Round 1	Round 2	Round 3
Hypertension	L15 & L17: remove L17	L15 & L16: remove L16	L11 & L12: both were remained	-	-	No suggested reductions	-	-
High cholesterol	L6 & L7: remove L7	L16 & L18: remove L16	L11 & L12: both were remained	-	-	No suggested reductions	-	-
Pre-diabetes	L6 & L7: remove L7	L15 & L16: remove L16	L15 & L17: remove L17	L15 & L18: remove L15	L11 & L12: both were remained	No suggested reductions	-	-
Diabetes	L15 & L18: remove L15	-	-	-	-	H6 & H7; remove H6	-	-
Fatty liver disease	L6 & L7: remove L7	L9 & L10: remove L9	L13 & L14: remove L14	L15 & L17: remove L17	-	No suggested reductions	-	-
Heart	L9 & L10: remove L9	L15 & L17: remove L17	L11 & L12: both were remained	-	-	No suggested reductions	-	-
Chronic obstructive pulmonary disease	L15 & L16: remove L16	L4 & L5: remove L4	L13 & L14: remove L14	L15 & L17: remove L17	-	H9 & H10: remove H9	H3 & H5: remove H3	H4 & H5: remove H4
Cancer	L15 & L16: remove L16	L15 & L18: remove L15	L17 & L18: remove L17	L11 & L12: both were remained	-	H6 & H7; remove H6	-	-
Gastric ulcer	L16 & L17: remove L17	L15 & L16: remove L16	L13 & L14: remove L14	L15 & L18: remove L15	-	No suggested reductions	-	-
Irritable bowel syndrome	L9 & L10: remove L9	L15 & L17: remove L17	L11 & L12: both were remained	-	-	H6 & H7; remove H6	-	-
Insomnia	L16 & L18: remove L16	L15 & L18: remove L15	-	-	-	No suggested reductions	-	-
Depression	L15 & L16: remove L16	L15 & L17: remove L17	L4 & L5: remove L4	L11 & L12: both were remained	-	No suggested reductions	-	-
Anxiety	L15 & L18: remove L15	L16 & L18: remove L16	-	-	-	No suggested reductions	-	-
Eczema	L11 & L12: both were remained	-	-	-	-	No suggested reductions	-	-
Autoimmune disease	L9 & L10: remove L9	L15 & L17: remove L17	L11 & L12: both were remained		-	H6 & H7; remove H6	-	-
Hearing problems	L6 & L7: remove L7	L4 & L5: remove L4	L15 & L18: remove L15	L11 & L12: both were remained	-	H6 & H7; remove H6	-	-

(b) Reason for the removals.

Removals	Reason for the removals
L4 (Less consumption of snacks) & L5 (Less soft drinks/juices/other sugary drinks): L4 was removed	The consumption of snacks (L4) does not necessarily imply an unhealthy lifestyle, as some snacks like nuts can be considered healthy. Therefore, choosing "less sugary drinks" (L5) is a more representative indicator of a healthier lifestyle.
L6 (Having a meal at home) & L7 (Cooking at home): L7 was removed	Having a meal at home (L6) is a more representative indicator of a healthier lifestyle than specifically focusing on cooking at home (L7). This is because individuals may not cook but still consume healthy food prepared by others.
L9 (Taking alternative medicine or natural health products) & L10 (Taking oral	Taking oral supplements/vitamins (L10) is more widely used and representative of healthier lifestyle practices compared to taking

supplements/vitamins): L9 was removed	alternative medicine or natural health products (L9).
L11 (Less smoking tobacco) & L12 (Less alcohol consumption): both remained	Both items were kept as they measure different objective aspects and do not have overlapping relationships.
L13 (Less duration of sitting) & L14 (Less duration of screen time): L14 was removed	Duration of sitting (L13) is a more representative measure of sedentary behavior, as screen time (L14) specifically captures sedentary behavior associated with the use of electronic devices.
L15 (Frequency of exercise) & L17 (Type of exercise): L17 was removed	
L15 (Frequency of exercise) & L18 (Overall amount of exercise): L15 was removed	Removal of physical activity items based on representativeness ranking: L18 > L15 > L16 > L17
L15(Frequency of exercise) & L16 (Duration of exercise): remove L16 was removed	L18 (overall amount of exercise) is the most comprehensive indicator of an individual's physical activity level. L15 (frequency) is a good indicator of routine exercise, where a higher frequency is better.
L16 (Duration of exercise) & L17 (Type of exercise): L17 was removed	L16 (duration) is a good indicator of the level of physical exertion, but longer duration is not always better.
L16 (Duration of exercise) & L18 (Overall amount of exercise): L16 was removed	L17 (type of exercise) mainly focuses on specific muscle training and is less representative of a complete picture of exercise.
L17(Type of exercise) & L18 (Overall amount of exercise): L17 was removed	
H6 (Less mental burden) & H7 (Less emotional distress): H6 was removed	Emotional distress (H7) is more widespread and common among individuals than mental burden (H6).
H3 (Physical health) & H5 (Quality of life): H3 was removed	Quality of life (H5) is a comprehensive and representative measure of better health and well-being.
H4 (Sleep quality) & H5 (Quality of life): H4 was removed	Quality of life (H5) is a comprehensive and representative measure of better health and well-being.
H9 (Social support provided) & H10 (Social support received): H9 was removed	Receiving social support (H10) is more closely related to an individual's health compared to providing social support (H9).

Table S3. Partial correlation matrix of variables in lifestyles network across chronic disease subgroups.

(a) Anxiety

	L1	L2	L3	L4	L5	L6	L7	L8	L9	L10	L11	L12	L13	L14	L17
L1															
L2	0.27095														
L3	0.00000	0.01028													
L4	-0.07293	0.06806	0.26749												
L5	-0.11526	0.03501	0.11551	0.40621											
L6	0.01370	0.03899	0.00000	-0.00168	0.00000										
L7	0.02327	0.07753	0.00000	0.00000	0.02094	0.65160									
L8	-0.04018	0.00000	0.02388	0.10111	0.12782	0.00000	0.04152								
L9	0.05837	0.07528	-0.01613	0.00000	0.01659	0.04921	0.00157	0.00000							
L10	0.02634	0.04970	-0.01582	0.00000	0.00000	0.05125	0.00000	-0.04863	0.38500						
L11	0.00000	0.14392	0.01407	0.00000	0.07631	0.00000	0.00000	0.00406	0.00000	-0.02366					
L12	-0.01503	0.00000	0.00000	0.10405	0.04442	0.00000	0.03007	0.10570	-0.00436	0.00000	0.48833				
L13	-0.02330	0.01738	0.00000	0.03081	0.00000	-0.04596	-0.01632	0.00000	-0.02088	0.00000	0.02357	0.00392			
L14	-0.00847	0.00000	0.06567	0.02021	0.06193	-0.06179	-0.01691	0.01696	0.00000	-0.01972	0.03770	0.00122	0.60677		
L17	0.00000	0.05174	0.01249	0.00000	0.04442	0.00000	0.00000	-0.01547	0.00000	0.00000	-0.03449	-0.01979	0.00691	0.00359	
L18	0.00000	0.04249	0.00000	0.00000	0.00000	-0.00684	0.00000	0.00000	-0.01075	0.00000	-0.00655	0.00000	0.02862	0.02413	0.81186

(b) Autoimmune disease

	L1	L2	L3	L4	L5	L6	L7	L8	L10	L11	L12	L13	L14	L15	L16
L1															
L2	0.3443														
L3	0.0000	0.0000													
L4	0.0000	0.0575	0.1652												
L5	-0.0176	0.0000	0.1316	0.4166											
L6	0.0031	0.0283	0.0000	0.0000	0.0000										
L7	0.0000	0.1602	0.0000	0.0000	0.0305	0.5682									
L8	0.0000	0.0000	0.1465	0.0086	0.1516	-0.0033	0.0773								
L10	0.0270	0.1104	-0.0166	0.0000	0.0000	0.0257	0.0000	0.0000							
L11	0.0000	0.0000	0.0400	0.0000	0.1388	0.0000	0.0000	0.0328	-0.0436						
L12	-0.0411	0.0000	0.0408	0.1375	0.0467	0.0338	0.0000	0.0000	-0.0480	0.4534					
L13	0.0000	0.0000	0.0000	0.0068	0.0294	-0.0128	0.0000	0.0000	-0.0317	0.0000	0.0172				
L14	0.0000	0.0000	0.0000	0.0480	0.0000	-0.1751	0.0000	0.0211	-0.0276	0.0036	0.0072	0.6562			
L15	0.0000	0.0000	0.0000	0.0000	0.0000	0.0000	0.0000	0.0000	0.0000	0.0000	0.0000	0.0000	0.0000		
L16	0.0006	0.0030	0.0000	0.0000	0.0000	0.0000	0.0000	0.0000	0.0000	0.0000	0.0000	0.0131	0.0000	0.4803	
L18	0.0075	0.0659	0.0125	0.0000	0.0000	0.0000	0.0000	0.0000	0.0000	0.0000	0.0000	0.0359	0.0000	0.3795	0.4999

(c) Cancer

	L1	L2	L3	L4	L5	L6	L7	L8	L9	L10	L11	L12	L13	L14
L1														
L2	0.1247													
L3	0.0000	0.0000												
L4	0.0000	0.0000	0.1510											
L5	0.0000	0.0374	0.0481	0.2573										
L6	0.0000	0.0000	0.0000	0.0000	0.0000									
L7	0.0000	0.0000	0.0000	0.0000	0.0000	0.2635								
L8	0.0000	0.0000	0.0000	0.0542	0.0076	0.0000	0.0000							
L9	0.0000	0.0297	0.0000	0.0000	0.0000	0.0000	0.0000	0.0000						
L10	0.0000	0.0000	0.0000	0.0000	0.0000	0.0000	0.0000	0.0000	0.2102					
L11	0.0000	0.0000	0.0000	0.0383	0.0183	0.0000	0.0000	0.0000	0.0000	0.0000				
L12	0.0000	0.0000	0.0000	0.0000	0.1605	0.0000	0.0000	0.0000	0.0000	0.0000	0.3377			
L13	0.0000	0.0000	0.0000	0.0000	0.0000	0.0000	0.0000	0.0000	0.0000	0.0000	0.0000	0.0000		
L14	0.0000	0.0000	0.0000	0.0000	0.0000	-0.0513	0.0000	0.0000	0.0000	0.0000	0.0000	0.0000	0.4297	
L18	0.0000	0.0000	0.0000	0.0000	0.0000	0.0000	0.0000	0.0000	0.0000	0.0000	0.0000	0.0000	0.0000	0.0000

(d) Chronic obstructive pulmonary disease

	L1	L2	L3	L5	L6	L7	L8	L9	L10	L11	L12	L13	L15
L1													
L2	0.2155												
L3	-0.0986	0.0000											
L5	-0.1090	0.0000	0.2289										
L6	0.0000	0.0152	0.0000	0.0044									
L7	0.0000	0.1075	0.0000	0.0000	0.6943								
L8	0.0000	0.0000	0.1459	0.0872	0.0000	0.0000							
L9	0.0534	0.0280	0.0000	0.0000	0.0000	0.0000	0.0000						
L10	0.0000	0.0000	-0.0123	-0.0585	0.0000	0.0000	0.0000	0.3000					
L11	0.0000	0.0232	0.0908	0.1082	0.0000	0.0942	0.0249	0.0000	0.0000				
L12	0.0000	0.0000	0.0000	0.0849	0.0000	0.0000	0.0770	0.0000	0.0000	0.5524			
L13	0.0000	0.0000	0.0113	0.0315	-0.0647	-0.0900	0.0000	-0.0213	-0.0076	0.0000	0.0000		
L15	-0.0154	0.0000	0.0000	0.0000	0.0000	0.0000	0.0000	0.0000	0.0000	0.0000	-0.0265	0.0000	
L18	0.0000	0.0000	0.0000	0.0000	0.0000	0.0000	0.0000	0.0000	0.0000	0.0000	0.0000	0.0287	0.7614

(e) Depression

	L1	L2	L3	L5	L6	L7	L8	L9	L10	L11	L12	L13	L14	L15
L1														
L2	0.2909													
L3	0.0000	0.0000												
L5	-0.0644	0.0610	0.2171											
L6	0.0144	0.0438	0.0000	0.0000										
L7	0.0163	0.0765	0.0000	0.0000	0.6186									
L8	-0.0068	0.0000	0.1378	0.1780	0.0000	0.0000								
L9	0.0114	0.0913	0.0000	0.0000	0.0242	0.0000	-0.0118							
L10	0.0148	0.0067	0.0000	0.0000	0.0329	0.0119	-0.0503	0.3277						
L11	0.0000	0.0677	0.0189	0.0843	0.0000	0.0000	0.0000	-0.0080	0.0000					
L12	0.0000	0.0000	0.0132	0.0923	0.0000	0.0000	0.1197	0.0000	0.0000	0.5027				
L13	-0.0182	0.0000	0.0000	0.0000	-0.0022	-0.0246	0.0000	-0.0168	0.0000	0.0249	0.0000			
L14	0.0000	0.0000	0.0154	0.0966	-0.1266	-0.0255	0.0049	0.0000	-0.0400	0.0107	0.0000	0.6006		
L15	0.0000	0.0894	0.0000	0.0112	0.0000	0.0000	0.0000	0.0000	0.0029	0.0000	0.0000	0.0436	0.0028	
L18	0.0000	0.0000	0.0036	0.0000	0.0000	0.0000	0.0000	0.0000	0.0000	-0.0266	0.0000	0.0182	0.0177	0.7820

(f) Diabetes

	L1	L2	L3	L4	L5	L6	L7	L8	L9	L10	L11	L12	L13	L14	L16	L17
L1																
L2	0.2725															
L3	-0.0341	0.0000														
L4	-0.0686	0.0263	0.1966													
L5	-0.0621	0.0340	0.0625	0.4235												
L6	0.0000	0.0233	-0.0523	0.0000	0.0000											
L7	0.0000	0.0806	0.0000	0.0000	0.0276	0.6333										
L8	-0.0226	0.0000	0.1402	0.2049	0.1037	0.0000	0.0000									
L9	0.0804	0.0784	-0.0314	-0.0025	0.0000	0.0000	0.0000	-0.0788								
L10	0.0000	0.1234	0.0000	0.0000	0.0000	0.0000	0.0571	0.0000	0.2319							
L11	0.0000	0.0000	0.0122	0.0000	0.0715	0.0180	0.0000	0.0295	0.0000	-0.0068						
L12	0.0000	0.0537	0.0000	0.0532	0.1064	0.0206	0.0000	0.0000	-0.0531	0.0000	0.5884					
L13	0.0000	0.0000	0.0000	0.0150	0.0000	-0.0356	0.0000	0.0000	0.0000	0.0000	0.0000	0.0000				
L14	0.0000	0.0000	0.0642	0.0292	0.0030	-0.0677	-0.0624	0.0292	-0.0007	-0.0480	0.0000	0.0380	0.5566			
L16	0.0000	0.0000	0.0000	0.0000	-0.0056	-0.0126	0.0000	0.0000	0.0000	0.0000	0.0000	-0.0478	0.0000	0.0000		
L17	0.0000	0.0000	0.0000	0.0000	-0.0149	0.0000	0.0000	0.0000	0.0000	0.0000	0.0000	-0.0409	0.0000	0.0000	0.3644	
L18	0.0000	0.0149	0.0000	0.0000	0.0000	0.0000	0.0000	0.0000	0.0000	0.0000	0.0000	0.0000	0.0150	0.0000	0.4558	0.4336

(g) Eczema

	L1	L2	L3	L4	L5	L6	L7	L8	L9	L10	L11	L12	L13	L14	L15	L16	L17
L1																	
L2	0.3759																
L3	0.0000	0.0000															
L4	-0.0395	0.0630	0.1745														
L5	-0.0213	0.0000	0.1716	0.3841													
L6	0.0055	0.0389	-0.0791	0.0000	0.0000												
L7	0.0000	0.0439	0.0000	0.0000	0.0299	0.6714											
L8	0.0000	0.0439	0.1671	0.0149	0.1659	0.0000	0.0232										
L9	0.0000	0.0827	-0.0306	0.0000	0.0000	0.0000	0.0000	0.0000									
L10	0.0259	0.0629	-0.1158	-0.0407	0.0000	0.0000	0.0575	0.0000	0.3843								
L11	0.0000	0.0000	0.0000	0.0000	0.0617	0.0000	0.0000	0.0043	0.0000	0.0000							
L12	-0.0343	0.0000	0.0000	0.0511	0.0311	0.0000	0.0286	0.0263	-0.0716	0.0000	0.4912						
L13	0.0000	0.0000	0.0229	0.0384	0.0000	0.0000	-0.0301	0.0000	-0.0147	-0.0014	0.0000	0.0267					
L14	0.0000	0.0000	0.0000	0.0957	0.0343	-0.0851	-0.0130	0.0087	0.0000	0.0000	0.0000	0.0000	0.5884				
L15	0.0334	0.0000	0.0000	0.0000	0.0000	0.0000	0.0000	0.0000	0.0000	0.0000	0.0000	0.0000	0.0000	0.0000			
L16	0.0000	0.0000	0.0000	0.0000	0.0154	0.0000	0.0000	0.0159	0.0000	0.0000	0.0000	0.0000	0.0000	0.0032	0.3564		
L17	0.0000	0.0218	0.0000	0.0000	0.0000	0.0000	0.0000	0.0000	0.0000	0.0000	-0.0039	-0.0201	0.0000	0.0062	0.0775	0.3496	
L18	0.0000	0.0200	0.0199	0.0000	0.0000	0.0000	0.0000	0.0000	0.0000	0.0000	0.0000	0.0000	0.0378	0.0151	0.3780	0.3022	0.3481

(h) Fatty liver disease

	L1	L2	L3	L4	L5	L6	L8	L10	L11	L12	L13	L15	L16
L1													
L2	0.2894												
L3	0.0000	0.0000											
L4	-0.1621	0.0000	0.2655										
L5	0.0000	0.0726	0.1043	0.4224									
L6	0.0269	0.2064	-0.0289	0.0000	0.0609								
L8	0.0000	0.0963	0.2468	0.0427	0.1654	-0.0564							
L10	0.0000	0.1305	-0.0048	0.0000	-0.0474	0.2227	-0.1157						
L11	0.0000	0.0075	0.0000	0.0393	0.0456	0.0327	0.0082	-0.0249					
L12	-0.0484	0.0000	0.0635	0.0579	0.0897	0.0000	0.0000	-0.0381	0.5297				
L13	-0.0076	-0.0130	0.0308	0.1007	0.0000	-0.2355	0.0000	-0.0601	0.0000	0.0507			
L15	0.0000	0.0138	0.0000	0.0064	0.0000	0.0000	0.0000	0.0000	0.0035	0.0000	0.0023		
L16	0.0000	0.0294	-0.0034	0.0000	0.0063	0.0000	-0.0354	0.0000	0.0000	-0.0087	0.0000	0.6013	
L18	0.0000	0.0000	0.0000	0.0000	0.0000	-0.0262	0.0000	0.0000	0.0000	-0.0211	0.0871	0.3859	0.4036

(i) Liver

	H1	H2	H3	H4	H5	H6	H7	H8	H9	H10	H11	H12
H1												
H2	-0.4061											
H3	0.0000	0.0000										
H4	0.0000	0.0000	0.2007									
H5	0.0000	0.0000	0.2573	0.3217								
H6	0.0000	-0.0792	0.0000	0.0000	-0.0210							
H7	0.0000	-0.0223	0.0000	0.0000	0.0000	0.6943						
H8	0.0000	-0.0378	0.0000	0.0000	0.0000	0.0190	0.1797					
H9	0.0000	0.0000	0.0000	0.0000	0.0000	-0.0385	-0.0229	-0.0282				
H10	0.0000	0.0000	0.0045	0.0629	0.0404	0.0000	0.0000	0.0000	0.3630			
H11	0.0000	0.0000	0.0319	0.0315	0.1755	0.0000	0.0706	0.0000	0.0061	0.1083		
H12	0.0000	-0.0172	-0.0338	0.0000	-0.0817	0.0000	0.0000	0.0000	-0.0233	-0.0518	-0.1024	
H13	0.0000	0.0000	0.0000	0.0000	0.0106	-0.1548	-0.0820	0.0000	0.0097	0.0000	0.0000	-0.1265

(j) Gastric ulcer

	L1	L2	L3	L4	L5	L6	L7	L8	L9	L10	L11	L12	L13
L1													
L2	0.3144												
L3	0.0000	0.0000											
L4	-0.0404	0.0000	0.1466										
L5	-0.0267	0.0000	0.1761	0.3468									
L6	0.0000	0.0067	0.0000	0.0000	0.0000								
L7	0.0172	0.0749	-0.0495	0.0000	0.0098	0.6818							
L8	-0.0111	0.0000	0.1625	0.1233	0.1195	0.0000	0.0000						
L9	0.0138	0.0000	-0.0863	0.0000	0.0000	0.0206	0.0000	0.0000					
L10	0.0471	0.0034	-0.1287	-0.0533	0.0000	0.0712	0.0000	-0.0906	0.2793				
L11	0.0000	0.0779	0.0000	0.0000	0.0897	0.0361	0.0000	0.0143	0.0000	-0.0177			
L12	-0.0542	0.0000	0.0000	0.0345	0.0838	0.0000	0.0169	0.0000	0.0000	-0.0058	0.6032		
L13	0.0000	0.0000	0.0409	0.0556	0.0341	-0.1398	-0.0293	0.0000	-0.0043	-0.0496	0.0288	0.0000	
L18	0.0000	0.1387	0.0000	0.0000	0.0000	0.0000	0.0000	0.0000	0.0000	0.0000	0.0000	-0.0405	0.0306

(k) Hearing problems

	L1	L2	L3	L5	L6	L8	L9	L10	L11	L12	L13	L14	L16	L17
L1														
L2	0.2016													
L3	-0.0324	0.0000												
L5	0.0000	0.0287	0.0089											
L6	0.0000	0.1175	0.0000	0.0000										
L8	0.0000	0.0000	0.0735	0.1652	0.0000									
L9	0.0000	0.0000	-0.0128	0.0000	0.0000	-0.0110								
L10	0.0000	0.0306	0.0000	0.0000	0.0000	0.0000	0.2080							
L11	0.0000	0.0000	0.0000	0.0651	0.0000	0.0000	0.0000	0.0000						
L12	0.0000	0.0000	0.0000	0.0915	0.0000	0.0529	0.0000	0.0000	0.4087					
L13	0.0000	0.0000	0.0000	0.0000	0.0000	0.0800	0.0000	0.0000	0.0000	0.0195				
L14	0.0000	0.0000	0.0000	0.0000	-0.1719	0.0578	0.0000	0.0000	0.0000	0.0000	0.4560			
L16	0.0000	0.0000	0.0000	0.0000	0.0000	0.0000	0.0000	0.0000	0.0000	-0.0516	0.0000	0.0000		
L17	0.0000	0.0000	0.0000	0.0000	0.0000	0.0000	0.0000	0.0000	0.0000	-0.0126	0.0000	0.0000	0.3504	
L18	0.0000	0.0000	0.0000	0.0000	0.0000	0.0000	0.0000	0.0000	0.0000	0.0000	0.0000	0.0000	0.4411	0.3509

(l) Heart attack

	L1	L2	L3	L4	L5	L6	L7	L8	L10	L11	L12	L13	L14	L15	L16	L18
L1																
L2	0.3455															
L3	0.0000	0.0000														
L4	0.0000	0.0539	0.1646													
L5	-0.0169	0.0000	0.1325	0.4147												
L6	0.0039	0.0289	0.0000	0.0000	0.0000											
L7	0.0000	0.1606	0.0000	0.0000	0.0310	0.5685										
L8	0.0000	0.0000	0.1466	0.0084	0.1519	-0.0032	0.0772									
L10	0.0271	0.1102	-0.0165	0.0000	0.0000	0.0259	0.0000	0.0000								
L11	0.0000	0.0000	0.0399	0.0000	0.1389	0.0000	0.0000	0.0328	-0.0435							
L12	-0.0403	0.0000	0.0412	0.1354	0.0480	0.0342	0.0000	0.0000	-0.0479	0.4536						
L13	0.0000	0.0000	0.0000	0.0008	0.0313	-0.0100	0.0000	0.0000	-0.0310	0.0000	0.0190					
L14	0.0000	0.0000	0.0000	0.0512	0.0000	-0.1767	0.0000	0.0210	-0.0280	0.0035	0.0062	0.6559				
L15	0.0000	0.0000	0.0000	0.0000	0.0000	0.0000	0.0000	0.0000	0.0000	0.0000	0.0000	0.0000	0.0000			
L16	0.0005	0.0029	0.0000	0.0000	0.0000	0.0000	0.0000	0.0000	0.0000	0.0000	0.0000	0.0130	0.0000	0.4803		
L18	0.0072	0.0655	0.0127	0.0000	0.0000	0.0000	0.0000	0.0000	0.0000	0.0000	0.0000	0.0358	0.0000	0.3795	0.5000	

(m) Hypertension

	L2	L3	L4	L5	L6	L7	L8	L9	L10	L11	L12	L13	L14	L15
L1														
L2														
L3	-0.0382													
L4	0.0671	0.2002												
L5	0.0769	0.1279	0.4169											
L6	0.0681	-0.0607	-0.0139	0.0000										
L7	0.0791	0.0000	0.0000	0.0657	0.6713									
L8	0.0373	0.2237	0.0921	0.1162	0.0000	0.0157								
L9	0.0505	-0.0084	0.0000	-0.0474	0.0071	0.0000	-0.0989							
L10	0.1248	-0.0423	-0.0101	0.0000	0.0180	0.0359	-0.0123	0.3645						
L11	0.0299	0.0384	0.0248	0.0963	0.0446	0.0000	0.0082	-0.0192	0.0000					
L12	0.0000	0.0059	0.0265	0.1246	0.0057	0.0059	0.0000	-0.0435	-0.0112	0.5361				
L13	0.0000	0.0000	0.0348	0.0000	-0.0200	-0.0564	0.0000	0.0000	-0.0353	0.0053	0.0373			
L14	0.0462	0.0358	0.0416	0.0014	-0.0907	-0.0508	0.0448	0.0000	-0.0288	0.0066	0.0291	0.6210		
L15	0.0033	0.0000	-0.0098	0.0000	0.0000	0.0000	0.0000	0.0101	0.0190	0.0000	-0.0197	0.0000	0.0000	
L18	0.0250	0.0000	0.0000	0.0000	-0.0202	0.0000	-0.0144	0.0004	0.0000	0.0000	-0.0134	0.0432	0.0299	0.8258

(n) Insomnia

	L1	L2	L3	L4	L5	L6	L7	L8	L9	L10	L11	L12	L13	L14	L17
L1															
L2	0.3032														
L3	-0.0276	-0.0298													
L4	-0.0780	0.0938	0.2600												
L5	-0.0548	0.0707	0.1014	0.4432											
L6	0.0000	0.0413	0.0000	0.0000	0.0184										
L7	0.0196	0.0925	0.0000	0.0000	0.0404	0.6013									
L8	0.0000	0.0099	0.1852	0.0758	0.1474	-0.0120	0.0000								
L9	0.0427	0.1466	0.0000	0.0000	0.0000	0.0404	0.0000	-0.0134							
L10	0.0000	0.0186	0.0000	0.0000	0.0000	0.0600	0.0130	-0.1061	0.3921						
L11	0.0000	0.0215	0.0000	0.0272	0.0235	0.0000	0.0000	0.0102	0.0110	0.0000					
L12	-0.0267	0.0764	0.0000	0.0000	0.0837	0.0000	0.0000	0.0786	0.0000	0.0000	0.5176				
L13	-0.0195	0.0442	0.0000	0.0017	0.0000	0.0000	-0.0936	0.0000	-0.0448	0.0000	0.0120	0.0726			
L14	0.0000	0.0000	0.0399	0.0000	0.1138	-0.1390	-0.0255	0.0173	0.0000	-0.0268	0.0000	0.0000	0.5828		
L17	0.0000	0.0319	0.0399	0.0000	0.0233	0.0000	0.0000	0.0000	-0.0107	0.0000	-0.0585	-0.0007	0.0000	0.0000	
L18	0.0000	0.0241	0.0000	0.0000	0.0000	-0.0243	0.0000	-0.0453	-0.0033	0.0000	-0.0271	-0.0233	0.0707	0.0000	0.7543

(o) Irritable bowel syndrome

	L1	L2	L3	L4	L5	L6	L7	L8	L10	L11	L12	L13	L14	L15	L16
L1															
L2	0.3420														
L3	0.0000	0.0000													
L4	0.0000	0.0534	0.1626												
L5	-0.0151	0.0000	0.1296	0.4170											
L6	0.0025	0.0251	0.0000	0.0000	0.0000										
L7	0.0000	0.1606	0.0000	0.0000	0.0299	0.5668									
L8	0.0000	0.0000	0.1476	0.0074	0.1500	-0.0010	0.0734								
L10	0.0260	0.1090	-0.0142	0.0000	0.0000	0.0246	0.0000	0.0000							
L11	0.0000	0.0000	0.0403	0.0000	0.1378	0.0000	0.0000	0.0328	-0.0425						
L12	-0.0391	0.0000	0.0415	0.1363	0.0469	0.0299	0.0000	0.0000	-0.0470	0.4521					
L13	0.0000	0.0000	0.0000	0.0084	0.0306	-0.0067	0.0000	0.0000	-0.0309	0.0000	0.0134				
L14	0.0000	0.0000	0.0000	0.0480	0.0000	-0.1734	0.0000	0.0164	-0.0277	0.0021	0.0066	0.6586			
L15	0.0000	0.0000	0.0000	0.0000	0.0000	0.0000	0.0000	0.0000	0.0000	0.0000	0.0000	0.0027	0.0000		
L16	0.0020	0.0000	0.0000	0.0000	0.0000	0.0000	0.0000	0.0000	0.0000	0.0000	0.0000	0.0214	0.0000	0.4853	
L18	0.0050	0.0698	0.0129	0.0000	0.0000	0.0000	0.0000	0.0000	0.0000	0.0000	0.0000	0.0225	0.0000	0.3763	0.4944

(p) Pre-diabetes

	L1	L2	L3	L4	L5	L6	L8	L9	L10	L11	L12	L13	L14
L1													
L2	0.1917												
L3	0.0000	0.0000											
L4	-0.1104	0.0472	0.0891										
L5	0.0000	0.0182	0.0565	0.4659									
L6	0.0000	0.1471	0.0000	0.0000	0.0000								
L8	0.0000	0.0000	0.1330	0.0642	0.1906	0.0000							
L9	0.0000	0.0000	0.0000	0.0000	0.0000	0.0180	0.0000						
L10	0.0000	0.0807	-0.0537	0.0000	0.0000	0.0240	0.0000	0.2893					
L11	0.0000	0.0967	0.0000	0.0045	0.0808	0.0000	0.0000	0.0000	0.0000				
L12	0.0000	0.0461	0.0262	0.0641	0.0698	0.0000	0.0700	-0.0275	0.0000	0.4317			
L13	0.0000	0.0000	0.0000	0.0000	0.0000	-0.0535	0.0000	0.0000	0.0000	0.0000	0.0417		
L14	0.0000	0.0000	0.0672	0.0373	0.0000	-0.1894	0.0000	0.0000	0.0000	0.0344	0.0000	0.5866	
L18	0.0000	0.0000	0.0320	0.0000	0.0000	0.0000	0.0000	0.0000	0.0000	0.0000	0.0000	0.0869	0.0362

Table S4. Partial correlation matrix of variables in health outcomes network across chronic disease subgroups.

(a) Anxiety

	H1	H2	H3	H4	H5	H6	H7	H8	H9	H10	H11	H12
H1												
H2	-0.47413											
H3	0.07506	0.03841										
H4	0.00000	0.02439	0.12442									
H5	0.00000	0.01384	0.30669	0.40557								
H6	0.01414	-0.01647	0.00000	-0.01718	0.00000							
H7	0.00000	-0.03752	0.00000	0.00000	0.00000	0.66075						
H8	0.00000	-0.03933	0.00000	0.00000	0.03173	0.05515	0.18054					
H9	0.00000	0.05345	0.00989	0.00000	0.07777	-0.00408	-0.01989	0.00000				
H10	0.00000	0.00000	0.00000	0.00000	0.04896	0.00000	0.00000	0.00000	0.37545			
H11	0.00000	0.00000	0.04273	0.07437	0.12709	0.00000	0.08735	-0.03225	0.05413	0.18711		
H12	0.00403	-0.00755	-0.01918	-0.00835	-0.12490	0.00000	0.00000	0.00000	-0.03841	-0.05974	-0.11815	
H13	0.00000	0.00000	0.00000	0.00000	-0.01700	-0.07044	-0.11786	-0.13913	0.03974	0.00000	0.04494	0.01413

(b) Autoimmune disease

	H1	H2	H3	H4	H5	H7	H8	H9	H10	H11	H12
H1											
H2	-0.3735										
H3	0.0000	0.0000									
H4	0.0000	0.0913	0.2200								
H5	0.0000	0.0000	0.2880	0.2955							
H7	0.0000	-0.0397	0.0000	0.0000	0.0000						
H8	0.0000	-0.0493	0.0000	0.0000	0.0514	0.3399					
H9	0.0000	0.0000	0.0000	0.0000	0.0293	-0.0249	0.0000				
H10	0.0000	0.0000	0.0397	0.0013	0.0000	0.0000	0.0000	0.3791			
H11	0.0000	0.0000	0.0000	0.1251	0.2035	0.0000	0.0000	0.0217	0.0963		
H12	0.0000	0.0000	-0.0194	0.0000	-0.0876	0.0369	0.0000	0.0000	-0.0402	-0.0330	
H13	0.0000	0.0000	0.0000	0.0000	0.0000	-0.1661	0.0000	0.0000	0.0000	0.0000	0.0000

(c) Cancer

	H1	H2	H3	H4	H5	H7	H8	H9	H10	H11	H12
H1											
H2	-0.3499										
H3	0.0000	0.0000									
H4	0.0000	0.0000	0.0990								
H5	0.0000	0.0000	0.2626	0.2300							
H7	0.0088	0.0000	0.0000	0.0000	0.0000						
H8	0.1357	-0.0237	0.0000	0.0000	0.0000	0.2144					
H9	0.0000	0.0000	0.0078	0.0000	0.1074	0.0000	0.0000				
H10	0.0000	0.0355	0.0000	0.0000	0.0000	0.0000	0.0000	0.2998			
H11	0.0000	0.0000	0.0000	0.0465	0.1570	0.0000	0.0000	0.0000	0.0557		
H12	0.0000	0.0000	-0.1064	0.0000	-0.0481	0.0000	0.0000	0.0000	-0.0245	-0.0957	
H13	0.0000	0.0000	0.0000	0.0000	0.0000	-0.1321	-0.0435	0.0000	0.0000	0.0000	0.0000

(d) Chronic obstructive pulmonary disease

	H1	H2	H5	H6	H7	H8	H10	H11	H12
H1									
H2	-0.2766								
H5	0.0000	0.0000							
H6	0.0000	0.0000	0.0000						
H7	0.0000	0.0000	0.0000	0.5087					
H8	0.0000	0.0000	0.0000	0.0728	0.2463				
H10	0.0000	0.0000	0.0000	0.0000	0.0000	-0.0275			
H11	0.0000	0.0000	0.2302	0.0000	0.0000	0.0000	0.1663		
H12	0.0000	0.0000	-0.0785	0.0000	0.0000	0.0000	-0.0420	-0.0678	
H13	0.0000	0.0000	0.0000	-0.0873	0.0000	-0.0291	0.0524	0.0000	0.0000

(e) Depression

	H1	H2	H3	H4	H5	H6	H7	H8	H9	H10	H11	H12
H1												
H2	-0.4502											
H3	0.0608	0.0105										
H4	0.0000	0.0445	0.1477									
H5	0.0000	0.0000	0.3154	0.3299								
H6	0.0000	0.0000	0.0000	-0.0028	0.0000							
H7	0.0000	-0.0223	-0.0187	0.0000	0.0000	0.6950						
H8	0.0000	-0.0237	0.0000	0.0000	0.0000	0.0382	0.1828					
H9	0.0000	0.0000	0.0000	0.0000	0.0625	-0.0317	0.0000	0.0000				
H10	0.0000	0.0000	0.0000	0.0000	0.0474	0.0000	0.0000	0.0000	0.3393			
H11	0.0000	0.0000	0.0283	0.0689	0.1605	0.0091	0.0563	0.0000	0.0821	0.1461		
H12	0.0000	0.0000	-0.0515	-0.0296	-0.0803	0.0000	0.0000	0.0000	-0.0250	-0.0278	-0.1439	
H13	0.0000	0.0000	0.0000	0.0000	0.0000	-0.0938	-0.0789	-0.0964	0.0000	0.0000	0.0000	0.0000

(f) Diabetes

	H1	H2	H3	H4	H5	H7	H8	H9	H10	H11	H12
H1											
H2	-0.4950										
H3	0.0000	0.0487									
H4	0.0000	0.1071	0.1807								
H5	0.0780	0.0000	0.2698	0.3378							
H7	0.0000	0.0000	0.0000	0.0000	0.0000						
H8	0.0391	-0.0781	0.0000	0.0000	0.0360	0.3529					
H9	0.0334	0.0000	0.0212	0.0000	0.0800	0.0000	0.0000				
H10	0.0000	0.0000	0.0104	0.0089	0.0376	0.0000	0.0000	0.2803			
H11	0.0000	0.0000	0.0000	0.0454	0.1031	0.0507	0.0000	0.0107	0.0895		
H12	0.0000	0.0000	-0.1028	0.0000	-0.1339	-0.0204	-0.0403	-0.0159	-0.0885	-0.1529	
H13	0.0000	0.0429	0.0000	0.0000	0.0000	-0.2328	-0.0812	0.0651	0.0000	0.0000	0.0190

(g) Eczema

	H1	H2	H3	H4	H5	H6	H7	H8	H9	H10	H11	H12
H1												
H2	-0.3882											
H3	0.0000	0.0000										
H4	0.0000	0.0000	0.2179									
H5	0.0000	0.0000	0.1834	0.2826								
H6	0.0000	0.0000	0.0000	0.0000	0.0000							
H7	0.0000	0.0000	0.0000	0.0000	0.0000	0.6690						
H8	0.0323	0.0000	0.0000	0.0000	0.0000	0.0325	0.1670					
H9	0.0000	0.0000	0.0000	0.0000	0.0611	0.0000	0.0000	0.0000				
H10	0.0000	0.0000	0.0030	0.0000	0.0000	0.0000	0.0000	0.0000	0.3005			
H11	0.0292	0.0000	0.0259	0.0210	0.1982	0.0378	0.0000	0.0000	0.0228	0.1021		
H12	-0.0203	0.0000	-0.0332	0.0000	-0.0792	0.0000	0.0000	-0.0037	0.0000	-0.0764	-0.0909	
H13	0.0000	0.0000	0.0000	0.0000	0.0000	-0.0855	-0.0943	-0.0409	0.0000	0.0000	0.0000	0.0000

(h) Liver

	H1	H2	H3	H4	H5	H6	H7	H8	H9	H10	H11	H12
H1												
H2	-0.4061											
H3	0.0000	0.0000										
H4	0.0000	0.0000	0.2007									
H5	0.0000	0.0000	0.2573	0.3217								
H6	0.0000	-0.0792	0.0000	0.0000	-0.0210							
H7	0.0000	-0.0223	0.0000	0.0000	0.0000	0.6943						
H8	0.0000	-0.0378	0.0000	0.0000	0.0000	0.0190	0.1797					
H9	0.0000	0.0000	0.0000	0.0000	0.0000	-0.0385	-0.0229	-0.0282				
H10	0.0000	0.0000	0.0045	0.0629	0.0404	0.0000	0.0000	0.0000	0.3630			
H11	0.0000	0.0000	0.0319	0.0315	0.1755	0.0000	0.0706	0.0000	0.0061	0.1083		
H12	0.0000	-0.0172	-0.0338	0.0000	-0.0817	0.0000	0.0000	0.0000	-0.0233	-0.0518	-0.1024	
H13	0.0000	0.0000	0.0000	0.0000	0.0106	-0.1548	-0.0820	0.0000	0.0097	0.0000	0.0000	-0.1265

(i) Gastric ulcer

	H1	H2	H3	H4	H5	H6	H7	H8	H9	H10	H11	H12
H1												
H2	-0.4042											
H3	-0.0395	0.1447										
H4	0.0000	0.0119	0.2138									
H5	0.0000	0.0000	0.2612	0.2882								
H6	0.0307	0.0000	0.0000	-0.0097	-0.0566							
H7	0.0000	0.0000	-0.0166	0.0000	0.0000	0.6798						
H8	0.0684	-0.0542	0.0000	0.0000	0.0253	0.0132	0.2570					
H9	0.0000	0.0000	0.1061	0.0000	0.0265	-0.0470	0.0000	-0.0849				
H10	0.0000	0.0306	0.0054	0.0000	0.0000	0.0000	0.0000	0.0000	0.3906			
H11	0.0234	0.0000	0.0600	0.0467	0.1499	0.0000	0.0197	0.0000	0.0374	0.1209		
H12	0.0000	0.0000	0.0000	0.0000	-0.1625	0.0000	0.0291	-0.0502	-0.0552	-0.0299	-0.1568	
H13	0.0000	0.0094	0.0000	0.0000	0.0628	-0.1128	-0.0895	-0.1017	0.0551	0.0000	0.0000	-0.0839

(j) Hearing problems

	H1	H2	H3	H4	H5	H7	H8	H9	H10	H11	H12
H1											
H2	-0.3454										
H3	0.0449	0.0000									
H4	0.0000	0.0985	0.1245								
H5	0.0000	0.0000	0.1138	0.2500							
H7	0.0000	0.0000	0.0000	0.0000	0.0000						
H8	0.0000	0.0000	0.0000	0.0000	0.0744	0.2415					
H9	0.0000	0.0000	0.1355	0.0000	0.1271	0.0000	0.0000				
H10	0.0000	0.0000	0.0000	0.0000	0.0000	0.0000	0.0000	0.2128			
H11	0.0000	0.0000	0.0000	0.1245	0.0928	0.0000	0.0000	0.0000	0.1751		
H12	0.0000	0.0000	-0.0516	-0.0258	0.0000	0.0000	0.0000	-0.0795	-0.1186	-0.0220	
H13	0.0000	0.0000	0.0000	0.0000	0.0000	-0.0970	-0.0976	0.0000	0.0000	0.0000	0.0000

(k) Heart attack

	H1	H2	H3	H4	H5	H6	H7	H8	H9	H10	H11	H12	H13
H1													
H2	-0.3705												
H3	0.0000	0.0000											
H4	0.0000	0.0868	0.2171										
H5	0.0000	0.0000	0.2872	0.2951									
H6	0.0000	0.0000	0.0000	0.0000	0.0000								
H7	0.0000	-0.0284	0.0000	0.0000	0.0000	0.6508							
H8	0.0000	-0.0475	0.0000	0.0000	0.0489	0.0062	0.2523						
H9	0.0000	0.0000	0.0000	0.0000	0.0283	-0.0204	-0.0036	0.0000					
H10	0.0000	0.0000	0.0390	0.0000	0.0000	0.0000	0.0000	0.0000	0.3768				
H11	0.0000	0.0000	0.0000	0.1261	0.2026	0.0000	0.0000	0.0000	0.0210	0.0950			
H12	0.0000	0.0000	-0.0186	0.0000	-0.0862	0.0232	0.0103	0.0000	0.0000	-0.0386	-0.0310		
H13	0.0000	0.0000	0.0000	0.0000	0.0000	-0.1493	-0.0260	0.0000	0.0000	0.0000	0.0000	0.0000	

(l) High cholesterol

	H1	H2	H3	H4	H5	H6	H7	H8	H9	H10	H11	H12
H1												
H2	-0.3590											
H3	0.0000	0.0488										
H4	0.0000	0.0000	0.2241									
H5	0.0000	0.0000	0.2322	0.3685								
H6	0.0292	-0.0124	0.0000	0.0000	-0.0106							
H7	0.0242	0.0000	0.0000	0.0000	0.0000	0.6497						
H8	0.0525	-0.0845	0.0000	0.0000	0.0000	0.0286	0.1976					
H9	0.0000	0.0131	0.0223	0.0000	0.0476	-0.0447	0.0000	0.0000				
H10	0.0000	0.0000	0.0218	0.0000	0.0000	0.0000	-0.0087	-0.0452	0.3509			
H11	0.0075	0.0000	0.0000	0.0824	0.1500	0.0121	0.0511	0.0000	0.0150	0.0628		
H12	0.0000	0.0000	-0.0448	-0.0012	-0.0778	0.0000	0.0000	-0.0263	-0.0606	-0.0388	-0.1161	
H13	0.0000	0.0223	0.0000	0.0000	0.0000	-0.1444	-0.0572	-0.0579	0.0337	0.0000	0.0000	0.0000

(m) Hypertension

	H1	H2	H3	H4	H5	H6	H7	H8	H9	H10	H11	H12
H1												
H2	-0.4602											
H3	0.0000	0.0677										
H4	0.0000	0.0882	0.2185									
H5	0.0285	0.0000	0.2543	0.3342								
H6	0.0000	-0.0358	0.0000	0.0000	-0.0234							
H7	0.0205	0.0000	0.0000	0.0000	0.0000	0.6292						
H8	0.0280	-0.0710	0.0000	0.0000	0.0366	0.0220	0.2138					
H9	0.0000	0.0357	0.0122	0.0000	0.0468	-0.0533	-0.0097	-0.0242				
H10	0.0000	0.0000	0.0000	0.0000	0.0000	0.0000	0.0000	-0.0267	0.4030			
H11	0.0000	0.0000	0.0000	0.0900	0.1496	0.0000	0.0589	0.0000	0.0391	0.1334		
H12	0.0000	0.0000	-0.0577	-0.0125	-0.1784	0.0000	-0.0129	-0.0533	0.0000	-0.0829	-0.1270	
H13	-0.0486	0.0000	0.0000	0.0000	0.0000	-0.1307	-0.1043	-0.0814	0.0566	0.0000	0.0000	0.0000

(n) Insomnia

	H1	H2	H3	H4	H5	H6	H7	H8	H9	H10	H11	H12
H1												
H2	-0.4349											
H3	0.0000	0.0399										
H4	0.0000	0.0478	0.1203									
H5	0.0000	0.0000	0.3368	0.3642								
H6	0.0000	-0.0304	0.0000	0.0000	-0.0376							
H7	0.0000	0.0000	-0.0037	0.0000	0.0000	0.6591						
H8	0.0000	-0.0292	0.0000	-0.0273	0.0000	0.0561	0.2225					
H9	0.0000	0.0145	0.0000	0.0000	0.0565	-0.0760	-0.0048	0.0000				
H10	0.0000	0.0000	0.0100	0.0000	0.0000	0.0000	0.0000	0.0000	0.3090			
H11	0.0000	0.0000	0.0373	0.0870	0.0983	0.0230	0.0138	0.0000	0.0299	0.2217		
H12	0.0000	-0.0147	-0.1137	0.0000	-0.1307	0.0168	0.0000	-0.0097	-0.0847	-0.0430	-0.1384	
H13	-0.0320	0.0097	0.0000	0.0000	0.0000	-0.0863	-0.0807	-0.1719	0.0000	0.0000	0.0560	0.0000

(o) Irritable bowel syndrome

	H1	H2	H3	H4	H5	H7	H8	H9	H10	H11	H12
H1											
H2	-0.3734										
H3	0.0000	0.0000									
H4	0.0000	0.0916	0.2215								
H5	0.0000	0.0000	0.2867	0.2949							
H7	0.0000	-0.0396	0.0000	0.0000	0.0000						
H8	0.0000	-0.0495	0.0000	0.0000	0.0517	0.3399					
H9	0.0000	0.0000	0.0000	0.0000	0.0296	-0.0255	0.0000				
H10	0.0000	0.0000	0.0395	0.0013	0.0000	0.0000	0.0000	0.3790			
H11	0.0000	0.0000	0.0000	0.1236	0.2046	0.0000	0.0000	0.0230	0.0960		
H12	0.0000	0.0000	-0.0186	0.0000	-0.0881	0.0372	0.0000	0.0000	-0.0403	-0.0335	
H13	0.0000	0.0000	0.0000	0.0000	0.0000	-0.1660	0.0000	0.0000	0.0000	0.0000	0.0000

(p) Pre-diabetes

	H1	H2	H3	H4	H5	H6	H7	H8	H9	H10	H11	H12
H1												
H2	-0.3655											
H3	0.0000	0.0000										
H4	0.0000	0.0000	0.1258									
H5	0.0000	0.0000	0.2237	0.2126								
H6	0.0000	-0.0027	0.0000	0.0000	0.0000							
H7	0.0000	-0.0383	0.0000	0.0000	0.0000	0.5404						
H8	0.0000	0.0000	0.0000	0.0000	0.0000	0.0225	0.2254					
H9	0.0000	0.0000	0.0584	0.0000	0.0234	0.0000	0.0000	0.0000				
H10	0.0000	0.0000	0.0000	0.0000	0.0279	0.0000	0.0000	0.0000	0.3177			
H11	0.0000	0.0000	0.0000	0.0734	0.1319	0.0000	0.0678	0.0000	0.0313	0.0000		
H12	0.0000	0.0000	0.0000	0.0000	-0.1100	0.0000	0.0000	0.0000	0.0000	0.0000	-0.0288	
H13	0.0000	0.0000	0.0000	0.0000	0.0000	-0.0713	-0.0653	-0.0188	0.0000	0.0000	0.0000	0.0000

Table S5. Partial correlation matrix of variables in bridge network of lifestyles and health outcomes across chronic disease subgroups.

(a) Anxiety

	L1	L2	L3	L4	L5	L6	L7	L8	L9	L10	L11	L12	L13	L14	L17	L18	H1	H2	H3	H4	H5	H6	H7	H8	H9	H10	H11	H12
L1																												
L2	0.2187																											
L3	0.0000	0.0000																										
L4	-0.0308	0.0456	0.2531																									
L5	-0.0804	0.0239	0.1141	0.3885																								
L6	0.0089	0.0296	0.0000	0.0000	0.0000																							
L7	0.0051	0.0725	0.0000	0.0000	0.0000	0.6199																						
L8	-0.0030	0.0000	0.0114	0.0900	0.1184	0.0000	0.0130																					
L9	0.0397	0.0578	0.0000	0.0000	0.0000	0.0421	0.0000	0.0000																				
L10	0.0180	0.0264	0.0000	0.0000	0.0000	0.0402	0.0000	-0.0166	0.3520																			
L11	0.0000	0.1111	0.0032	0.0053	0.0706	0.0000	0.0000	0.0000	0.0000	0.0000																		
L12	0.0000	0.0000	0.0000	0.0929	0.0406	0.0000	0.0111	0.0916	0.0000	0.0000	0.4628																	
L13	0.0000	0.0000	0.0000	0.0234	0.0000	-0.0413	-0.0077	0.0000	-0.0011	0.0000	0.0105	0.0000																
L14	0.0000	0.0000	0.0562	0.0175	0.0536	-0.0543	-0.0051	0.0000	0.0000	-0.0056	0.0261	0.0000	0.5690															
L17	0.0000	0.0250	0.0000	0.0000	0.0205	0.0000	0.0000	0.0000	0.0000	0.0000	-0.0142	-0.0029	0.0000	0.0000														
L18	0.0000	0.0098	0.0000	0.0000	0.0000	0.0000	0.0000	0.0000	0.0000	0.0000	0.0000	0.0000	0.0149	0.0142	0.7485													
H1	-0.0432	0.0000	0.0000	0.0184	0.0594	0.0000	0.0000	0.0059	0.0000	0.0000	0.0000	0.0035	0.0149	0.0399	0.0065	0.0586												
H2	0.1754	0.0000	-0.0005	-0.0794	0.0000	0.0045	0.0000	-0.0599	0.0000	0.0000	0.0000	-0.0327	-0.0072	-0.0097	0.0000	0.0000	-0.3964											
H3	0.0117	0.1282	0.0000	0.0000	0.0000	0.0000	0.0000	0.0000	0.0000	0.0000	0.0000	0.0000	0.0318	0.0000	0.0797	0.1050	0.0000	0.0000										
H4	0.0000	0.0000	0.0000	0.0000	0.0000	0.0000	0.0000	0.0000	0.0000	-0.0105	0.0000	0.0000	0.0000	0.0000	0.0000	0.0000	0.0000	0.0093	0.1111									
H5	0.0015	0.0384	0.0000	0.0000	0.0000	0.0000	0.0000	0.0000	0.0000	0.0000	0.0000	0.0000	0.0168	0.0000	0.0110	0.0308	0.0000	0.0045	0.2548	0.3860								
H6	0.0000	0.0000	0.0000	0.0000	0.0000	0.0000	0.0000	0.0000	0.0000	0.0000	0.0000	0.0155	0.0252	0.0245	0.0000	0.0000	0.0000	-0.0087	0.0000	0.0000	0.0000							
H7	-0.0412	0.0000	0.0000	0.0000	0.0000	-0.0091	0.0000	0.0000	-0.0295	-0.0188	0.0000	0.0000	0.0091	0.0445	0.0000	0.0000	0.0000	-0.0137	0.0000	0.0000	0.0000	0.6237						
H8	0.0000	0.0000	0.0000	0.0060	0.0000	0.0000	0.0000	0.0000	0.0000	0.0000	0.0000	0.0000	0.0231	0.0207	0.0000	0.0000	0.0000	-0.0125	0.0000	0.0000	0.0000	0.0515	0.1655					
H9	0.0000	0.0000	0.0000	0.0000	0.0000	0.0000	0.0000	0.0000	0.0466	0.0098	0.0000	0.0000	0.0000	0.0000	0.0000	0.0000	0.0000	0.0301	0.0000	0.0000	0.0714	0.0000	0.0000	0.0000				
H10	0.0000	0.0000	-0.0153	0.0000	0.0000	0.0000	0.0000	-0.0157	0.0000	0.0469	0.0000	-0.0212	0.0000	0.0000	0.0000	0.0211	0.0000	0.0000	0.0000	0.0000	0.0385	0.0000	0.0000	0.0000	0.3477			

H11	0.0000	0.0000	0.0000	0.0000	0.0000	-0.0475	0.0000	0.0000	0.0000	0.0000	0.0000	0.0000	0.0053	0.0190	0.0015	0.0504	0.0000	0.0000	0.0086	0.0631	0.1191	0.0000	0.0364	0.0000	0.0452	0.1694		
H12	0.0000	0.0000	0.0000	0.0000	0.0000	0.0000	0.0055	0.0462	0.0000	0.0000	0.0000	0.0000	0.0000	0.0000	0.0000	0.0000	0.0000	0.0000	-0.0061	0.0000	-0.1190	0.0000	0.0000	0.0000	-0.0252	-0.0467	-0.1024	
H13	0.0000	0.0000	0.0000	0.0000	0.0000	0.0000	0.0000	0.0000	0.0000	0.0000	0.0000	0.0000	0.0000	0.0000	0.0000	0.0000	0.0000	0.0000	0.0000	0.0000	0.0000	-0.0646	-0.1077	-0.1201	0.0155	0.0000	0.0000	0.0000

(b) Autoimmune disease

	L1	L2	L3	L4	L5	L6	L7	L8	L10	L11	L12	L13	L14	L15	L16	L18	H1	H2	H3	H4	H5	H7	H8	H9	H10	H11	H12
L1																											
L2	0.3200																										
L3	0.0000	0.0000																									
L4	0.0000	0.0429	0.1545																								
L5	0.0000	0.0000	0.1280	0.3959																							
L6	0.0000	0.0226	0.0000	0.0000	0.0000																						
L7	0.0000	0.1523	0.0000	0.0000	0.0184	0.5459																					
L8	0.0000	0.0000	0.1319	0.0000	0.1424	0.0000	0.0540																				
L10	0.0131	0.0950	0.0000	0.0000	0.0000	0.0126	0.0000	0.0000																			
L11	0.0000	0.0000	0.0360	0.0000	0.1341	0.0000	0.0000	0.0227	-0.0310																		
L12	-0.0080	0.0000	0.0354	0.1237	0.0475	0.0094	0.0000	0.0000	-0.0373	0.4361																	
L13	0.0000	0.0000	0.0000	0.0000	0.0152	-0.0071	0.0000	0.0000	-0.0202	0.0000	0.0023																
L14	0.0000	0.0000	0.0000	0.0236	0.0000	-0.1655	0.0000	0.0000	-0.0223	0.0000	0.0000	0.6181															
L15	0.0000	0.0000	0.0000	0.0000	0.0000	0.0000	0.0000	0.0000	0.0000	0.0000	0.0000	0.0000	0.0000														
L16	0.0000	0.0005	0.0000	0.0000	0.0000	0.0000	0.0000	0.0000	0.0000	0.0000	0.0000	0.0060	0.0000	0.4613													
L18	0.0000	0.0598	0.0013	0.0000	0.0000	0.0000	0.0000	0.0000	0.0000	0.0000	0.0000	0.0263	0.0000	0.3728	0.4802												
H1	-0.0218	0.0000	0.0062	0.0506	0.0316	0.0000	0.0000	0.0535	0.0000	0.0000	0.0202	0.0431	0.0381	0.0347	0.0000	0.0000											
H2	0.1502	0.0000	-0.0075	-0.1054	0.0000	0.0000	0.0000	-0.0126	0.0000	0.0000	-0.0260	-0.0603	-0.0224	0.0000	0.0000	0.0000	-0.3260										
H3	0.0095	0.0294	0.0000	0.0000	0.0000	0.0000	0.0000	0.0000	0.0000	0.0000	0.0000	0.0000	0.0000	0.0199	0.0374	0.0514	0.0000	0.0019									
H4	0.0000	0.0000	0.0000	0.0000	0.0000	0.0000	0.0000	0.0000	-0.0284	0.0000	0.0000	0.0000	0.0000	0.0063	0.0145	0.0000	0.0000	0.0921	0.2060								
H5	0.0031	0.0000	0.0000	0.0000	0.0000	0.0000	0.0000	0.0000	0.0000	0.0198	0.0000	0.0356	0.0000	0.0449	0.0000	0.0364	0.0000	0.0000	0.2498	0.2883							
H7	-0.0134	0.0000	0.0000	0.0000	0.0000	0.0000	0.0000	0.0000	0.0000	0.0128	0.0000	0.0257	0.0397	0.0000	0.0000	0.0000	0.0000	-0.0221	0.0000	0.0000	0.0000						
H8	0.0000	0.0000	0.0000	0.0476	0.0000	0.0000	0.0000	0.0000	0.0000	0.0000	0.0000	0.0842	0.0192	0.0000	0.0000	0.0000	0.0000	-0.0169	0.0000	0.0000	0.0351	0.3218					

H9	0.0000	0.0000	0.0000	0.0000	0.0000	0.0000	0.0000	0.0000	0.0000	0.0000	0.0000	0.0000	0.0000	0.0000	0.0000	0.0000	0.0000	0.0000	0.0000	0.0000	0.0246	-0.0218	0.0000				
H10	0.0000	0.0000	0.0000	0.0000	0.0000	0.0000	0.0102	0.0000	0.0250	0.0000	0.0000	0.0000	0.0000	0.0370	0.0000	0.0019	0.0000	0.0000	0.0212	0.0000	0.0000	0.0000	0.0000	0.3744			
H11	0.0000	0.0000	0.0000	0.0000	0.0000	-0.0207	-0.0595	-0.0503	0.0000	0.0000	0.0000	0.0046	0.0000	0.0309	0.0231	0.0000	0.0000	0.0000	0.0000	0.1160	0.1801	0.0000	0.0000	0.0210	0.0860		
H12	0.0000	0.0000	0.0000	0.0000	0.0000	0.0000	0.0000	0.0421	-0.0088	0.0000	0.0000	0.0000	0.0000	0.0000	0.0000	0.0000	0.0000	0.0000	-0.0170	0.0000	-0.0853	0.0331	0.0000	0.0000	-0.0365	-0.0282	
H13	0.0000	0.0000	0.0000	0.0000	0.0000	0.0000	0.0000	0.0000	0.0909	0.0000	0.0000	0.0000	0.0000	0.0000	0.0000	-0.0057	0.0000	0.0000	0.0000	0.0000	0.0000	-0.1600	0.0000	0.0000	0.0000	0.0000	0.0000

(c) Cancer

	L1	L2	L3	L4	L5	L6	L7	L8	L9	L10	L11	L12	L13	L14	L18	H1	H2	H3	H4	H5	H7	H8	H9	H10	H11	H12
L1																										
L2	0.1247																									
L3	0.0000	0.0000																								
L4	0.0000	0.0000	0.1506																							
L5	0.0000	0.0374	0.0481	0.2536																						
L6	0.0000	0.0000	0.0000	0.0000	0.0000																					
L7	0.0000	0.0000	0.0000	0.0000	0.0000	0.2635																				
L8	0.0000	0.0000	0.0000	0.0540	0.0076	0.0000	0.0000																			
L9	0.0000	0.0297	0.0000	0.0000	0.0000	0.0000	0.0000	0.0000																		
L10	0.0000	0.0000	0.0000	0.0000	0.0000	0.0000	0.0000	0.0000	0.2102																	
L11	0.0000	0.0000	0.0000	0.0378	0.0182	0.0000	0.0000	0.0000	0.0000	0.0000																
L12	0.0000	0.0000	0.0000	0.0000	0.1601	0.0000	0.0000	0.0000	0.0000	0.0000	0.3367															
L13	0.0000	0.0000	0.0000	0.0000	0.0000	0.0000	0.0000	0.0000	0.0000	0.0000	0.0000	0.0000														
L14	0.0000	0.0000	0.0000	0.0000	0.0000	-0.0507	0.0000	0.0000	0.0000	0.0000	0.0000	0.0000	0.4208													
L18	0.0000	0.0000	0.0000	0.0000	0.0000	0.0000	0.0000	0.0000	0.0000	0.0000	0.0000	0.0000	0.0000	0.0000												
H1	0.0000	0.0000	0.0000	0.0562	0.0368	0.0000	0.0000	0.0000	0.0000	0.0000	0.0000	0.0000	0.0000	0.1460	0.0000											
H2	0.0000	0.0000	0.0000	-0.0372	0.0000	0.0000	0.0000	0.0000	0.0000	0.0000	0.0000	-0.0102	0.0000	0.0000	0.0000	-0.2339										
H3	0.0000	0.0000	0.0000	0.0000	0.0000	0.0000	0.0000	0.0000	0.0000	0.0000	0.0000	0.0000	0.0004	0.0000	0.0516	0.0000	0.0000									
H4	0.0000	0.0000	0.0000	0.0000	0.0000	0.0000	0.0000	0.0000	0.0000	0.0000	0.0000	0.0000	0.0000	0.0000	0.0495	0.0000	0.0000	0.0293								
H5	0.0000	0.0000	0.0000	0.0000	0.0000	0.0000	0.0000	0.0000	0.0000	0.0000	0.0000	0.0000	0.0998	0.0000	0.0000	0.0000	0.0000	0.1873	0.1565							
H7	0.0000	0.0000	0.0000	0.0000	0.0000	0.0000	0.0000	0.0000	0.0000	0.0000	0.0000	0.0000	0.0525	0.0000	0.0000	0.0000	0.0000	0.0000	0.0000	0.0000						

H8	0.0000	0.0000	0.0000	0.0000	0.0000	0.0000	0.0000	0.0000	0.0000	0.0000	0.0462	0.0128	0.0000	0.0000	0.0000	0.0405	0.0000	0.0000	0.0000	0.0000	0.1097					
H9	0.0000	0.0000	0.0000	0.0000	0.0000	0.0000	0.0000	0.0000	0.0000	0.0000	0.0000	0.0000	0.0000	0.0000	0.0000	0.0000	0.0000	0.0000	0.0000	0.0137	0.0000	0.0000				
H10	0.0000	0.0000	0.0000	0.0000	0.0000	0.0000	0.0000	0.0000	0.0000	0.0000	0.0000	0.0000	0.0000	0.0000	0.0000	0.0000	0.0000	0.0000	0.0000	0.0000	0.0000	0.0000	0.1884			
H11	0.0000	0.0000	0.0000	0.0000	0.0000	0.0000	0.0000	0.0000	0.0000	0.0000	0.0000	0.0000	0.0623	0.0000	0.0206	0.0000	0.0000	0.0000	0.0000	0.0687	0.0000	0.0000	0.0000	0.0000		
H12	0.0000	0.0000	0.0000	0.0000	0.0000	0.0000	0.0000	0.0000	0.0000	0.0000	0.0000	0.0000	0.0000	0.0000	0.0000	0.0000	0.0000	-0.0191	0.0000	0.0000	0.0000	0.0000	0.0000	0.0000	-0.0007	
H13	0.0000	0.0000	0.0000	0.0000	0.0000	0.0000	0.0000	0.0000	0.0000	0.0000	0.0000	0.0000	0.0000	0.0000	0.0000	0.0000	0.0000	0.0000	0.0000	0.0000	-0.0290	0.0000	0.0000	0.0000	0.0000	0.0000

(d) Chronic obstructive pulmonary disease

	L1	L2	L3	L5	L6	L7	L8	L9	L10	L11	L12	L13	L15	L18	H1	H2	H5	H6	H7	H8	H10	H11	H12
L1																							
L2	0.1441																						
L3	-0.0487	0.0000																					
L5	-0.0593	0.0000	0.1954																				
L6	0.0000	0.0000	0.0000	0.0000																			
L7	0.0000	0.0772	0.0000	0.0000	0.6320																		
L8	0.0000	0.0000	0.1015	0.0544	0.0000	0.0000																	
L9	0.0000	0.0000	0.0000	0.0000	0.0000	0.0000	0.0000																
L10	0.0000	0.0000	0.0000	0.0000	0.0000	0.0000	0.0000	0.2266															
L11	0.0000	0.0000	0.0622	0.0913	0.0000	0.0628	0.0093	0.0000	0.0000														
L12	0.0000	0.0000	0.0000	0.0647	0.0000	0.0000	0.0478	0.0000	0.0000	0.4973													
L13	0.0000	0.0000	0.0000	0.0000	-0.0414	-0.0633	0.0000	0.0000	0.0000	0.0000	0.0000												
L15	0.0000	0.0000	0.0000	0.0000	0.0000	0.0000	0.0000	0.0000	0.0000	0.0000	0.0000	0.0000											
L18	0.0000	0.0000	0.0000	0.0000	0.0000	0.0000	0.0000	0.0000	0.0000	0.0000	0.0000	0.0000	0.6840										
H1	0.0000	0.0000	0.0000	0.0000	0.0000	0.0000	0.0000	0.0000	0.0000	0.0107	0.0000	0.0040	0.0000	0.0000									
H2	0.1048	0.0000	0.0000	0.0000	0.0000	0.0000	0.0000	0.0000	0.0000	0.0000	0.0000	0.0000	0.0000	0.0000	-0.2506								
H5	0.0000	0.0704	0.0000	0.0000	0.0000	0.0000	0.0000	0.0000	0.0000	0.0000	0.0000	0.0000	0.0000	0.0528	0.0000	0.0000							
H6	0.0000	0.0000	0.0000	0.0000	0.0000	0.0000	0.0000	0.0000	0.0000	0.0000	0.0000	0.0000	0.0000	0.0000	0.0000	0.0000	0.0000						
H7	0.0000	0.0000	0.0000	0.0000	0.0000	0.0000	0.0000	0.0000	0.0000	0.0000	0.0000	0.0000	0.0000	0.0000	0.0000	0.0000	0.0000	0.4901					
H8	0.0000	0.0000	0.0000	0.0000	0.0000	0.0000	0.0000	0.0000	0.0000	0.0000	0.0000	0.0000	0.0000	0.0000	0.0000	0.0000	0.0000	0.0683	0.2336				

H10	0.0000	0.0000	0.0000	0.0000	0.0000	0.0000	0.0000	0.0000	0.0000	0.0000	0.0000	0.0000	0.0000	0.0000	0.0000	0.0000	0.0000	0.0000	0.0000	-0.0069			
H11	0.0000	0.0000	0.0000	0.0000	0.0000	0.0000	0.0000	0.0000	0.0000	0.0000	0.0000	0.0503	0.0000	0.0528	0.0000	0.0000	0.2027	0.0000	0.0000	0.0000	0.1454		
H12	0.0000	0.0000	0.0000	0.0000	0.0000	0.0000	0.0000	0.0000	0.0000	0.0000	0.0000	0.0000	0.0000	0.0000	0.0000	0.0000	-0.0603	0.0000	0.0000	0.0000	-0.0232	-0.0539	
H13	0.0000	0.0000	0.0000	0.0000	0.0000	0.0000	0.0000	0.0000	0.0000	0.0000	0.0000	0.0000	0.0000	0.0000	0.0000	0.0000	0.0000	-0.0720	0.0000	-0.0138	0.0302	0.0000	0.0000

(e) Depression

	L1	L2	L3	L5	L6	L7	L8	L9	L10	L11	L12	L13	L14	L15	L18	H1	H2	H3	H4	H5	H6	H7	H8	H9	H10	H11	H12
L1																											
L2	0.2755																										
L3	0.0000	0.0000																									
L5	-0.0318	0.0502	0.2106																								
L6	0.0122	0.0456	0.0000	0.0000																							
L7	0.0106	0.0736	0.0000	0.0000	0.6081																						
L8	0.0000	0.0000	0.1302	0.1634	0.0000	0.0000																					
L9	0.0076	0.0815	0.0000	0.0000	0.0223	0.0000	-0.0049																				
L10	0.0098	0.0000	0.0000	0.0000	0.0316	0.0094	-0.0435	0.3187																			
L11	0.0000	0.0596	0.0161	0.0829	0.0000	0.0000	0.0000	0.0000	0.0000																		
L12	0.0000	0.0000	0.0100	0.0825	0.0000	0.0000	0.1069	0.0000	0.0000	0.4930																	
L13	-0.0051	0.0000	0.0000	0.0000	0.0000	-0.0207	0.0000	-0.0153	0.0000	0.0164	0.0000																
L14	0.0000	0.0000	0.0152	0.0910	-0.1167	-0.0247	0.0000	0.0000	-0.0373	0.0053	0.0000	0.5746															
L15	0.0000	0.0539	0.0000	0.0102	0.0000	0.0000	0.0000	0.0000	0.0000	0.0000	0.0000	0.0235	0.0000														
L18	0.0000	0.0000	0.0000	0.0000	0.0000	0.0000	0.0000	0.0000	0.0000	-0.0176	0.0000	0.0162	0.0116	0.7401													
H1	-0.0202	0.0057	0.0125	0.0424	0.0000	0.0000	0.0532	0.0000	0.0000	0.0000	0.0384	0.0471	0.0430	0.0361	0.0054												
H2	0.1588	0.0000	0.0000	-0.0397	0.0000	0.0148	-0.0434	0.0000	0.0000	0.0000	-0.0408	0.0000	-0.0089	0.0000	0.0000	-0.4156											
H3	0.0058	0.0914	0.0000	0.0000	0.0000	0.0000	0.0000	0.0147	0.0316	0.0012	0.0000	0.0369	0.0000	0.1388	0.0708	0.0241	0.0000										
H4	0.0000	0.0000	0.0000	-0.0436	-0.0099	0.0000	0.0000	0.0000	-0.0204	0.0000	0.0000	0.0000	0.0000	0.0000	0.0141	0.0000	0.0384	0.1341									
H5	0.0000	0.0404	0.0054	0.0000	0.0000	0.0000	0.0000	0.0000	0.0000	0.0000	0.0000	0.0080	0.0000	0.0123	0.0077	0.0000	0.0000	0.2793	0.3277								
H6	0.0000	0.0000	0.0000	0.0000	0.0000	0.0000	0.0000	0.0000	0.0000	0.0000	0.0000	0.0141	0.0104	0.0000	-0.0101	0.0000	0.0000	0.0000	0.0000	0.0000							
H7	0.0000	0.0000	-0.0304	0.0000	0.0000	0.0000	0.0000	0.0000	0.0000	0.0000	0.0000	0.0088	0.0584	0.0000	0.0000	0.0000	-0.0135	-0.0167	0.0000	0.0000	0.6873						

H8	0.0000	0.0000	0.0000	0.0050	0.0000	0.0000	0.0000	0.0000	0.0000	0.0058	0.0000	0.0119	0.0000	0.0000	0.0000	0.0000	-0.0188	0.0000	0.0000	0.0000	0.0380	0.1803					
H9	0.0000	0.0026	0.0000	0.0000	0.0000	0.0000	0.0000	0.0334	0.0000	0.0000	0.0000	0.0000	0.0000	0.0000	0.0000	0.0000	0.0000	0.0000	0.0000	0.0607	-0.0285	0.0000	0.0000				
H10	0.0000	0.0000	-0.0069	-0.0008	0.0000	0.0000	-0.0057	0.0000	0.0000	0.0000	-0.0251	0.0000	0.0000	0.0000	0.0267	0.0000	0.0000	0.0000	0.0000	0.0409	0.0000	0.0000	0.0000	0.3360			
H11	0.0000	0.0000	0.0000	-0.0315	-0.0675	0.0000	0.0000	0.0000	0.0000	0.0000	0.0000	0.0439	0.0593	0.0487	0.0056	0.0000	0.0000	0.0000	0.0618	0.1527	0.0047	0.0414	0.0000	0.0797	0.1389		
H12	0.0000	0.0000	0.0000	0.0000	0.0000	0.0000	0.0774	0.0000	0.0000	0.0000	0.0000	0.0000	0.0000	0.0000	0.0000	0.0000	0.0000	-0.0463	-0.0273	-0.0805	0.0000	0.0000	0.0000	-0.0234	-0.0248	-0.1395	
H13	0.0000	0.0000	0.0000	0.0000	0.0000	0.0247	0.0000	0.0000	0.0000	-0.0263	0.0000	-0.0188	0.0000	0.0000	0.0293	0.0000	0.0000	0.0000	0.0000	0.0000	-0.0914	-0.0764	-0.0935	0.0000	0.0000	0.0000	0.0000

(f) Diabetes

	L1	L2	L3	L4	L5	L6	L7	L8	L9	L10	L11	L12	L13	L14	L16	L17	L18	H1	H2	H3	H4	H5	H7	H8	H9	H10	H11	H12
L1																												
L2	0.2656																											
L3	-0.0366	0.0000																										
L4	-0.0596	0.0271	0.1950																									
L5	-0.0628	0.0264	0.0611	0.4110																								
L6	0.0000	0.0287	-0.0594	0.0000	0.0000																							
L7	0.0000	0.0836	0.0000	0.0000	0.0262	0.6299																						
L8	-0.0209	0.0000	0.1415	0.1972	0.1022	0.0000	0.0000																					
L9	0.0789	0.0615	-0.0328	-0.0023	0.0000	0.0000	0.0000	-0.0788																				
L10	0.0000	0.1127	0.0000	0.0000	0.0000	0.0000	0.0594	0.0000	0.2282																			
L11	0.0000	0.0000	0.0105	0.0000	0.0639	0.0218	0.0000	0.0229	0.0000	-0.0018																		
L12	0.0000	0.0554	0.0000	0.0385	0.0980	0.0211	0.0000	0.0000	-0.0525	0.0000	0.5832																	
L13	0.0000	0.0000	0.0000	0.0140	0.0000	-0.0321	0.0000	0.0000	-0.0046	-0.0031	0.0000	0.0000																
L14	0.0000	0.0000	0.0608	0.0189	0.0000	-0.0680	-0.0587	0.0255	0.0000	-0.0497	0.0000	0.0266	0.5448															
L16	0.0000	0.0000	0.0000	0.0000	-0.0071	-0.0097	0.0000	0.0000	0.0000	0.0000	0.0000	-0.0484	0.0000	0.0000														
L17	0.0000	0.0000	0.0000	0.0000	-0.0134	0.0000	0.0000	0.0000	0.0000	0.0000	0.0000	-0.0420	0.0000	0.0000	0.3627													
L18	0.0000	0.0000	0.0051	0.0000	0.0000	0.0000	0.0000	0.0000	0.0000	0.0000	0.0000	0.0000	0.0000	0.0000	0.4491	0.4265												
H1	0.0000	0.0014	0.0000	0.0536	0.0194	0.0000	0.0000	0.0387	0.0000	0.0000	0.0665	0.0076	0.0346	0.0623	0.0000	0.0000	0.0245											
H2	0.0689	-0.0159	0.0000	-0.0820	0.0000	0.0000	0.0000	-0.0060	0.0000	0.0444	0.0000	-0.0108	0.0000	-0.0079	0.0000	0.0000	0.0000	-0.4534										
H3	0.0000	0.1112	0.0000	0.0000	0.0000	0.0000	0.0000	0.0000	0.0290	0.0175	0.0000	0.0000	0.0506	0.0000	0.0000	0.0327	0.0663	0.0000	0.0382									

H4	0.0216	0.0000	0.0000	0.0000	0.0000	0.0000	0.0000	0.0000	0.0000	0.0000	0.0000	-0.0121	0.0000	0.0000	0.0511	0.0000	0.0392	0.0000	0.0900	0.1543								
H5	0.0000	0.1010	0.0000	0.0000	0.0000	0.0000	0.0000	0.0000	0.0000	0.0000	0.0000	0.0000	0.0134	0.0427	0.0000	0.0000	0.0576	0.0541	0.0000	0.2261	0.3137							
H7	0.0000	0.0000	0.0000	0.0000	0.0000	0.0000	-0.0251	0.0000	0.0000	0.0000	0.0000	0.0000	0.0339	0.0572	0.0000	0.0000	0.0000	0.0000	0.0000	0.0000	0.0000	0.0000						
H8	0.0000	0.0423	0.0293	0.0281	0.0697	0.0000	0.0000	0.0088	0.0000	0.0000	0.0145	0.1068	0.0000	0.0266	0.0000	0.0000	0.0000	0.0000	-0.0508	0.0000	0.0000	0.0215	0.3285					
H9	0.0000	0.1538	0.0000	0.0000	0.0420	0.0140	0.0000	0.0000	0.0690	0.0000	0.0000	0.0000	0.0000	0.0000	0.0000	0.0000	0.0000	0.0230	0.0000	0.0000	0.0000	0.0543	0.0000	0.0000				
H10	0.0000	0.0000	0.0000	0.0000	0.0000	0.0000	0.0000	-0.0259	0.0000	0.0839	-0.0447	0.0000	0.0000	0.0000	0.0000	0.0000	0.0000	0.0000	0.0000	0.0000	0.0013	0.0383	0.0000	0.0000	0.2620			
H11	0.0000	0.0000	0.0000	-0.0539	-0.0488	-0.0811	-0.0484	0.0000	0.0000	0.0000	0.0000	0.0000	0.0410	0.0000	0.0000	0.0469	0.0000	0.0000	0.0000	0.0000	0.0260	0.0892	0.0356	0.0000	0.0185	0.0799		
H12	-0.0162	-0.0234	0.0000	0.0000	0.0000	0.0000	0.0000	0.0222	0.0000	-0.0154	0.0000	0.0000	0.0000	0.0000	0.0000	0.0000	0.0000	0.0000	0.0000	-0.0888	0.0000	-0.1278	-0.0148	-0.0401	-0.0035	-0.0815	-0.1432	
H13	0.0000	0.0000	0.0000	0.0000	0.0000	0.0000	0.0183	0.0000	0.0713	0.0000	0.0000	0.0000	0.0000	-0.0350	0.0000	0.0000	0.0000	0.0000	0.0260	0.0000	0.0000	0.0000	-0.2161	-0.0726	0.0433	0.0000	0.0000	0.0101

(g) Eczema

	L1	L2	L3	L4	L5	L6	L7	L8	L9	L10	L11	L12	L13	L14	L15	L16	L17	L18	H1	H2	H3	H4	H5	H6	H7	H8	H9	H10	H11	H12
L1																														
L2	0.3520																													
L3	0.0000	0.0000																												
L4	-0.0171	0.0296	0.1686																											
L5	-0.0068	0.0000	0.1662	0.3720																										
L6	0.0000	0.0320	-0.0638	0.0000	0.0000																									
L7	0.0000	0.0401	0.0000	0.0000	0.0123	0.6522																								
L8	0.0000	0.0320	0.1537	0.0100	0.1596	0.0000	0.0084																							
L9	0.0000	0.0713	-0.0214	0.0000	0.0000	0.0000	0.0000	0.0000																						
L10	0.0199	0.0541	-0.1097	-0.0280	0.0000	0.0000	0.0469	0.0000	0.3683																					
L11	0.0000	0.0000	0.0000	0.0000	0.0520	0.0000	0.0000	0.0000	0.0000	0.0000																				
L12	-0.0216	0.0000	0.0000	0.0418	0.0320	0.0000	0.0124	0.0177	-0.0580	0.0000	0.4695																			
L13	0.0000	0.0000	0.0220	0.0259	0.0000	0.0000	-0.0185	0.0000	0.0000	0.0000	0.0000	0.0045																		
L14	0.0000	0.0000	0.0000	0.0844	0.0292	-0.0797	-0.0061	0.0000	0.0000	0.0000	0.0000	0.0000	0.5493																	
L15	0.0221	0.0000	0.0000	0.0000	0.0000	0.0000	0.0000	0.0000	0.0000	0.0000	0.0000	0.0000	0.0000	0.0000																
L16	0.0000	0.0000	0.0000	0.0000	0.0064	0.0000	0.0000	0.0109	0.0000	0.0000	0.0000	0.0000	0.0000	0.0000	0.3444															
L17	0.0000	0.0126	0.0000	0.0000	0.0000	0.0000	0.0000	0.0000	0.0000	0.0000	0.0000	-0.0079	0.0000	0.0028	0.0940	0.3386														

L18	0.0000	0.0072	0.0111	0.0000	0.0000	0.0000	0.0000	0.0000	0.0000	0.0000	0.0000	0.0000	0.0226	0.0074	0.3624	0.2927	0.3372													
H1	-0.0497	0.0000	0.0000	0.0000	0.0000	0.0000	0.0000	0.0707	0.0000	0.0000	0.0000	0.0000	0.0593	0.0284	0.0168	0.0000	0.0000	0.0430												
H2	0.0000	0.0000	0.0000	-0.0915	0.0000	0.0000	0.0000	-0.0149	0.0000	0.0000	0.0000	0.0000	-0.0048	-0.0206	0.0000	0.0000	0.0000	0.0000	-0.3870											
H3	0.0423	0.0710	0.0000	0.0000	0.0000	0.0000	0.0000	0.0000	0.0000	0.0000	0.0000	0.0000	0.0000	0.0000	0.0000	0.0699	0.0196	0.0556	0.0000	0.0123										
H4	0.0000	0.0000	0.0000	0.0000	0.0000	0.0000	0.0000	0.0000	0.0000	0.0000	0.0000	0.0000	0.0000	0.0000	0.0156	0.0000	0.0000	0.0000	0.0000	0.0172	0.2127									
H5	0.0000	0.0091	0.0163	0.0671	0.0000	0.0000	0.0000	0.0000	0.0000	0.0000	0.0000	0.0000	0.0000	0.0000	0.0274	0.0291	0.0000	0.0000	0.0000	0.0000	0.1567	0.2856								
H6	0.0000	0.0000	0.0000	0.0000	0.0000	0.0000	0.0000	-0.0249	0.0000	0.0000	0.0000	0.0000	0.0415	0.0000	0.0000	0.0000	0.0000	0.0000	0.0000	0.0000	0.0000	0.0000	0.0000							
H7	0.0000	0.0000	0.0000	0.0214	0.0000	0.0000	0.0000	0.0000	0.0000	0.0000	0.0000	0.0420	0.0297	0.0193	0.0000	0.0000	-0.0098	0.0000	0.0000	0.0000	0.0000	0.0000	0.0000	0.6755						
H8	0.0000	0.0000	0.0000	0.0204	0.0000	0.0000	0.0000	0.0000	0.0000	0.0000	0.0751	0.0000	0.0187	0.0000	0.0000	0.0000	0.0000	0.0000	0.0344	0.0000	0.0026	0.0000	0.0000	0.0310	0.1657					
H9	0.0000	0.0204	0.0000	0.0000	0.0000	0.0316	0.0000	0.0076	0.0000	0.0000	0.0000	0.0000	0.0000	0.0000	0.0000	0.0000	0.0000	0.0000	0.0000	0.0018	0.0000	0.0000	0.0630	0.0000	0.0000	0.0000				
H10	0.0000	0.0138	0.0000	0.0000	0.0000	0.0000	0.0000	-0.0730	0.0162	0.0167	0.0000	0.0000	0.0000	0.0000	0.0000	0.0000	0.0000	0.0661	0.0000	0.0000	0.0000	0.0000	0.0000	0.0000	0.0000	0.0000	0.3080			
H11	0.0000	0.0000	0.0000	0.0000	0.0000	-0.0148	-0.0371	0.0000	-0.0367	-0.0169	0.0000	0.0000	0.0952	0.0691	0.0099	0.0000	0.0000	0.0389	0.0000	0.0000	0.0054	0.0232	0.1843	0.0270	0.0000	0.0000	0.0325	0.0918		
H12	0.0000	0.0000	0.0000	0.0000	0.0000	0.0000	0.0000	0.0008	0.0000	0.0000	0.0000	0.0160	0.0000	-0.0449	0.0000	0.0000	0.0000	0.0000	-0.0219	0.0000	-0.0362	0.0000	-0.0823	0.0000	0.0000	-0.0132	0.0000	-0.0823	-0.0863	
H13	0.0000	0.0000	0.0217	0.0000	0.0000	0.0000	0.0000	0.0000	0.0000	0.0000	0.0000	0.0000	0.0000	0.0000	0.0000	0.0000	0.0000	0.0000	0.0000	0.0000	0.0000	0.0000	0.0000	-0.0887	-0.0967	-0.0494	0.0000	0.0000	0.0000	0.0000

(h) Liver

	L1	L2	L3	L4	L5	L6	L8	L10	L11	L12	L13	L15	L16	L18	H1	H2	H3	H4	H5	H6	H7	H8	H9	H10	H11	H12
L1																										
L2	0.2365																									
L3	0.0000	0.0000																								
L4	-0.0916	0.0000	0.2490																							
L5	0.0000	0.0326	0.1015	0.3884																						
L6	0.0000	0.1760	0.0000	0.0000	0.0000																					
L8	0.0000	0.0272	0.2224	0.0303	0.1499	0.0000																				
L10	0.0000	0.0844	-0.0029	0.0000	-0.0081	0.1939	-0.0848																			
L11	0.0000	0.0000	0.0000	0.0259	0.0412	0.0000	0.0000	0.0000																		
L12	0.0000	0.0000	0.0490	0.0581	0.0772	0.0000	0.0000	-0.0214	0.4817																	
L13	0.0000	0.0000	0.0079	0.0612	0.0000	-0.1862	0.0000	-0.0350	0.0000	0.0000																
L15	0.0000	0.0000	0.0000	0.0000	0.0000	0.0000	0.0000	0.0000	0.0000	0.0000	0.0000															

L16	0.0000	0.0000	0.0000	0.0000	0.0000	0.0000	-0.0103	0.0000	0.0000	0.0000	0.0000	0.5453														
L18	0.0000	0.0000	0.0000	0.0000	0.0000	0.0000	0.0000	0.0000	0.0000	-0.0039	0.0583	0.3880	0.3973													
H1	-0.0456	0.0352	0.0000	0.0000	0.0626	0.0000	0.0161	0.0000	0.0000	0.0264	0.0986	0.0000	0.0000	0.0267												
H2	0.2056	0.0000	-0.0310	-0.0712	0.0000	0.0351	-0.0494	0.0000	0.0000	-0.0551	-0.0313	0.0000	0.0000	0.0000	-0.3545											
H3	0.0000	0.0599	0.0000	0.0000	0.0000	0.0000	0.0000	0.0000	0.0000	0.0000	0.0000	0.0019	0.0000	0.1547	0.0000	0.0000										
H4	0.0000	0.0161	0.0000	0.0000	0.0000	0.0000	0.0000	0.0000	0.0000	0.0000	0.0282	0.0000	0.0062	0.0135	0.0000	0.0000	0.1803									
H5	0.0000	0.0766	0.0000	0.0000	0.0000	0.0000	0.0000	0.0000	0.0000	0.0000	0.0000	0.0000	0.0204	0.0000	0.0000	0.0000	0.2315	0.3168								
H6	0.0000	0.0000	0.0000	0.0000	0.0000	0.0000	0.0000	0.0000	0.0000	0.0000	0.0288	0.0047	0.0000	0.0000	0.0000	-0.0723	0.0000	0.0000	-0.0223							
H7	0.0000	0.0000	0.0000	0.0000	0.0000	-0.0436	0.0000	-0.0274	0.0000	0.0173	0.0847	0.0000	0.0000	0.0000	0.0000	-0.0054	0.0000	0.0000	0.0000	0.6844						
H8	0.0000	0.0000	0.0000	0.0552	0.0113	0.0000	0.0162	-0.0208	0.0716	0.0208	0.0000	0.0000	0.0000	0.0000	0.0000	-0.0114	0.0000	0.0000	0.0000	0.0187	0.1724					
H9	0.0000	0.0191	0.0000	0.0000	0.0000	0.0000	0.0000	0.0000	0.0000	0.0000	0.0000	0.0000	0.0000	0.0000	0.0000	0.0000	0.0000	0.0000	0.0000	-0.0380	-0.0210	-0.0241				
H10	0.0000	0.0000	0.0000	0.0000	0.0000	0.0000	-0.0534	0.0224	-0.0159	-0.0155	0.0000	0.0037	0.0000	0.0000	0.0000	0.0000	0.0018	0.0620	0.0386	0.0000	0.0000	0.0000	0.3595			
H11	0.0000	0.0000	0.0000	0.0000	0.0000	-0.0116	0.0000	0.0000	0.0000	0.0000	0.0994	0.0192	0.0332	0.0000	0.0000	0.0000	0.0038	0.0233	0.1690	0.0000	0.0509	0.0000	0.0045	0.1070		
H12	-0.0132	0.0000	0.0000	0.0000	0.0000	0.0000	0.0000	0.0000	0.0000	0.0000	0.0000	0.0000	0.0000	0.0000	0.0000	-0.0125	-0.0318	0.0000	-0.0804	0.0000	0.0000	0.0000	-0.0223	-0.0505	-0.1006	
H13	0.0000	0.0136	0.0000	0.0000	0.0000	0.0000	0.0000	0.0000	0.0000	0.0000	0.0000	-0.0015	0.0000	0.0000	0.0000	0.0000	0.0000	0.0000	0.0085	-0.1543	-0.0809	0.0000	0.0086	0.0000	0.0000	-0.1250

(i) Gastric ulcer

	L1	L2	L3	L4	L5	L6	L7	L8	L9	L10	L11	L12	L13	L18	H1	H2	H3	H4	H5	H6	H7	H8	H9	H10	H11	H12
L1																										
L2	0.2686																									
L3	0.0000	0.0000																								
L4	-0.0130	0.0000	0.1396																							
L5	-0.0122	0.0000	0.1650	0.3286																						
L6	0.0000	0.0000	0.0000	0.0000	0.0000																					
L7	0.0000	0.0646	-0.0310	0.0000	0.0000	0.6507																				
L8	0.0000	0.0000	0.1491	0.1082	0.1116	0.0000	0.0000																			
L9	0.0000	0.0000	-0.0685	0.0000	0.0000	0.0066	0.0000	0.0000																		
L10	0.0291	0.0000	-0.1243	-0.0450	0.0000	0.0606	0.0000	-0.0747	0.2536																	
L11	0.0000	0.0436	0.0000	0.0000	0.0840	0.0156	0.0000	0.0000	0.0000	0.0000																

L12	-0.0242	0.0000	0.0000	0.0258	0.0798	0.0000	0.0000	0.0000	0.0000	0.0000	0.5705															
L13	0.0000	0.0000	0.0330	0.0343	0.0188	-0.1239	-0.0221	0.0000	0.0000	-0.0363	0.0000	0.0000														
L18	0.0000	0.0557	0.0000	0.0000	0.0000	0.0000	0.0000	0.0000	0.0000	0.0000	0.0000	-0.0029	0.0000													
H1	0.0000	0.0000	0.0000	0.0000	0.0282	0.0000	-0.0044	0.0000	0.0000	0.0000	0.0000	0.0000	0.0507	0.0000												
H2	0.1050	0.0000	0.0000	-0.1027	0.0000	0.0046	0.0000	-0.0412	0.0000	0.0000	0.0000	0.0000	-0.0392	0.0000	-0.3474											
H3	0.0131	0.0273	0.0000	0.0000	0.0000	0.0000	0.0000	0.0000	0.0000	0.0000	0.0000	0.0000	0.0000	0.1373	-0.0145	0.1161										
H4	0.0097	0.0318	0.0000	0.0000	0.0000	0.0000	0.0000	0.0000	0.0000	0.0000	0.0000	0.0000	0.0000	0.0001	0.0000	0.0000	0.1893									
H5	0.0000	0.0938	0.0266	0.0000	0.0000	0.0000	0.0000	0.0000	0.0000	0.0000	0.0000	0.0000	0.0000	0.1092	0.0000	0.0000	0.2211	0.2573								
H6	0.0000	0.0000	0.0000	0.0000	0.0000	-0.0006	0.0000	-0.0293	0.0000	0.0000	0.0000	0.0000	0.0528	0.0000	0.0095	0.0000	0.0000	0.0000	-0.0439							
H7	0.0000	0.0000	0.0000	0.0000	0.0000	0.0000	0.0000	0.0000	0.0000	0.0000	0.0000	0.0000	0.0000	0.0000	0.0000	0.0000	-0.0050	0.0000	0.0000	0.6307						
H8	0.0000	0.0000	0.0000	0.0000	0.0030	0.0000	0.0000	0.0000	0.0000	0.0000	0.0000	0.0058	0.0465	0.0000	0.0440	-0.0270	0.0000	0.0000	0.0000	0.0235	0.2348					
H9	0.0100	0.0564	0.0000	0.0000	0.0000	0.0000	0.0000	0.0000	0.0000	0.0000	0.0000	0.0000	0.0000	0.0000	0.0000	0.0000	0.0849	0.0000	0.0133	-0.0400	0.0000	-0.0530				
H10	0.0000	0.0000	0.0000	0.0000	0.0000	0.0000	0.0000	0.0000	0.0000	0.0928	-0.0420	-0.0178	0.0000	0.0278	0.0000	0.0000	0.0000	0.0000	0.0000	0.0000	0.0000	0.0000	0.3457			
H11	0.0000	0.0396	0.0000	0.0000	0.0000	0.0000	0.0000	0.0000	0.0000	0.0000	0.0000	0.0000	0.0445	0.0894	0.0000	0.0000	0.0278	0.0246	0.1197	0.0000	0.0000	0.0000	0.0213	0.0896		
H12	0.0000	0.0000	0.0000	0.0000	0.0000	0.0000	0.0000	0.0000	0.0000	0.0000	0.0000	0.0000	-0.0145	-0.0468	0.0000	0.0000	0.0000	0.0000	-0.1339	0.0000	0.0000	0.0000	-0.0378	-0.0063	-0.1207	
H13	0.0000	0.0000	0.0000	0.0000	0.0000	0.0000	0.0000	0.0000	0.0000	0.0000	0.0000	0.0000	0.0000	0.0000	0.0000	0.0000	0.0000	0.0000	0.0422	-0.1074	-0.0909	-0.0734	0.0387	0.0000	0.0000	-0.0514

(j) Hearing problems

	L1	L2	L3	L5	L6	L8	L9	L10	L11	L12	L13	L14	L16	L17	L18	H1	H2	H3	H4	H5	H7	H8	H9	H10	H11	H12
L1																										
L2	0.1588																									
L3	0.0000	0.0000																								
L5	0.0000	0.0000	0.0000																							
L6	0.0000	0.0767	0.0000	0.0000																						
L8	0.0000	0.0000	0.0335	0.1305	0.0000																					
L9	0.0000	0.0000	0.0000	0.0000	0.0000	0.0000																				
L10	0.0000	0.0000	0.0000	0.0000	0.0000	0.0000	0.1647																			
L11	0.0000	0.0000	0.0000	0.0414	0.0000	0.0000	0.0000	0.0000																		
L12	0.0000	0.0000	0.0000	0.0724	0.0000	0.0271	0.0000	0.0000	0.3723																	

L13	0.0000	0.0000	0.0000	0.0000	0.0000	0.0581	0.0000	0.0000	0.0000	0.0000																
L14	0.0000	0.0000	0.0000	0.0000	-0.1374	0.0358	0.0000	0.0000	0.0000	0.0000	0.4181															
L16	0.0000	0.0000	0.0000	0.0000	0.0000	0.0000	0.0000	0.0000	0.0000	-0.0331	0.0000	0.0000														
L17	0.0000	0.0000	0.0000	0.0000	0.0000	0.0000	0.0000	0.0000	0.0000	0.0000	0.0000	0.0000	0.3369													
L18	0.0000	0.0000	0.0000	0.0000	0.0000	0.0000	0.0000	0.0000	0.0000	0.0000	0.0000	0.0000	0.4151	0.3353												
H1	0.0000	0.0000	0.0000	0.0000	0.0000	0.0000	0.0000	0.0000	0.0000	0.0000	0.0000	0.0146	0.0000	0.0000	0.0000											
H2	0.0000	0.0000	0.0000	0.0000	0.0000	0.0000	0.0000	0.0000	0.0000	0.0000	-0.0260	0.0000	0.0000	0.0000	0.0000	-0.2484										
H3	0.0000	0.0872	0.0000	0.0000	0.0000	0.0000	0.0000	0.0000	0.0000	0.0000	0.0000	0.0000	0.0220	0.0000	0.0867	0.0000	0.0000									
H4	0.0000	0.0000	0.0000	0.0000	0.0000	0.0000	0.0000	0.0000	0.0000	0.0000	0.0000	0.0000	0.0000	0.0000	0.0000	0.0000	0.0100	0.0606								
H5	0.0000	0.0000	0.0000	0.0000	0.0000	0.0000	0.0000	0.0000	0.0000	0.0000	0.0000	0.0000	0.0253	0.0000	0.0073	0.0000	0.0000	0.0591	0.1881							
H7	0.0000	0.0000	0.0000	0.0000	0.0000	0.0000	0.0000	0.0000	0.0000	0.0000	0.0847	0.0000	0.0000	0.0000	0.0000	0.0000	0.0000	0.0000	0.0000	0.0000						
H8	0.0000	0.0000	0.0000	0.0000	0.0000	0.0000	0.0000	0.0000	0.0000	0.0027	0.0000	0.0000	0.0000	0.0000	0.0000	0.0000	0.0000	0.0000	0.0000	0.0000	0.1548					
H9	0.0000	0.0032	0.0000	0.0000	0.0000	0.0000	0.0000	0.0000	0.0000	0.0000	0.0000	0.0000	0.0000	0.0000	0.0000	0.0000	0.0000	0.0686	0.0000	0.0641	0.0000	0.0000				
H10	0.0000	0.0000	0.0000	0.0000	0.0000	0.0000	0.0000	0.0000	0.0000	0.0000	0.0000	0.0000	0.0000	0.0000	0.0000	0.0000	0.0000	0.0000	0.0000	0.0000	0.0000	0.0000	0.1389			
H11	0.0000	0.0000	0.0000	0.0000	0.0000	0.0000	0.0000	0.0000	0.0000	0.0000	0.0000	0.0000	0.0000	0.0000	0.0000	0.0000	0.0000	0.0000	0.0602	0.0346	0.0000	0.0000	0.0000	0.0943		
H12	0.0000	0.0000	0.0000	0.0000	0.0000	0.0000	0.0000	0.0000	0.0000	0.0000	0.0000	0.0000	0.0000	0.0000	0.0000	0.0000	0.0000	0.0000	0.0000	0.0000	0.0000	0.0000	-0.0193	-0.0497	0.0000	
H13	0.0000	0.0000	0.0000	0.0000	0.0000	0.0000	0.0355	0.0000	0.0000	0.0000	0.0000	0.0000	0.0000	0.0000	0.0000	0.0000	0.0000	0.0000	0.0000	0.0000	-0.0228	-0.0237	0.0000	0.0000	0.0000	0.0000

(k) Heart attack

	L1	L2	L3	L4	L5	L6	L7	L8	L10	L11	L12	L13	L14	L15	L16	L18	H1	H2	H3	H4	H5	H6	H7	H8	H9	H10	H11	H12
L1																												
L2	0.3117																											
L3	0.0000	0.0000																										
L4	0.0000	0.0278	0.1508																									
L5	0.0000	0.0000	0.1271	0.3886																								
L6	0.0000	0.0205	0.0000	0.0000	0.0000																							
L7	0.0000	0.1474	0.0000	0.0000	0.0124	0.5374																						
L8	0.0000	0.0000	0.1257	0.0000	0.1393	0.0000	0.0472																					
L10	0.0067	0.0869	0.0000	0.0000	0.0000	0.0068	0.0000	0.0000																				

L11	0.0000	0.0000	0.0332	0.0000	0.1318	0.0000	0.0000	0.0176	-0.0242																			
L12	0.0000	0.0000	0.0332	0.1195	0.0490	0.0000	0.0000	0.0000	-0.0304	0.4280																		
L13	0.0000	0.0000	0.0000	0.0000	0.0117	-0.0030	0.0000	0.0000	-0.0148	0.0000	0.0000																	
L14	0.0000	0.0000	0.0000	0.0200	0.0000	-0.1631	0.0000	0.0000	-0.0201	0.0000	0.0000	0.6087																
L15	0.0000	0.0000	0.0000	0.0000	0.0000	0.0000	0.0000	0.0000	0.0000	0.0000	0.0000	0.0000	0.0000															
L16	0.0000	0.0000	0.0000	0.0000	0.0000	0.0000	0.0000	0.0000	0.0000	0.0000	0.0000	0.0051	0.0000	0.4563														
L18	0.0000	0.0559	0.0000	0.0000	0.0000	0.0000	0.0000	0.0000	0.0000	0.0000	0.0000	0.0245	0.0000	0.3733	0.4741													
H1	-0.0160	0.0000	0.0027	0.0518	0.0304	0.0000	0.0000	0.0476	0.0000	0.0000	0.0164	0.0408	0.0369	0.0292	0.0000	0.0000												
H2	0.1415	0.0000	-0.0052	-0.0988	0.0000	0.0000	0.0000	-0.0087	0.0000	0.0000	-0.0252	-0.0605	-0.0202	0.0000	0.0000	0.0000	-0.3199											
H3	0.0039	0.0238	0.0000	0.0000	0.0000	0.0000	0.0000	0.0000	0.0000	0.0000	0.0000	0.0000	0.0000	0.0209	0.0383	0.0514	0.0000	0.0000										
H4	0.0000	0.0000	0.0000	0.0000	0.0000	0.0000	0.0000	0.0000	-0.0148	0.0000	0.0000	0.0000	0.0000	0.0049	0.0137	0.0000	0.0000	0.0791	0.2007									
H5	0.0000	0.0000	0.0000	0.0000	0.0000	0.0000	0.0000	0.0000	0.0000	0.0108	0.0000	0.0328	0.0000	0.0459	0.0000	0.0374	0.0000	0.0000	0.2468	0.2849								
H6	-0.0080	0.0000	0.0000	0.0000	0.0000	0.0000	0.0000	0.0000	0.0000	0.0000	0.0000	0.0413	0.0000	0.0000	0.0000	0.0000	0.0000	0.0000	0.0000	0.0000	0.0000							
H7	0.0000	0.0000	0.0000	0.0000	0.0000	0.0000	0.0000	0.0000	0.0000	0.0037	0.0000	0.0000	0.0236	0.0000	0.0000	0.0000	0.0000	-0.0129	0.0000	0.0000	0.0000	0.6371						
H8	0.0000	0.0000	0.0000	0.0422	0.0000	0.0000	0.0000	0.0000	0.0000	0.0000	0.0000	0.0782	0.0208	0.0000	0.0000	0.0000	0.0000	-0.0126	0.0000	0.0000	0.0262	0.0000	0.2405					
H9	0.0000	0.0000	0.0000	0.0000	0.0000	0.0000	0.0000	0.0000	0.0000	0.0000	0.0000	0.0000	0.0000	0.0000	0.0000	0.0000	0.0000	0.0000	0.0000	0.0000	0.0193	-0.0139	0.0000	0.0000				
H10	0.0000	0.0000	0.0000	0.0000	0.0000	0.0000	0.0000	0.0000	0.0130	0.0000	0.0000	0.0000	0.0000	0.0353	0.0000	0.0012	0.0000	0.0000	0.0169	0.0000	0.0000	0.0000	0.0000	0.0000	0.3635			
H11	0.0000	0.0000	0.0000	0.0000	0.0000	-0.0154	-0.0519	-0.0407	0.0000	0.0000	0.0000	0.0019	0.0000	0.0299	0.0225	0.0000	0.0000	0.0000	0.0000	0.1140	0.1780	0.0000	0.0000	0.0000	0.0163	0.0802		
H12	0.0000	0.0000	0.0000	0.0000	0.0000	0.0000	0.0000	0.0311	0.0000	0.0000	0.0000	0.0000	0.0000	0.0000	0.0000	0.0000	0.0000	0.0000	-0.0115	0.0000	-0.0796	0.0176	0.0043	0.0000	0.0000	-0.0284	-0.0219	
H13	0.0000	0.0000	0.0000	0.0000	0.0000	0.0000	0.0000	0.0000	0.0782	0.0000	0.0000	0.0000	0.0000	0.0000	0.0000	0.0000	0.0000	0.0000	0.0000	0.0000	0.0000	-0.1422	-0.0231	0.0000	0.0000	0.0000	0.0000	0.0000

(I) High cholesterol

	L1	L2	L3	L4	L5	L6	L8	L9	L10	L11	L12	L13	L14	L15	L17	L18	H1	H2	H3	H4	H5	H6	H7	H8	H9	H10	H11	H12
L1																												
L2	0.2757																											
L3	0.0000	0.0000																										
L4	-0.0723	0.0304	0.1899																									
L5	-0.0042	0.0386	0.0994	0.4341																								
L6	0.0000	0.1779	-0.0749	0.0000	0.0253																							

L8	0.0000	0.0357	0.1545	0.0943	0.1787	0.0000																						
L9	0.0373	0.1091	0.0000	-0.0043	0.0000	0.0393	-0.0591																					
L10	0.0000	0.0365	0.0000	0.0000	0.0000	0.0209	-0.0494	0.3409																				
L11	0.0000	0.0000	0.0000	0.0493	0.0771	0.0248	0.0047	-0.0410	0.0000																			
L12	0.0000	0.0000	0.0000	0.0000	0.1032	0.0289	0.0000	-0.0482	0.0000	0.4902																		
L13	0.0000	0.0000	0.0000	0.0000	0.0000	-0.0823	0.0000	0.0000	-0.0032	0.0000	0.0212																	
L14	0.0000	0.0000	0.0512	0.0411	0.0113	-0.1188	0.0160	0.0000	-0.0679	0.0290	0.0000	0.5741																
L15	0.0000	0.0000	0.0000	0.0000	0.0000	0.0000	0.0000	0.0000	0.0000	0.0000	0.0000	0.0000	0.0000															
L17	0.0000	0.0000	0.0000	-0.0055	-0.0149	-0.0292	0.0000	0.0000	0.0000	-0.0018	-0.0192	0.0288	0.0000	0.3084														
L18	0.0000	0.0000	0.0000	0.0000	0.0000	0.0000	0.0000	0.0000	0.0163	0.0000	0.0000	0.0265	0.0000	0.5521	0.4011													
H1	-0.0654	0.0156	0.0000	0.0000	0.0451	-0.0182	0.0041	-0.0016	0.0000	0.0000	0.0000	0.0518	0.0570	0.0000	0.0000	0.0414												
H2	0.0728	0.0000	0.0000	-0.0560	0.0000	0.0120	0.0000	0.0000	0.0173	0.0000	-0.0211	-0.0465	-0.0281	0.0000	0.0000	0.0000	-0.3363											
H3	0.0000	0.0865	0.0000	0.0000	0.0000	0.0000	0.0000	0.0000	0.0000	0.0000	0.0000	0.0197	0.0227	0.0000	0.0208	0.0865	0.0000	0.0663										
H4	0.0205	0.0000	0.0000	0.0000	0.0000	0.0000	0.0000	0.0000	0.0000	-0.0063	0.0000	0.0000	0.0189	0.0585	0.0085	0.0000	0.0000	0.0089	0.2016									
H5	0.0228	0.0468	0.0237	0.0000	0.0230	0.0000	0.0197	0.0000	0.0000	0.0000	0.0000	0.0000	0.0016	0.0000	0.0372	0.0240	0.0000	0.0000	0.2044	0.3605								
H6	0.0000	0.0000	0.0000	0.0000	-0.0036	0.0000	0.0000	0.0000	0.0000	0.0000	0.0000	0.0141	0.0165	0.0000	0.0000	0.0000	0.0284	-0.0026	0.0000	0.0000	-0.0213							
H7	-0.0275	0.0000	0.0000	0.0000	0.0000	-0.0526	0.0000	0.0000	0.0000	0.0000	0.0443	0.0332	0.0000	0.0000	0.0000	0.0000	0.0083	0.0000	0.0000	0.0000	0.0000	0.6520						
H8	0.0000	0.0208	0.0000	0.0607	0.0104	0.0000	0.0000	0.0000	0.0000	0.0373	0.0718	0.0306	0.0000	0.0000	-0.0519	0.0000	0.0484	-0.0691	0.0000	0.0000	0.0155	0.0269	0.1877					
H9	0.0000	0.1082	0.0000	0.0000	0.0317	0.0000	0.0000	0.0329	0.0000	0.0000	0.0000	0.0000	0.0000	0.0000	0.0000	0.0000	0.0000	0.0152	0.0127	0.0000	0.0402	-0.0464	0.0000	0.0000				
H10	0.0000	0.0000	-0.0515	-0.0084	0.0000	0.0000	-0.0667	0.0418	0.0000	-0.0070	0.0000	0.0000	0.0000	0.0064	0.0000	0.0165	0.0000	0.0000	0.0161	0.0000	0.0000	0.0000	-0.0091	-0.0418	0.3514			
H11	0.0000	0.0000	0.0000	-0.0109	-0.0540	-0.0679	0.0000	0.0000	-0.0154	0.0000	0.0000	0.0184	0.1047	0.0000	0.0653	0.0000	0.0000	0.0000	0.0000	0.0639	0.1369	0.0113	0.0429	0.0000	0.0255	0.0618		
H12	0.0000	-0.0335	0.0000	0.0000	0.0000	0.0000	0.0600	0.0000	0.0000	-0.0187	0.0000	0.0000	0.0000	0.0000	0.0000	0.0000	0.0000	-0.0051	-0.0416	-0.0041	-0.0797	0.0000	0.0000	-0.0372	-0.0602	-0.0380	-0.1175	
H13	0.0000	0.0000	0.0000	0.0000	0.0000	0.0000	0.0000	0.0000	0.0042	0.0000	0.0000	-0.0184	0.0000	0.0054	0.0000	0.0000	-0.0018	0.0225	0.0000	0.0000	0.0065	-0.1459	-0.0552	-0.0598	0.0385	0.0000	0.0000	0.0000

(m) Hypertension

	L1	L2	L3	L4	L5	L6	L7	L8	L9	L10	L11	L12	L13	L14	L15	L18	H1	H2	H3	H4	H5	H6	H7	H8	H9	H10	H11	H12
L1																												
L2	0.2776																											
L3	-0.0235	0.0000																										

L4	-0.1181	0.0334	0.1957																									
L5	-0.0167	0.0608	0.1202	0.3998																								
L6	0.0057	0.0617	-0.0486	0.0000	0.0000																							
L7	0.0000	0.0729	0.0000	0.0000	0.0452	0.6487																						
L8	0.0000	0.0167	0.2081	0.0889	0.1128	0.0000	0.0000																					
L9	0.0389	0.0247	-0.0098	0.0000	-0.0373	0.0000	0.0000	-0.0860																				
L10	0.0000	0.1089	-0.0417	-0.0018	0.0000	0.0199	0.0278	0.0000	0.3499																			
L11	0.0000	0.0144	0.0303	0.0218	0.0954	0.0316	0.0000	0.0059	-0.0065	0.0000																		
L12	-0.0114	0.0000	0.0063	0.0243	0.1206	0.0000	0.0012	0.0000	-0.0419	-0.0018	0.5137																	
L13	0.0000	0.0000	0.0000	0.0273	0.0000	-0.0216	-0.0483	0.0000	0.0000	-0.0306	0.0000	0.0233																
L14	0.0000	0.0009	0.0390	0.0370	0.0000	-0.0768	-0.0394	0.0372	0.0000	-0.0221	0.0000	0.0164	0.5885															
L15	0.0000	0.0000	0.0000	0.0000	0.0000	0.0000	0.0000	0.0000	0.0000	0.0063	0.0000	-0.0159	0.0000	0.0000														
L18	0.0000	0.0000	0.0000	0.0000	0.0000	-0.0056	0.0000	0.0000	0.0000	0.0000	0.0000	-0.0056	0.0352	0.0070	0.7847													
H1	-0.0789	0.0547	0.0000	0.0366	0.0423	-0.0208	0.0000	0.0113	0.0000	0.0000	0.0024	0.0091	0.0222	0.0616	0.0000	0.0293												
H2	0.0790	0.0000	0.0000	-0.0668	0.0000	0.0000	0.0194	0.0000	0.0000	0.0000	0.0000	-0.0468	-0.0169	-0.0215	0.0059	0.0000	-0.4151											
H3	0.0000	0.0993	0.0000	0.0000	0.0000	0.0000	0.0000	-0.0105	0.0300	0.0000	0.0000	0.0000	0.0000	0.0000	0.0289	0.0813	0.0000	0.0595										
H4	0.0000	0.0000	0.0000	0.0000	0.0000	0.0000	0.0000	0.0000	0.0000	0.0000	0.0000	0.0000	0.0000	0.0000	0.0073	0.0249	0.0000	0.0824	0.2017									
H5	0.0343	0.0543	0.0092	0.0000	0.0000	0.0000	0.0000	0.0000	0.0000	0.0000	0.0000	0.0000	0.0000	0.0695	0.0000	0.0412	0.0000	0.0000	0.2222	0.3246								
H6	0.0000	0.0000	-0.0196	0.0000	0.0000	0.0000	0.0000	0.0000	0.0000	0.0000	0.0000	0.0214	0.0082	0.0190	0.0000	0.0000	0.0000	-0.0253	0.0000	0.0000	-0.0210							
H7	0.0000	0.0000	-0.0001	0.0000	0.0000	0.0000	-0.0217	0.0000	0.0000	0.0000	0.0000	0.0164	0.0426	0.0155	0.0000	0.0000	0.0041	0.0000	0.0000	0.0000	0.0000	0.6165						
H8	0.0000	0.0000	0.0000	0.0121	0.0326	0.0000	0.0000	0.0000	-0.0227	0.0000	0.0809	0.0000	0.0025	0.0000	0.0000	0.0000	0.0167	-0.0540	0.0000	0.0000	0.0269	0.0212	0.2071					
H9	0.0000	0.0701	0.0000	0.0000	0.0192	0.0302	0.0000	0.0000	0.0289	0.0000	0.0000	0.0000	0.0000	0.0000	0.0000	0.0000	0.0000	0.0269	0.0000	0.0000	0.0377	-0.0528	-0.0051	-0.0227				
H10	0.0000	0.0000	-0.0438	0.0000	-0.0135	0.0000	0.0000	-0.0512	0.0000	0.0403	0.0000	0.0000	0.0210	0.0000	0.0110	0.0000	0.0000	0.0000	0.0000	0.0000	0.0000	0.0000	0.0000	-0.0130	0.3918			
H11	0.0000	0.0000	0.0000	-0.0109	-0.0771	-0.0764	-0.0103	-0.0208	0.0000	0.0000	0.0000	0.0000	0.0085	0.0816	0.0377	0.0267	0.0000	0.0000	0.0000	0.0764	0.1212	0.0000	0.0359	0.0000	0.0456	0.1160		
H12	-0.0168	-0.0177	0.0000	0.0000	0.0000	0.0000	0.0000	0.0288	0.0000	0.0000	0.0000	0.0000	0.0000	0.0000	0.0000	0.0000	0.0000	0.0000	-0.0473	-0.0111	-0.1737	0.0000	-0.0078	-0.0508	0.0000	-0.0722	-0.1206	
H13	0.0000	0.0000	0.0000	0.0000	0.0000	0.0000	0.0229	0.0000	0.0221	0.0034	-0.0012	0.0000	0.0000	0.0000	0.0000	0.0000	-0.0408	0.0000	0.0000	0.0000	0.0000	-0.1286	-0.1016	-0.0766	0.0497	0.0000	0.0000	0.0000

(n) Insomnia

	L1	L2	L3	L4	L5	L6	L7	L8	L9	L10	L11	L12	L13	L14	L17	L18	H1	H2	H3	H4	H5	H6	H7	H8	H9	H10	H11	H12

L1																												
L2	0.2287																											
L3	-0.0025	0.0000																										
L4	-0.0250	0.0471	0.2334																									
L5	-0.0131	0.0475	0.0993	0.4176																								
L6	0.0000	0.0251	0.0000	0.0000	0.0000																							
L7	0.0000	0.0757	0.0000	0.0000	0.0000	0.5593																						
L8	0.0000	0.0000	0.1576	0.0707	0.1357	0.0000	0.0000																					
L9	0.0186	0.1178	0.0000	0.0000	0.0000	0.0331	0.0000	0.0000																				
L10	0.0000	0.0000	0.0000	0.0000	0.0000	0.0495	0.0026	-0.0657	0.3502																			
L11	0.0000	0.0000	0.0000	0.0126	0.0137	0.0000	0.0000	0.0000	0.0000	0.0000																		
L12	0.0000	0.0438	0.0000	0.0000	0.0788	0.0000	0.0000	0.0639	0.0000	0.0000	0.4777																	
L13	0.0000	0.0000	0.0000	0.0000	0.0000	0.0000	-0.0767	0.0000	-0.0143	0.0000	0.0000	0.0445																
L14	0.0000	0.0000	0.0239	0.0000	0.0811	-0.1229	-0.0175	0.0054	0.0000	-0.0111	0.0000	0.0000	0.5280															
L17	0.0000	0.0000	0.0039	0.0000	0.0000	0.0000	0.0000	0.0000	0.0000	0.0000	-0.0343	0.0000	0.0000	0.0000														
L18	0.0000	0.0000	0.0000	0.0000	0.0000	0.0000	0.0000	0.0000	0.0000	0.0000	-0.0233	0.0000	0.0372	0.0000	0.6663													
H1	-0.0572	0.0000	0.0014	0.0182	0.0440	0.0000	0.0000	0.0000	0.0000	0.0000	0.0000	0.0081	0.0154	0.0395	0.0000	0.0000												
H2	0.1416	0.0000	-0.0323	-0.0625	0.0000	0.0000	0.0000	0.0000	0.0000	0.0000	0.0000	-0.0048	-0.0167	0.0000	0.0000	0.0000	-0.3701											
H3	0.0000	0.1504	0.0000	0.0000	0.0000	0.0000	0.0000	0.0000	0.0000	0.0000	0.0000	0.0000	0.0396	0.0000	0.0879	0.0901	0.0000	0.0175										
H4	0.0000	0.0000	0.0000	0.0000	-0.0058	-0.0339	0.0000	0.0000	0.0000	-0.0329	0.0000	0.0000	0.0000	0.0000	0.0000	0.0784	0.0000	0.0283	0.0873									
H5	0.0000	0.0400	0.0000	0.0000	0.0000	0.0000	0.0000	0.0000	0.0000	0.0000	0.0000	0.0000	0.0000	0.0000	0.0102	0.0470	0.0000	0.0000	0.2844	0.3328								
H6	-0.0152	0.0000	0.0000	0.0000	0.0000	0.0000	0.0000	0.0000	0.0000	0.0000	0.0000	0.0000	0.0134	0.0000	-0.0141	0.0000	0.0000	-0.0110	0.0000	0.0000	-0.0230							
H7	0.0000	0.0000	0.0000	0.0000	0.0000	0.0000	0.0000	0.0000	0.0000	0.0000	0.0000	0.0000	0.0326	0.0627	-0.0003	0.0000	0.0000	0.0000	0.0000	0.0000	0.0000	0.6202						
H8	0.0000	0.0000	0.0000	0.0125	0.0206	0.0000	0.0000	0.0000	0.0000	0.0000	0.0000	0.0000	0.0200	0.0327	-0.0232	-0.0047	0.0000	-0.0016	0.0000	0.0000	0.0000	0.0586	0.2012					
H9	0.0000	0.0000	0.0000	0.0000	0.0000	0.0000	0.0000	0.0000	0.0250	0.0059	0.0000	0.0000	-0.0144	0.0000	0.0000	0.0000	0.0000	0.0000	0.0000	0.0000	0.0454	-0.0576	0.0000	0.0000				
H10	0.0000	0.0000	0.0000	0.0000	0.0000	0.0000	0.0000	0.0000	0.0000	0.0000	0.0000	0.0000	0.0000	0.0000	0.0000	0.0160	0.0000	0.0000	0.0000	0.0000	0.0000	0.0000	0.0000	0.0000	0.2799			
H11	0.0000	0.0000	0.0000	0.0000	0.0000	-0.0372	-0.0197	0.0000	-0.0234	0.0000	0.0000	0.0000	0.0190	0.0563	0.0564	0.0026	0.0000	0.0000	0.0093	0.0600	0.0874	0.0000	0.0000	0.0000	0.0208	0.1951		
H12	0.0000	0.0000	0.0000	0.0000	0.0000	0.0000	0.0000	0.0270	0.0000	0.0000	0.0000	0.0000	0.0000	0.0000	0.0000	-0.0009	0.0000	0.0000	-0.0958	0.0000	-0.1220	0.0000	0.0000	0.0000	-0.0710	-0.0296	-0.1213	
H13	0.0000	0.0000	0.0000	0.0000	0.0000	0.0000	0.0043	0.0000	0.0000	0.0000	0.0000	0.0000	-0.0513	0.0000	0.0000	0.0000	0.0000	0.0000	0.0000	0.0000	0.0000	-0.0776	-0.0703	-0.1466	0.0000	0.0000	0.0222	0.0000

(o) Irritable bowel syndrome

	L1	L2	L3	L4	L5	L6	L7	L8	L10	L11	L12	L13	L14	L15	L16	L18	H1	H2	H3	H4	H5	H7	H8	H9	H10	H11	H12
L1																											
L2	0.3196																										
L3	0.0000	0.0000																									
L4	0.0000	0.0412	0.1527																								
L5	0.0000	0.0000	0.1266	0.3973																							
L6	0.0000	0.0199	0.0000	0.0000	0.0000																						
L7	0.0000	0.1534	0.0000	0.0000	0.0190	0.5467																					
L8	0.0000	0.0000	0.1343	0.0000	0.1407	0.0000	0.0538																				
L10	0.0132	0.0954	0.0000	0.0000	0.0000	0.0127	0.0000	0.0000																			
L11	0.0000	0.0000	0.0366	0.0000	0.1335	0.0000	0.0000	0.0235	-0.0309																		
L12	-0.0080	0.0000	0.0365	0.1231	0.0473	0.0086	0.0000	0.0000	-0.0374	0.4365																	
L13	0.0000	0.0000	0.0000	0.0000	0.0168	-0.0014	0.0000	0.0000	-0.0202	0.0000	0.0000																
L14	0.0000	0.0000	0.0000	0.0249	0.0000	-0.1647	0.0000	0.0000	-0.0226	0.0000	0.0000	0.6224															
L15	0.0000	0.0000	0.0000	0.0000	0.0000	0.0000	0.0000	0.0000	0.0000	0.0000	0.0000	0.0000	0.0000														
L16	0.0000	0.0000	0.0000	0.0000	0.0000	0.0000	0.0000	0.0000	0.0000	0.0000	0.0000	0.0147	0.0000	0.4670													
L18	0.0000	0.0614	0.0029	0.0000	0.0000	0.0000	0.0000	0.0000	0.0000	0.0000	0.0000	0.0147	0.0000	0.3696	0.4764												
H1	-0.0217	0.0000	0.0031	0.0522	0.0328	0.0000	0.0000	0.0514	0.0000	0.0000	0.0193	0.0467	0.0385	0.0351	0.0000	0.0000											
H2	0.1503	0.0000	-0.0084	-0.1046	0.0000	0.0000	0.0000	-0.0131	0.0000	0.0000	-0.0266	-0.0581	-0.0223	0.0000	0.0000	0.0000	-0.3254										
H3	0.0089	0.0305	0.0000	0.0000	0.0000	0.0000	0.0000	0.0000	0.0000	0.0000	0.0000	0.0000	0.0000	0.0175	0.0351	0.0569	0.0000	0.0020									
H4	0.0000	0.0000	0.0000	0.0000	0.0000	0.0000	0.0000	0.0000	-0.0284	0.0000	0.0000	0.0000	0.0000	0.0058	0.0148	0.0000	0.0000	0.0924	0.2075								
H5	0.0033	0.0000	0.0000	0.0000	0.0000	0.0000	0.0000	0.0000	0.0000	0.0197	0.0000	0.0364	0.0000	0.0477	0.0000	0.0339	0.0000	0.0000	0.2486	0.2878							
H7	-0.0136	0.0000	0.0000	0.0000	0.0000	0.0000	0.0000	0.0000	0.0000	0.0132	0.0000	0.0229	0.0396	0.0000	0.0000	0.0000	0.0000	-0.0226	0.0000	0.0000	0.0000						
H8	0.0000	0.0000	0.0000	0.0474	0.0000	0.0000	0.0000	0.0000	0.0000	0.0000	0.0000	0.0835	0.0193	0.0000	0.0000	0.0000	0.0000	-0.0172	0.0000	0.0000	0.0354	0.3224					
H9	0.0000	0.0000	0.0000	0.0000	0.0000	0.0000	0.0000	0.0000	0.0000	0.0000	0.0000	0.0000	0.0000	0.0000	0.0000	0.0000	0.0000	0.0000	0.0000	0.0000	0.0249	-0.0225	0.0000				
H10	0.0000	0.0000	0.0000	0.0000	0.0000	0.0000	0.0104	0.0000	0.0251	0.0000	0.0000	0.0000	0.0000	0.0378	0.0000	0.0011	0.0000	0.0000	0.0211	0.0000	0.0000	0.0000	0.0000	0.3743			
H11	0.0000	0.0000	0.0000	0.0000	0.0000	-0.0185	-0.0606	-0.0517	0.0000	0.0000	0.0000	0.0082	0.0000	0.0308	0.0234	0.0000	0.0000	0.0000	0.0000	0.1145	0.1809	0.0000	0.0000	0.0223	0.0857		

H12	0.0000	0.0000	0.0000	0.0000	0.0000	0.0000	0.0000	0.0426	-0.0089	0.0000	0.0000	0.0000	0.0000	0.0000	0.0000	0.0000	0.0000	0.0000	-0.0163	0.0000	-0.0858	0.0335	0.0000	0.0000	-0.0367	-0.0286	
H13	0.0000	0.0000	0.0000	0.0000	0.0000	0.0000	0.0000	0.0000	0.0912	0.0000	0.0000	0.0000	0.0000	0.0000	0.0000	-0.0055	0.0000	0.0000	0.0000	0.0000	0.0000	-0.1601	0.0000	0.0000	0.0000	0.0000	0.0000

(p) Pre-diabetes

	L1	L2	L3	L4	L5	L6	L8	L9	L10	L11	L12	L13	L14	L18	H1	H2	H3	H4	H5	H6	H7	H8	H9	H10	H11	H12
L1																										
L2	0.1491																									
L3	0.0000	0.0000																								
L4	-0.0613	0.0205	0.0807																							
L5	0.0000	0.0093	0.0488	0.4390																						
L6	0.0000	0.1160	0.0000	0.0000	0.0000																					
L8	0.0000	0.0000	0.1146	0.0589	0.1780	0.0000																				
L9	0.0000	0.0000	0.0000	0.0000	0.0000	0.0000	0.0000																			
L10	0.0000	0.0510	-0.0228	0.0000	0.0000	0.0052	0.0000	0.2560																		
L11	0.0000	0.0793	0.0000	0.0019	0.0727	0.0000	0.0000	0.0000	0.0000																	
L12	0.0000	0.0333	0.0135	0.0579	0.0613	0.0000	0.0566	0.0000	0.0000	0.4088																
L13	0.0000	0.0000	0.0000	0.0000	0.0000	-0.0442	0.0000	0.0000	0.0000	0.0000	0.0216															
L14	0.0000	0.0000	0.0508	0.0091	0.0000	-0.1654	0.0000	0.0000	0.0000	0.0158	0.0000	0.5495														
L18	0.0000	0.0000	0.0045	0.0000	0.0000	0.0000	0.0000	0.0000	0.0000	0.0000	0.0000	0.0645	0.0227													
H1	-0.0565	0.0000	0.0000	0.0336	0.0948	0.0000	0.0000	0.0000	0.0000	0.0000	0.0598	0.0000	0.1174	0.0000												
H2	0.1563	0.0000	0.0000	0.0000	0.0000	0.0057	0.0000	0.0000	0.0000	0.0000	0.0000	-0.0288	-0.0008	0.0000	-0.3408											
H3	0.0000	0.0000	0.0000	0.0000	0.0000	0.0000	0.0000	0.0000	0.0000	0.0000	0.0000	0.0000	0.0000	0.1403	0.0000	0.0000										
H4	0.0000	0.0000	0.0000	0.0000	0.0000	0.0000	0.0000	0.0000	0.0000	0.0000	0.0000	0.0000	0.0000	0.0324	0.0000	0.0000	0.1214									
H5	0.0000	0.0861	0.0000	0.0000	0.0000	0.0000	0.0000	0.0000	0.0000	0.0000	0.0000	0.0152	0.0152	0.0363	0.0000	0.0000	0.2155	0.2127								
H6	0.0000	0.0000	0.0000	0.0000	0.0000	0.0000	0.0000	0.0000	0.0000	0.0000	0.0000	0.0115	0.0817	0.0000	0.0000	0.0000	0.0000	0.0000	0.0000							
H7	0.0000	0.0000	0.0000	0.0000	0.0000	-0.0530	0.0000	0.0000	0.0000	0.0000	0.0000	0.0000	0.0163	0.0000	0.0000	-0.0329	0.0000	0.0000	0.0000	0.5351						
H8	0.0000	0.0000	0.0000	0.0433	0.0139	-0.0016	0.0000	0.0000	0.0000	0.0000	0.0000	0.0000	0.0000	0.0000	0.0000	0.0000	0.0000	0.0000	0.0000	0.0226	0.2264					
H9	0.0000	0.0898	0.0000	0.0000	0.0000	0.0000	0.0000	0.0379	0.0000	0.0000	0.0000	0.0000	0.0000	0.0000	0.0000	0.0000	0.0577	0.0000	0.0172	0.0000	0.0000	0.0000				
H10	0.0000	0.0000	0.0000	0.0000	0.0000	0.0000	0.0000	0.0000	0.0000	0.0000	0.0000	0.0000	0.0000	0.0503	0.0000	0.0000	0.0000	0.0000	0.0255	0.0000	0.0000	0.0000	0.3193			

H11	0.0000	0.0000	0.0000	0.0000	0.0000	-0.0004	0.0000	0.0000	0.0000	0.0000	0.0000	0.0348	0.0000	0.0759	0.0000	0.0000	0.0000	0.0716	0.1254	0.0000	0.0666	0.0000	0.0324	0.0000		
H12	0.0000	0.0000	0.0000	0.0000	0.0000	0.0000	0.0000	0.0000	0.0000	0.0000	0.0000	0.0000	0.0000	0.0000	0.0000	0.0000	0.0000	0.0000	-0.1127	0.0000	0.0000	0.0000	0.0000	0.0000	-0.0319	
H13	0.0000	0.0000	0.0000	0.0000	0.0000	0.0000	0.0000	0.0000	0.0000	0.0000	0.0000	0.0000	0.0000	0.0000	0.0000	0.0000	0.0000	0.0000	0.0000	-0.0727	-0.0664	-0.0214	0.0000	0.0000	0.0000	0.0000

Figure S1. Age and gender distribution of sample versus population across countries and overall.

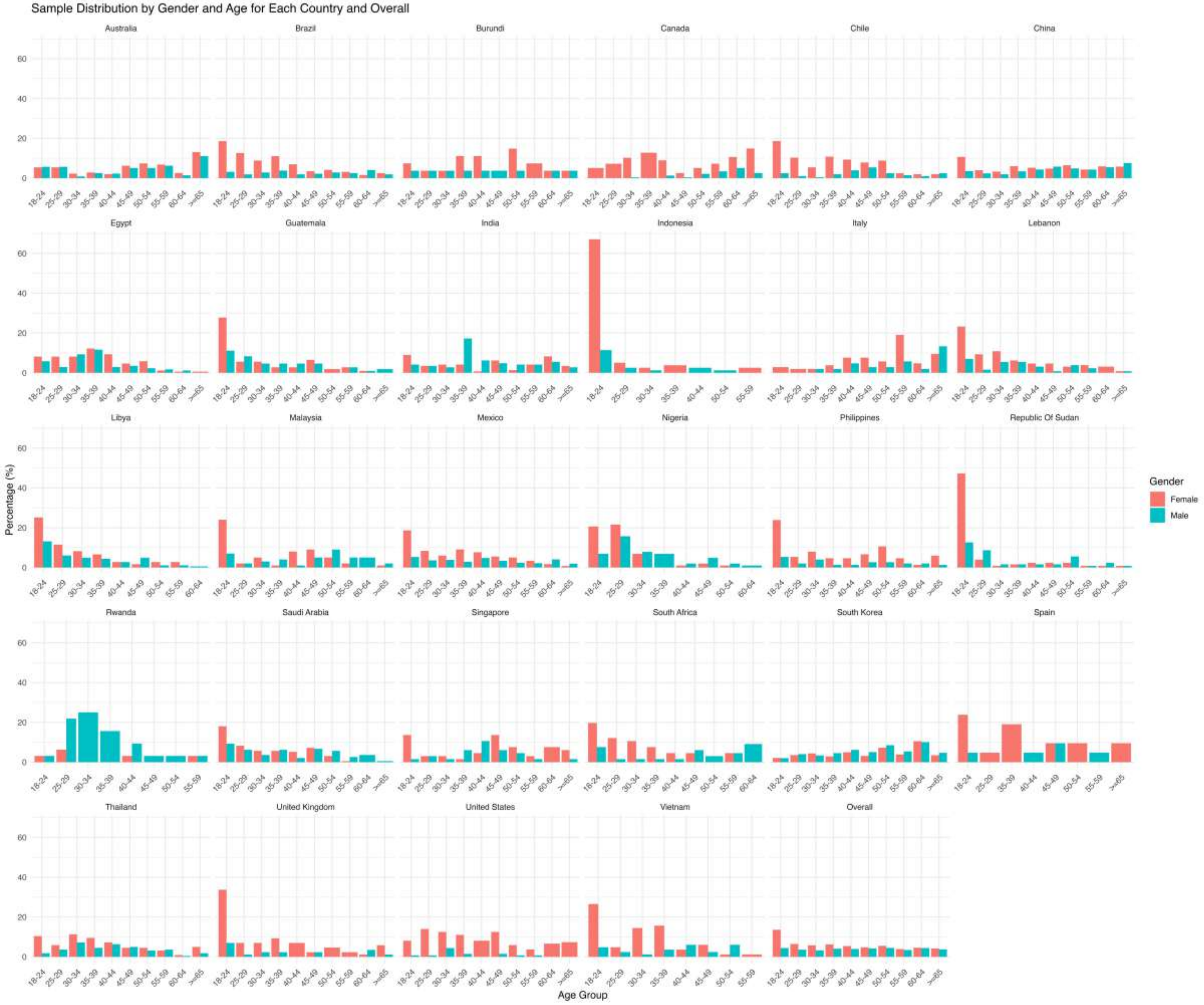

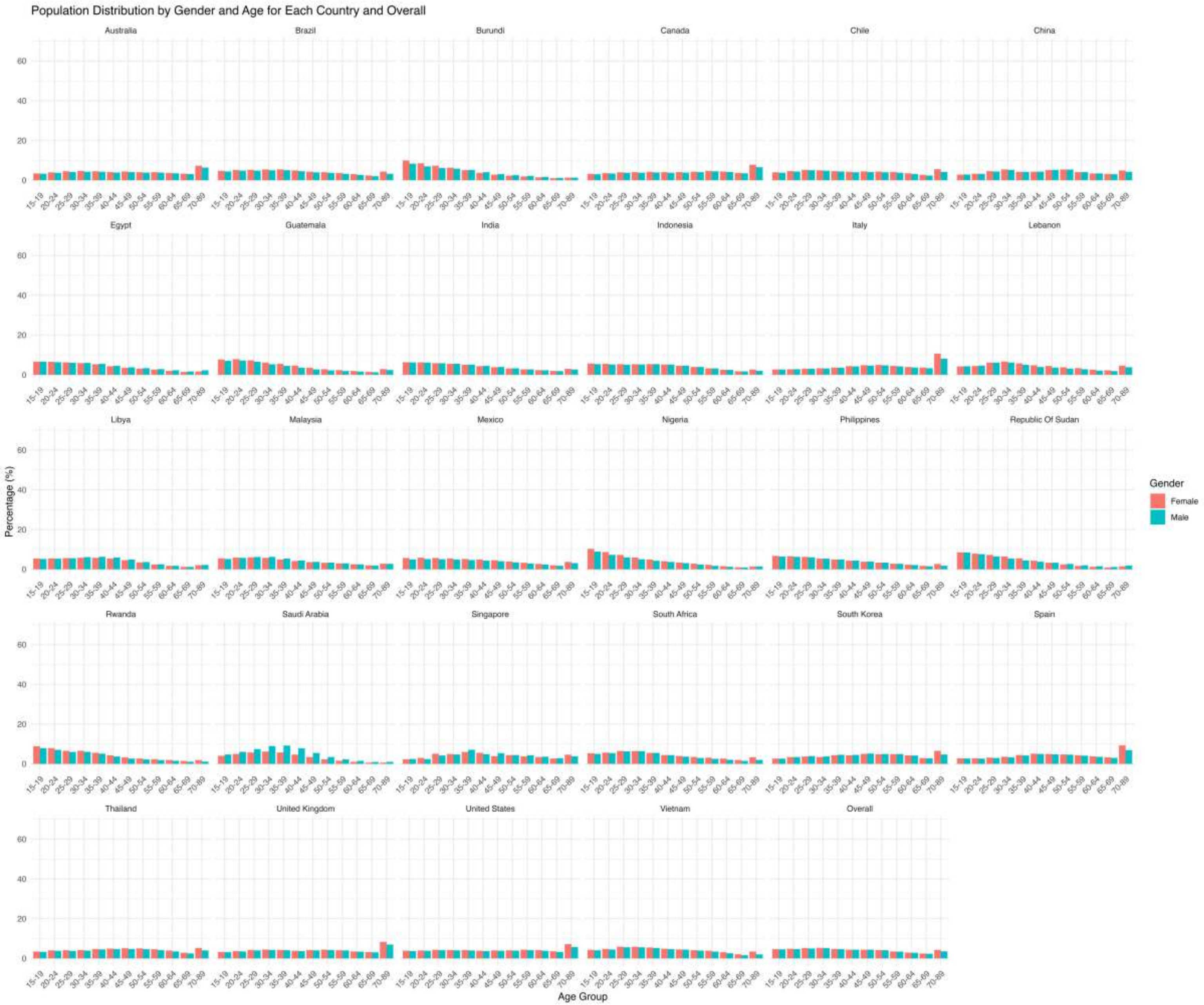

National data were sourced from the Global Burden of Disease study for non-communicable diseases in each country. 'Overall' data represent an aggregate of all countries included in this study.

Figure S2. Bootstrapped confidence intervals of edge weights for the lifestyles network across chronic disease subgroups.

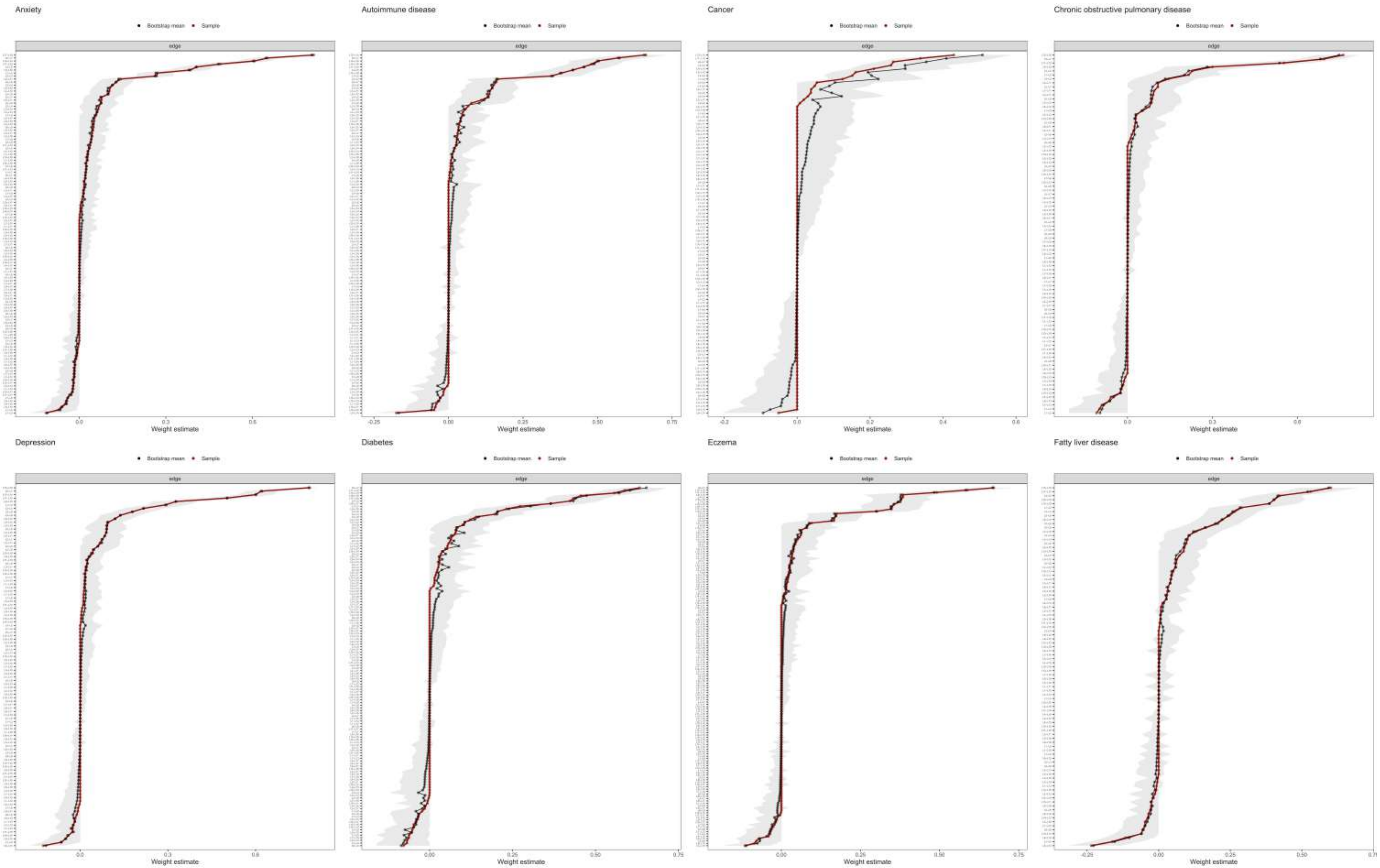

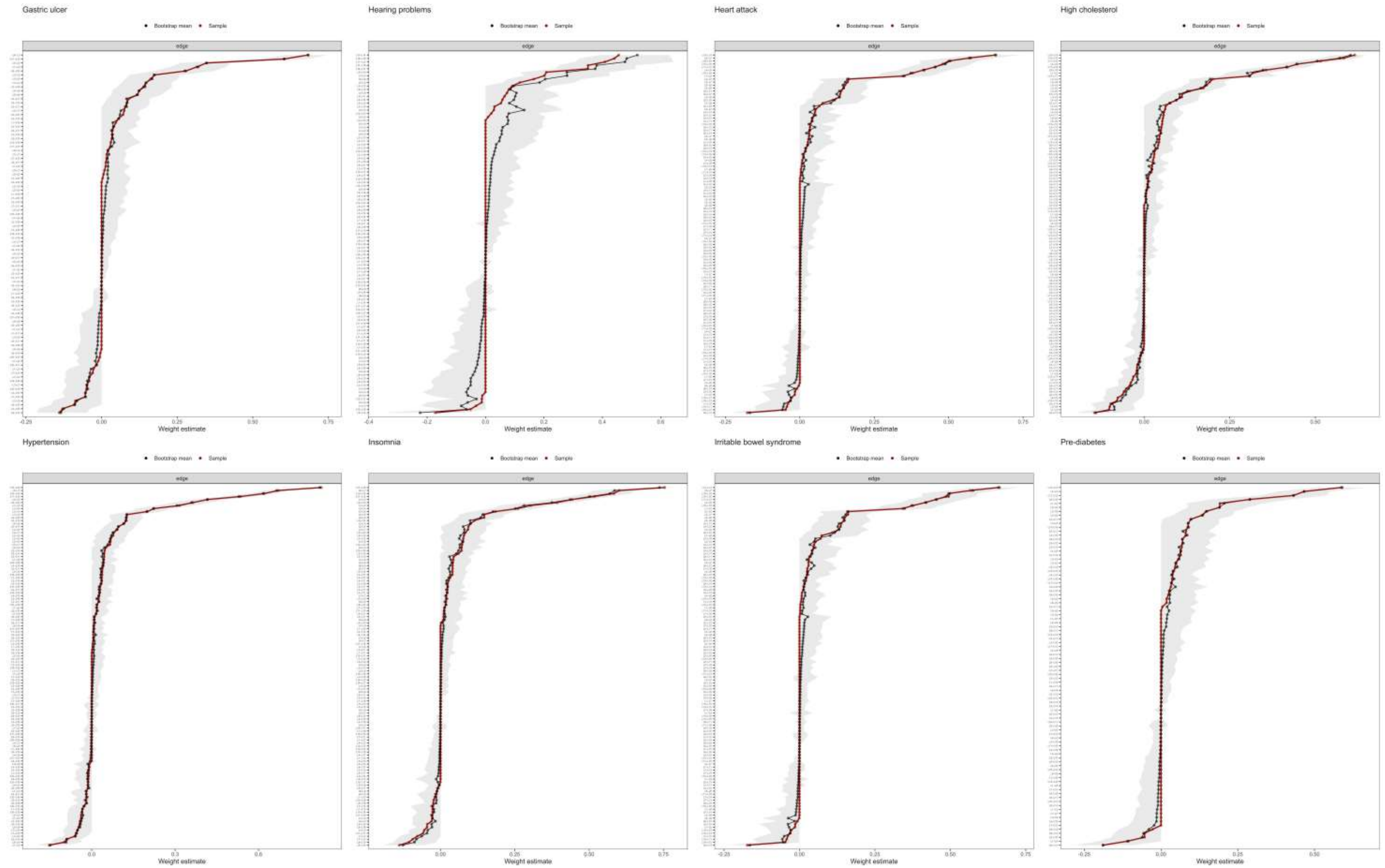

The red dots are sample means per edge, while the black dots are bootstrapped means, ordered from the highest to the lowest value. The gray area represents the 95% confidence intervals of edge weights, estimated with the non-parametric bootstrap procedure (Bootnet package). Wide intervals indicate lower stability and narrow intervals indicate higher stability.

Figure S3. The stability of expected influence centrality index in lifestyles network across chronic disease subgroups using case-dropping bootstrap.

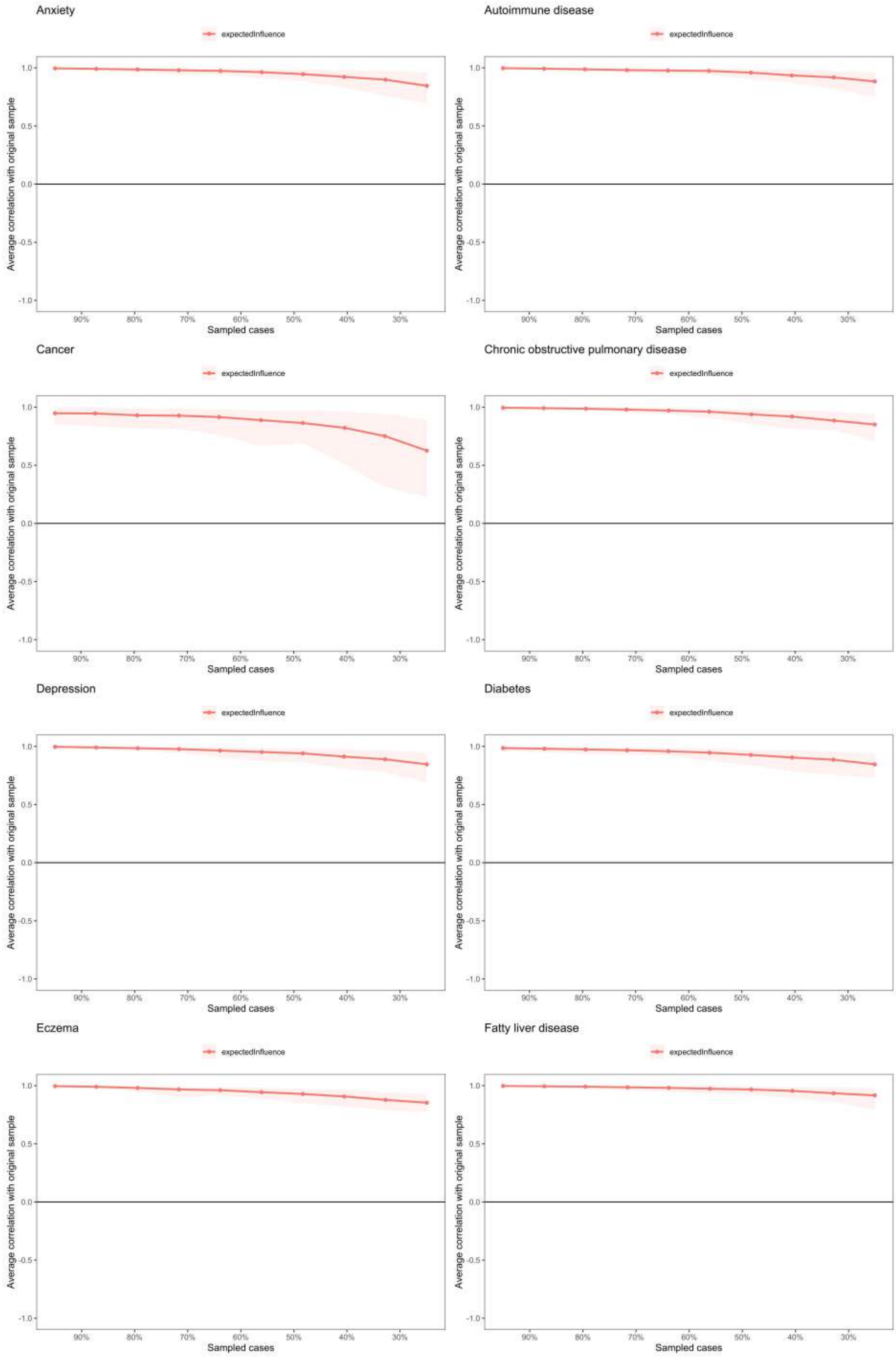

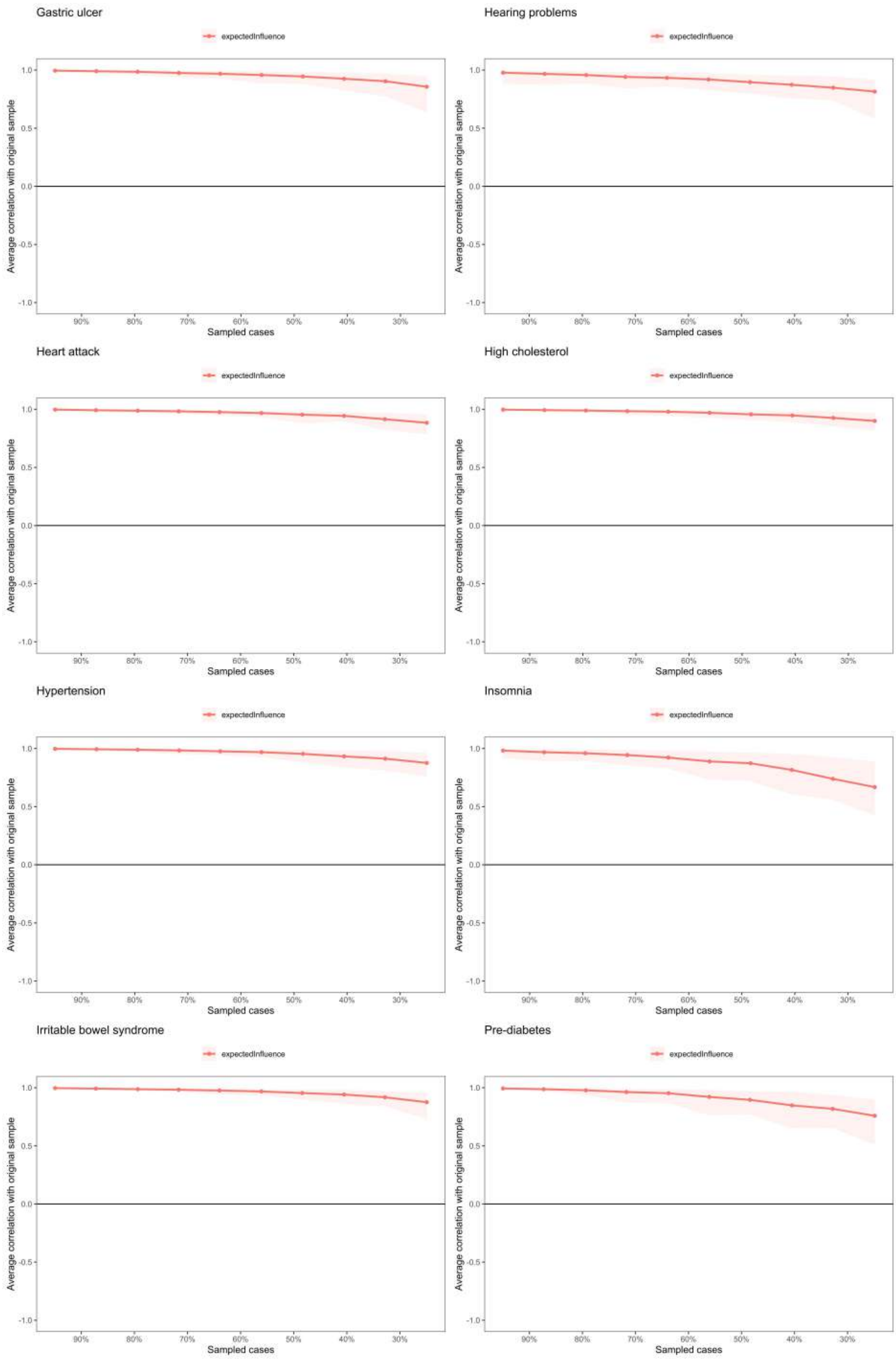

The x-axis indicates the percentage of cases of the original sample included at each step. The y-axis indicates the average of correlations between the expected influence centrality index from the original network and the expected influence centrality index from the networks that were re-estimated after excluding increasing percentages of cases.

Figure S4. Network structure and centrality difference test of lifestyle (A and B), health outcomes (C and D), and combined two (E and F) across chronic disease subgroups.

(a) Anxiety

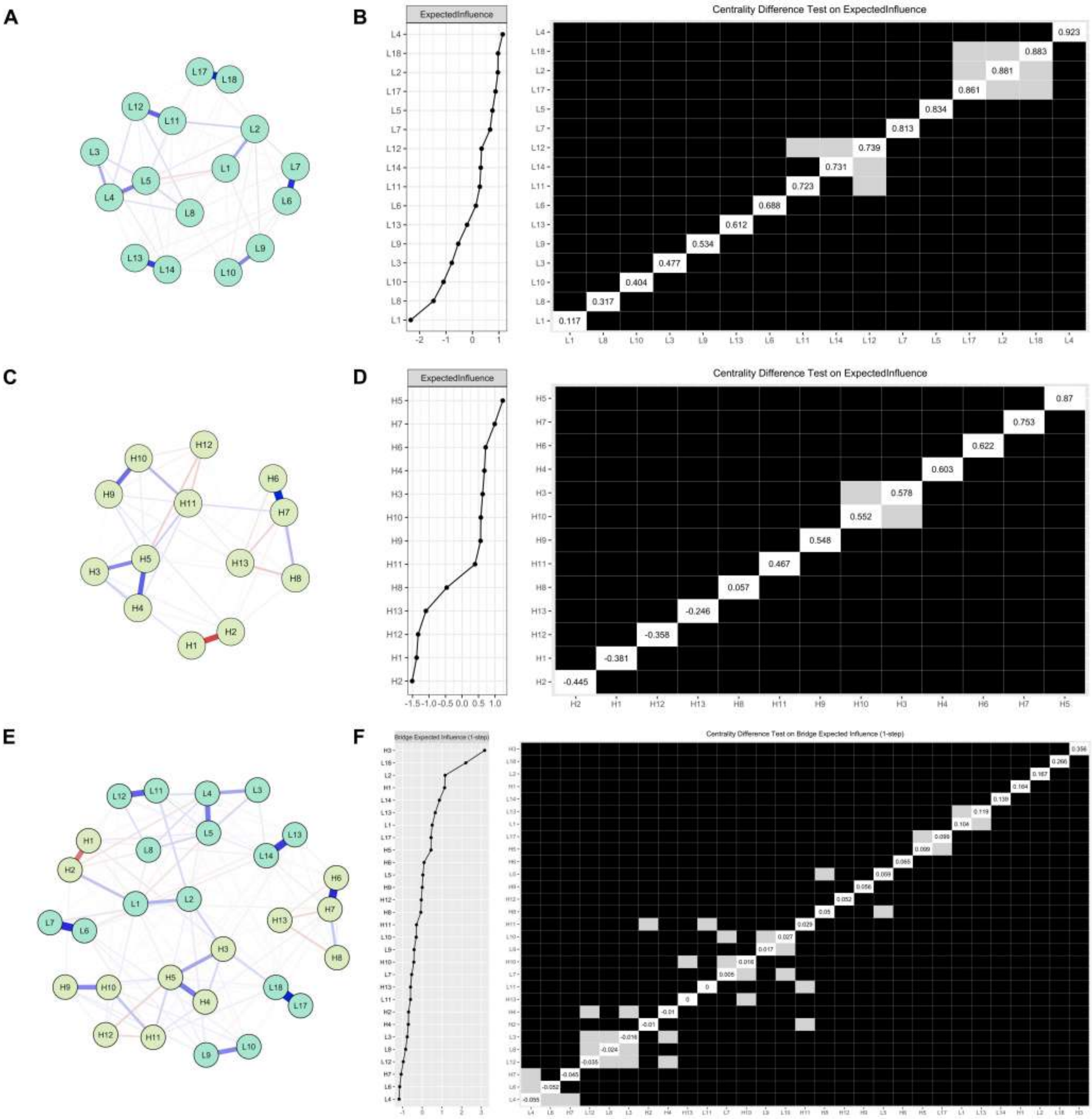

(b) Autoimmune disease

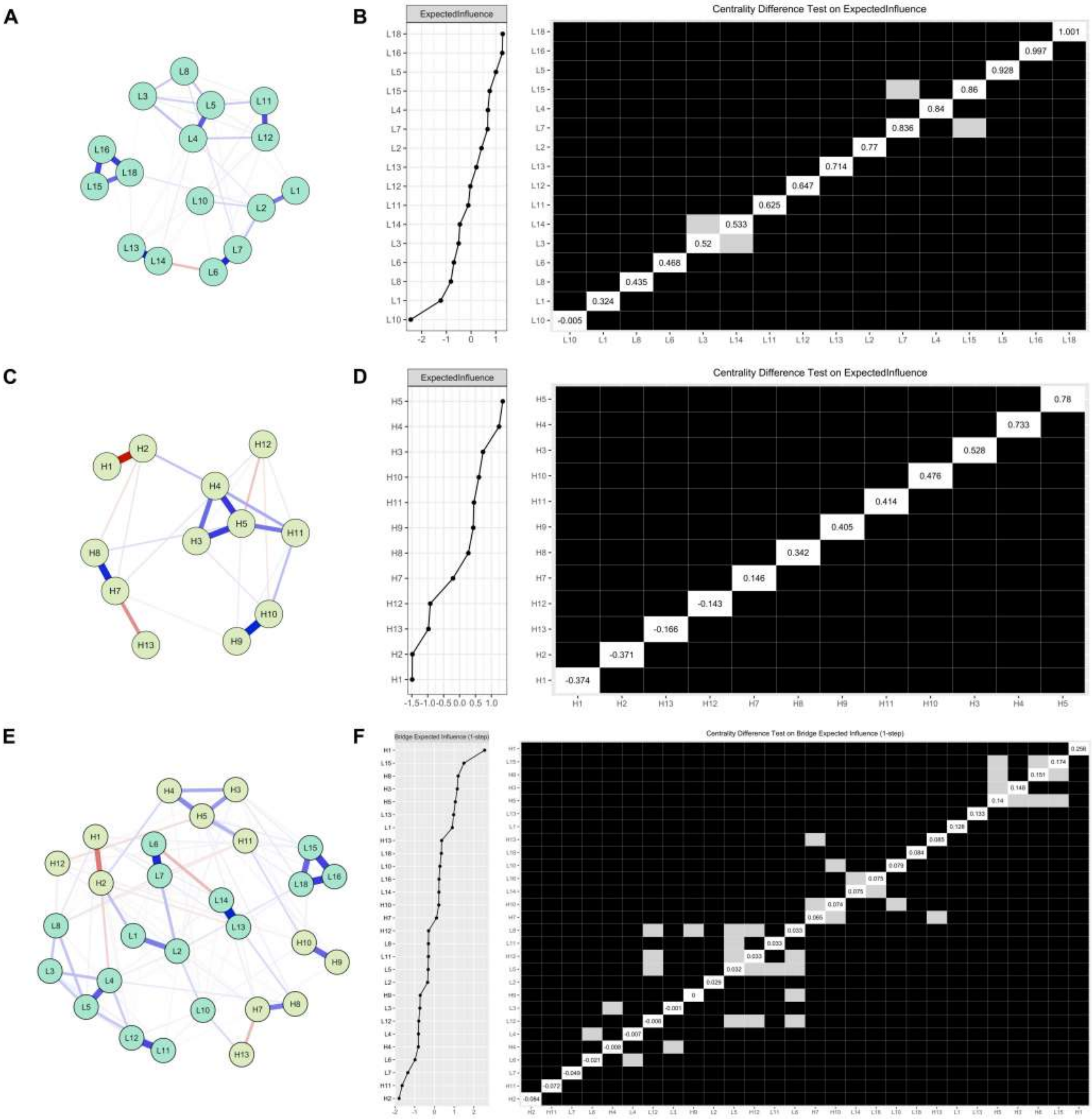

(c) Cancer

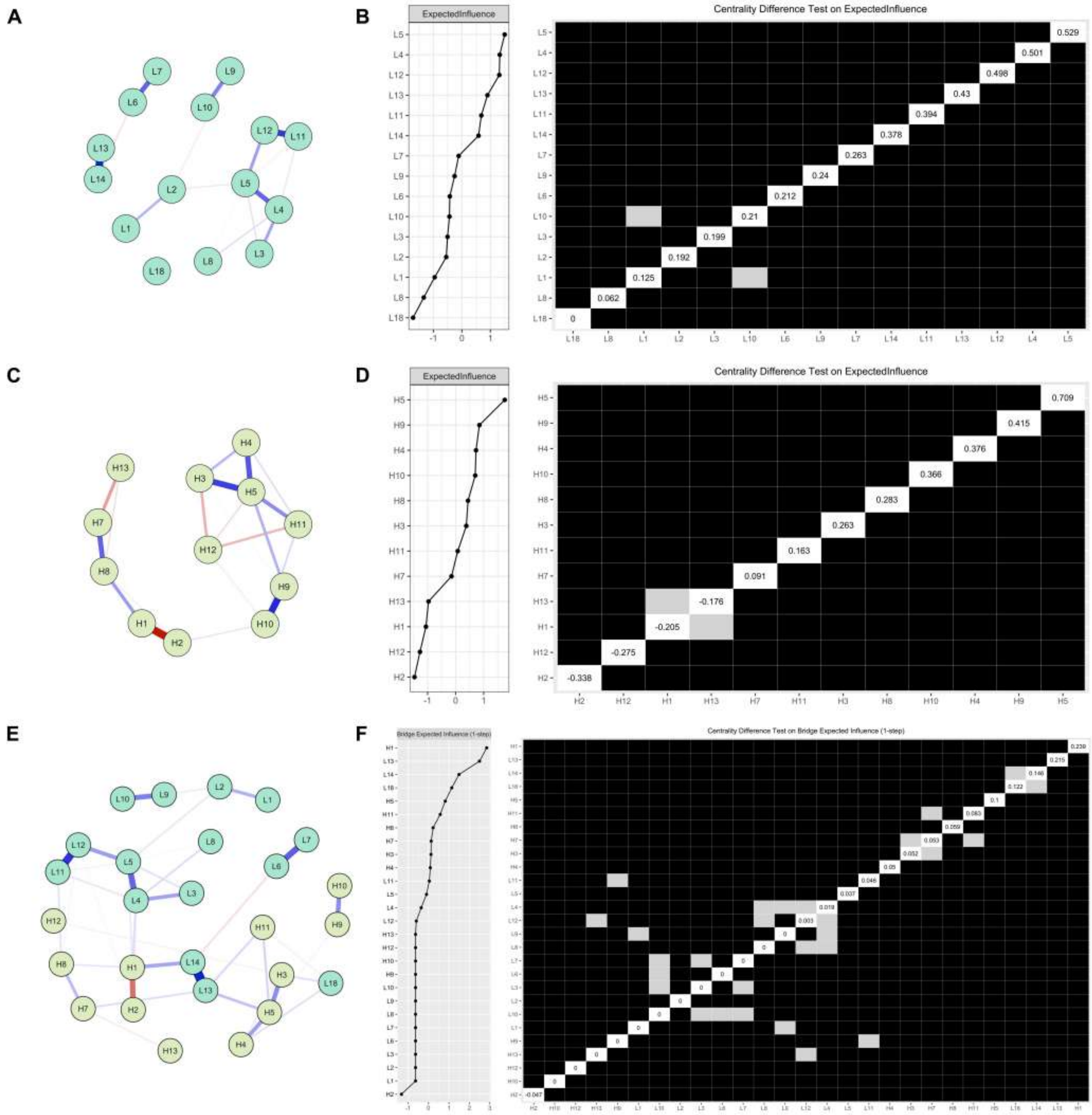
A
B
ExpectedInfluence
Centrality Difference Test on ExpectedInfluence
C
D
ExpectedInfluence
Centrality Difference Test on ExpectedInfluence
E
F
Bridge Expected Influence (1-step)
Centrality Difference Test on Bridge Expected Influence (1-step)

(d) Chronic obstructive pulmonary disease

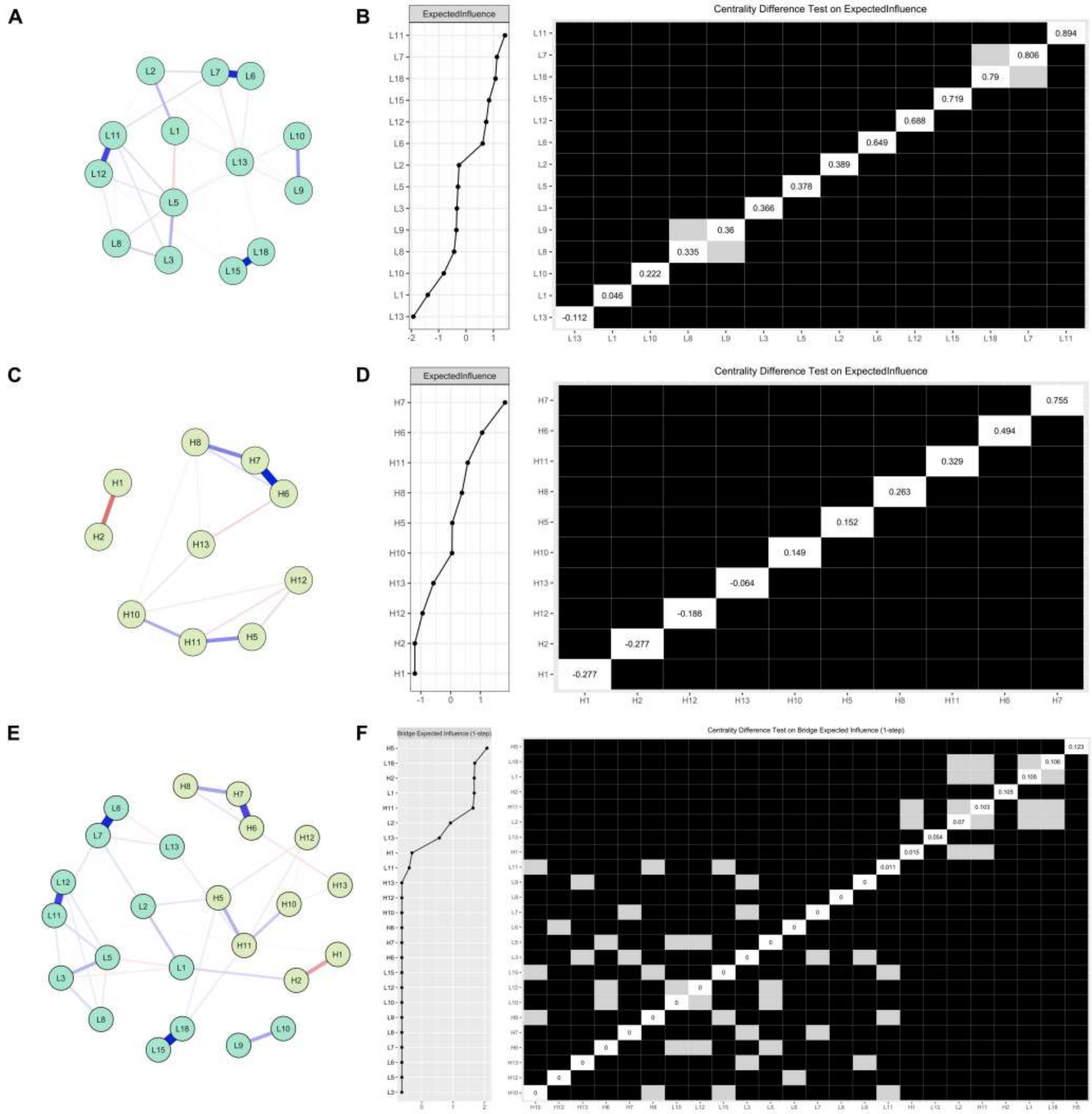

(e) Depression

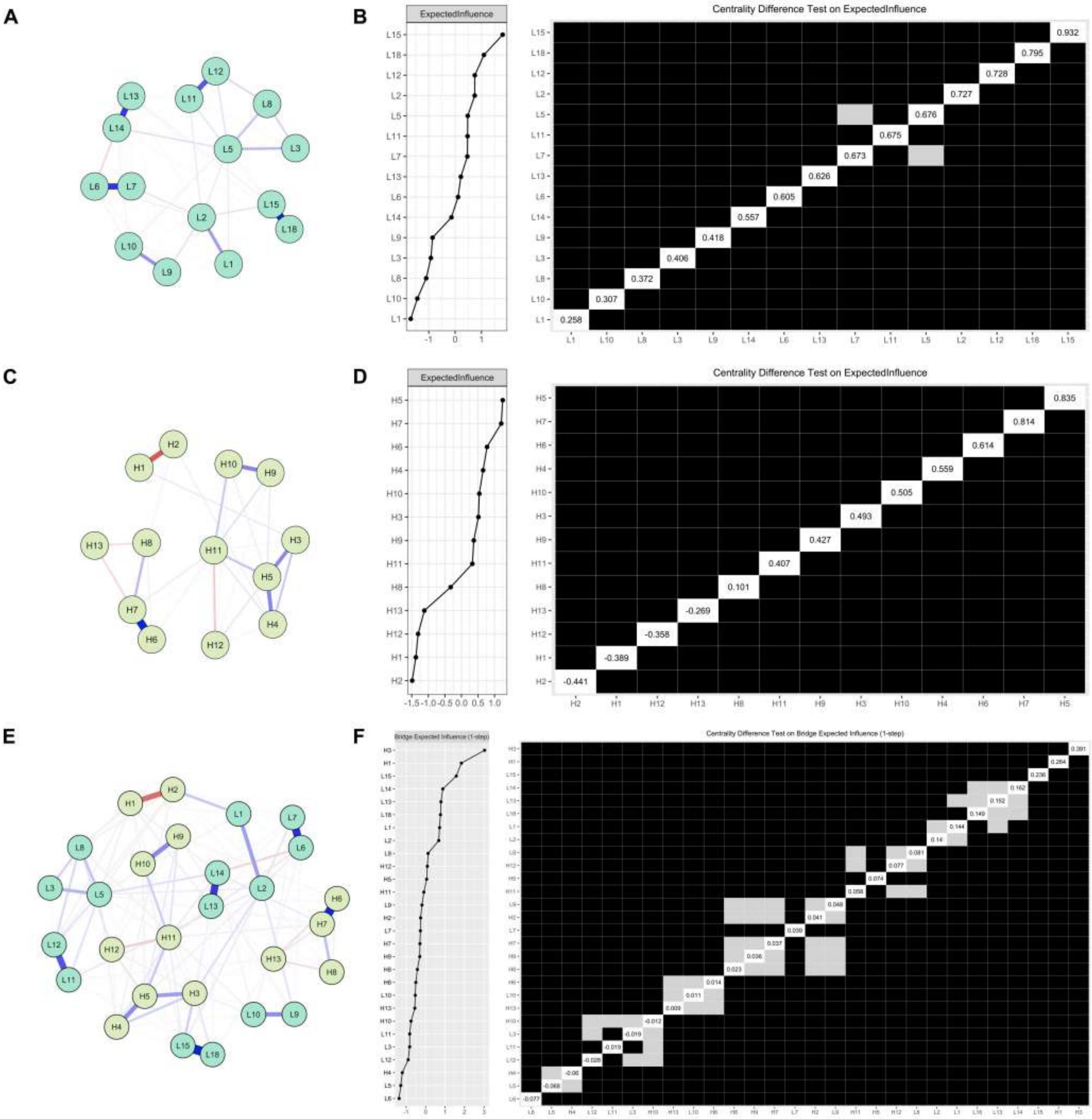

(f) Diabetes

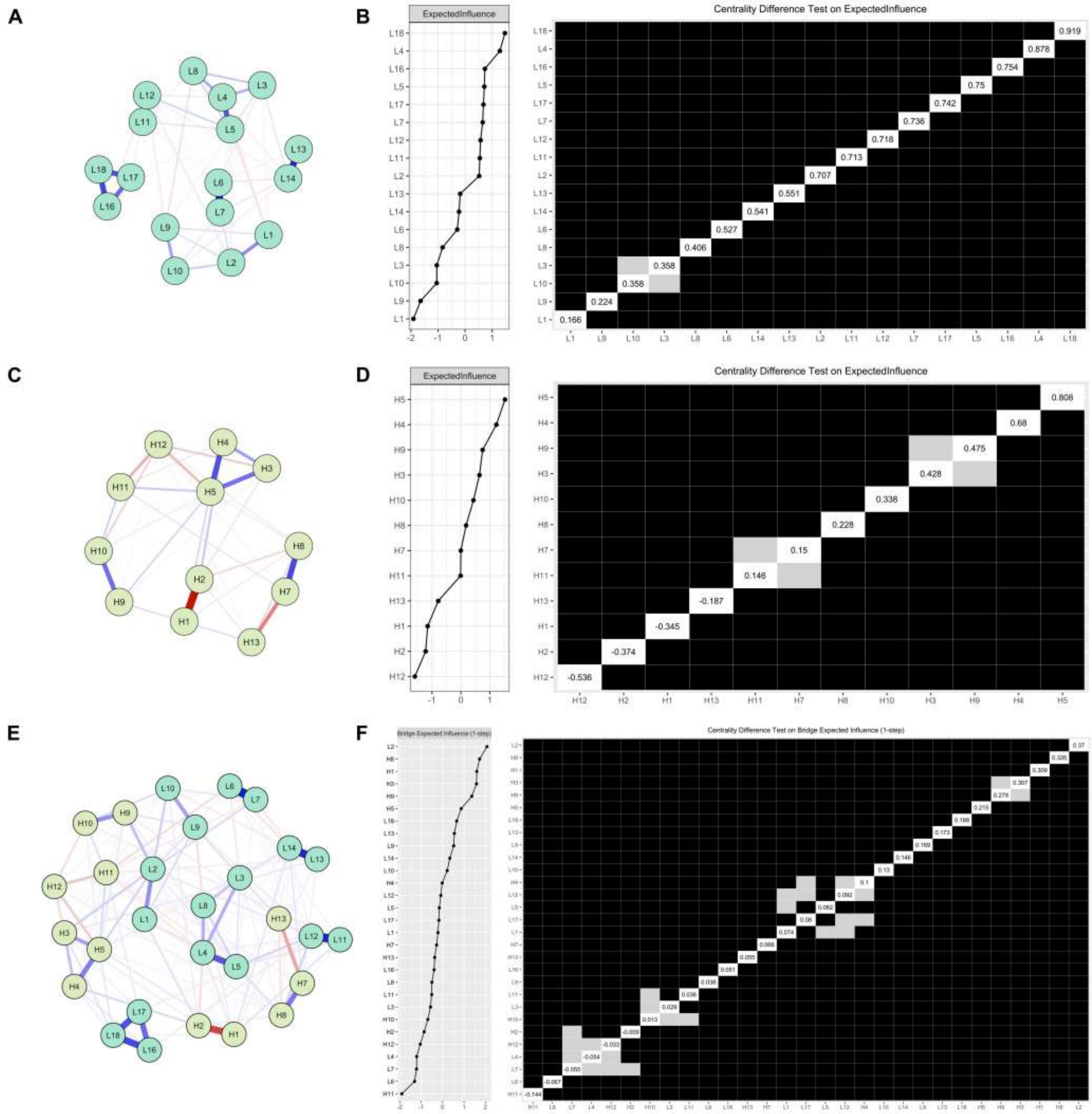

A
B
ExpectedInfluence
Centrality Difference Test on ExpectedInfluence
C
D
ExpectedInfluence
Centrality Difference Test on ExpectedInfluence
E
F
Bridge Expected Influence (1-step)
Centrality Difference Test on Bridge Expected Influence (1-step)

(g) Eczema

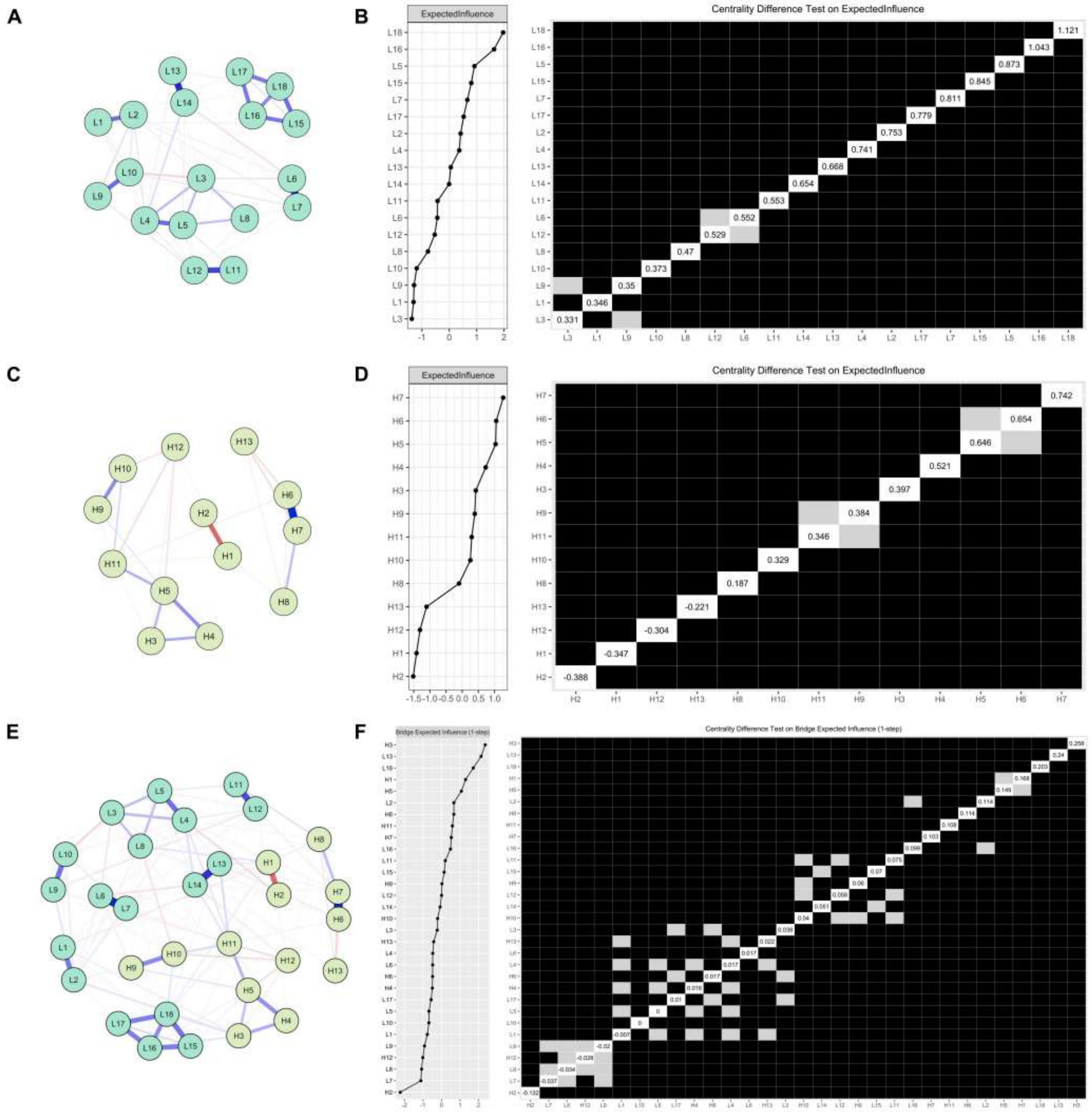

A
B
ExpectedInfluence
Centrality Difference Test on ExpectedInfluence
C
D
E
F
Bridge Expected Influence (1-step)
Centrality Difference Test on Bridge Expected Influence (1-step)

(h) Fatty liver disease

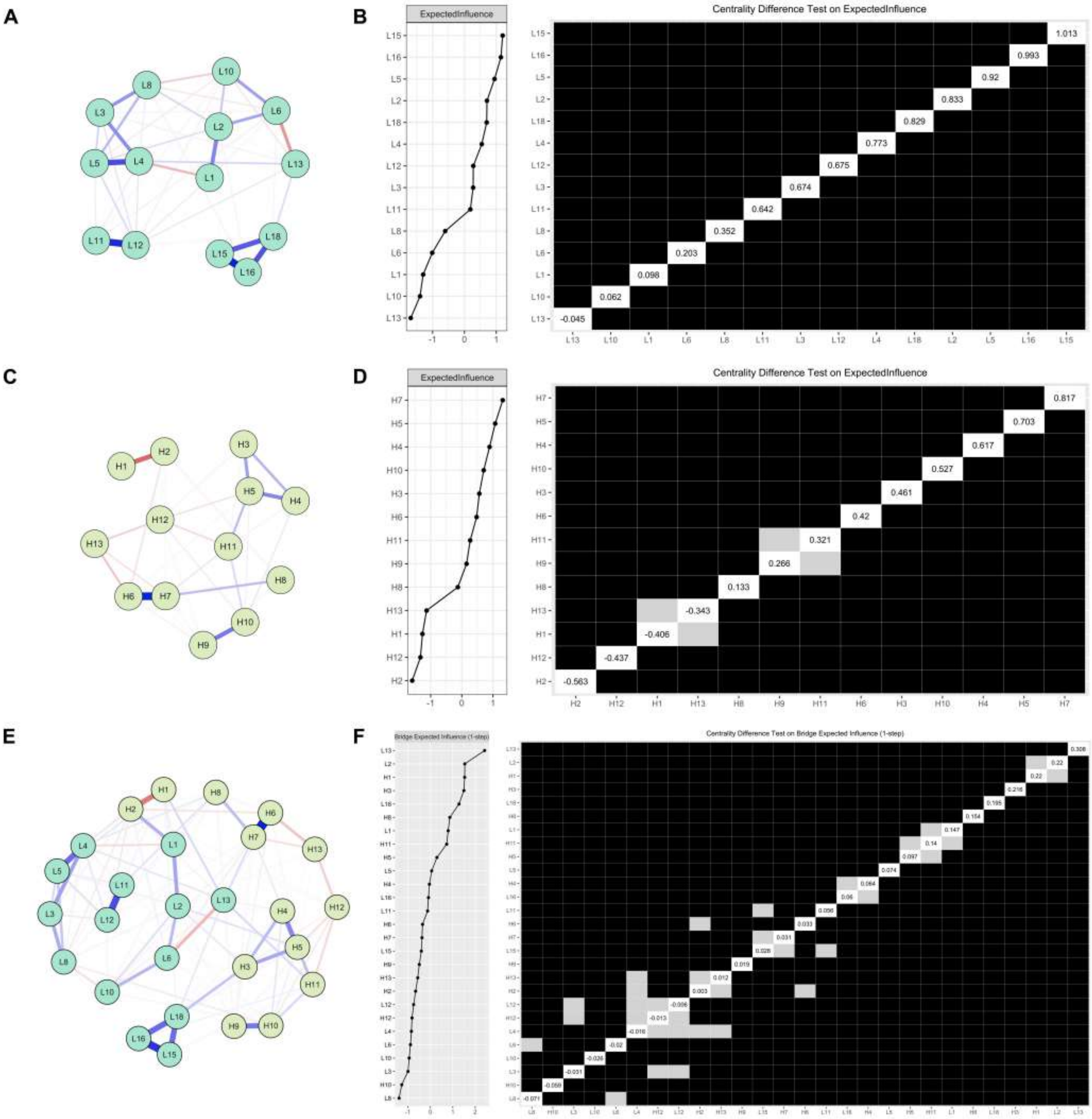

(i) Gastric ulcer

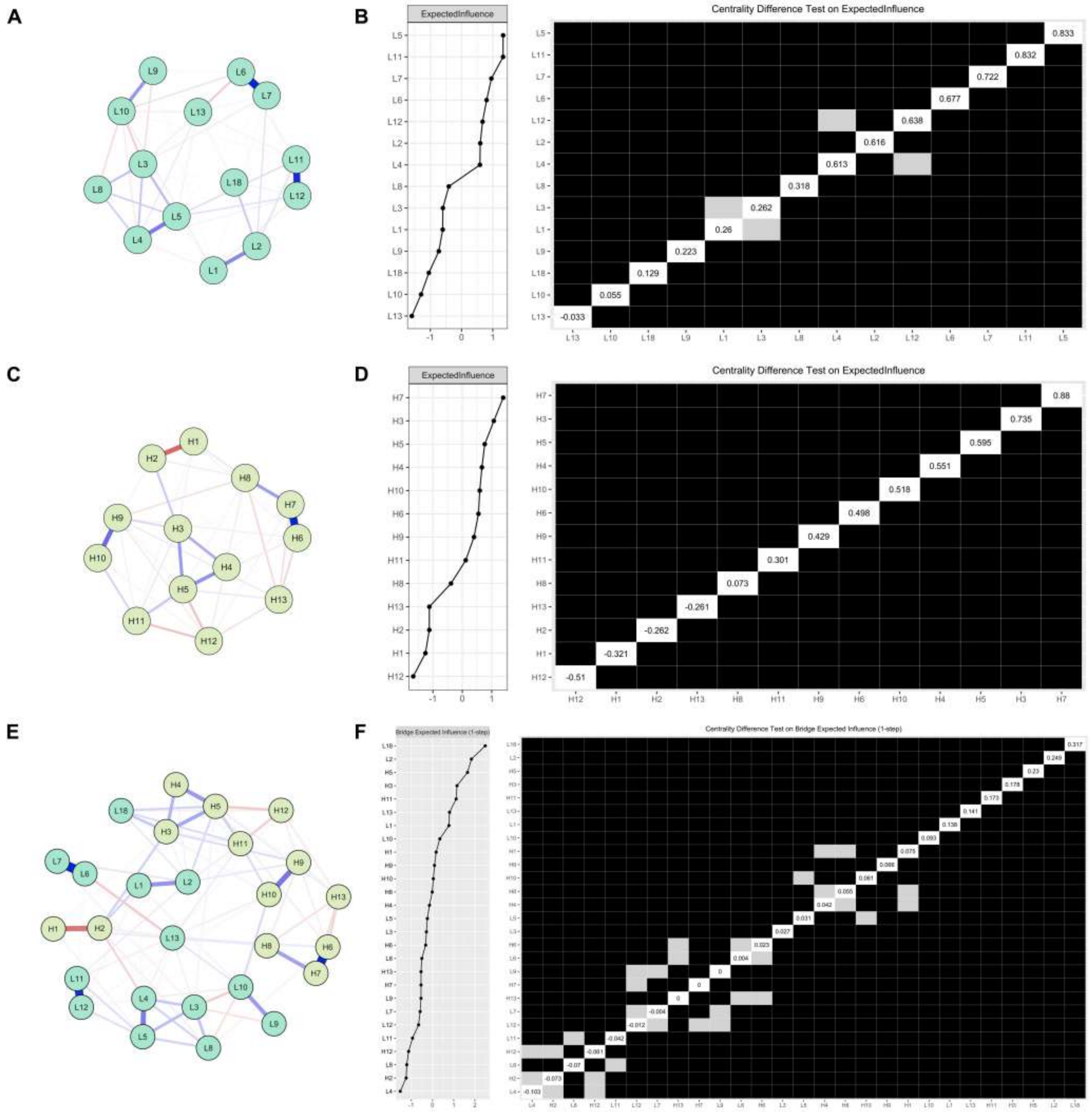

(j) Hearing problems

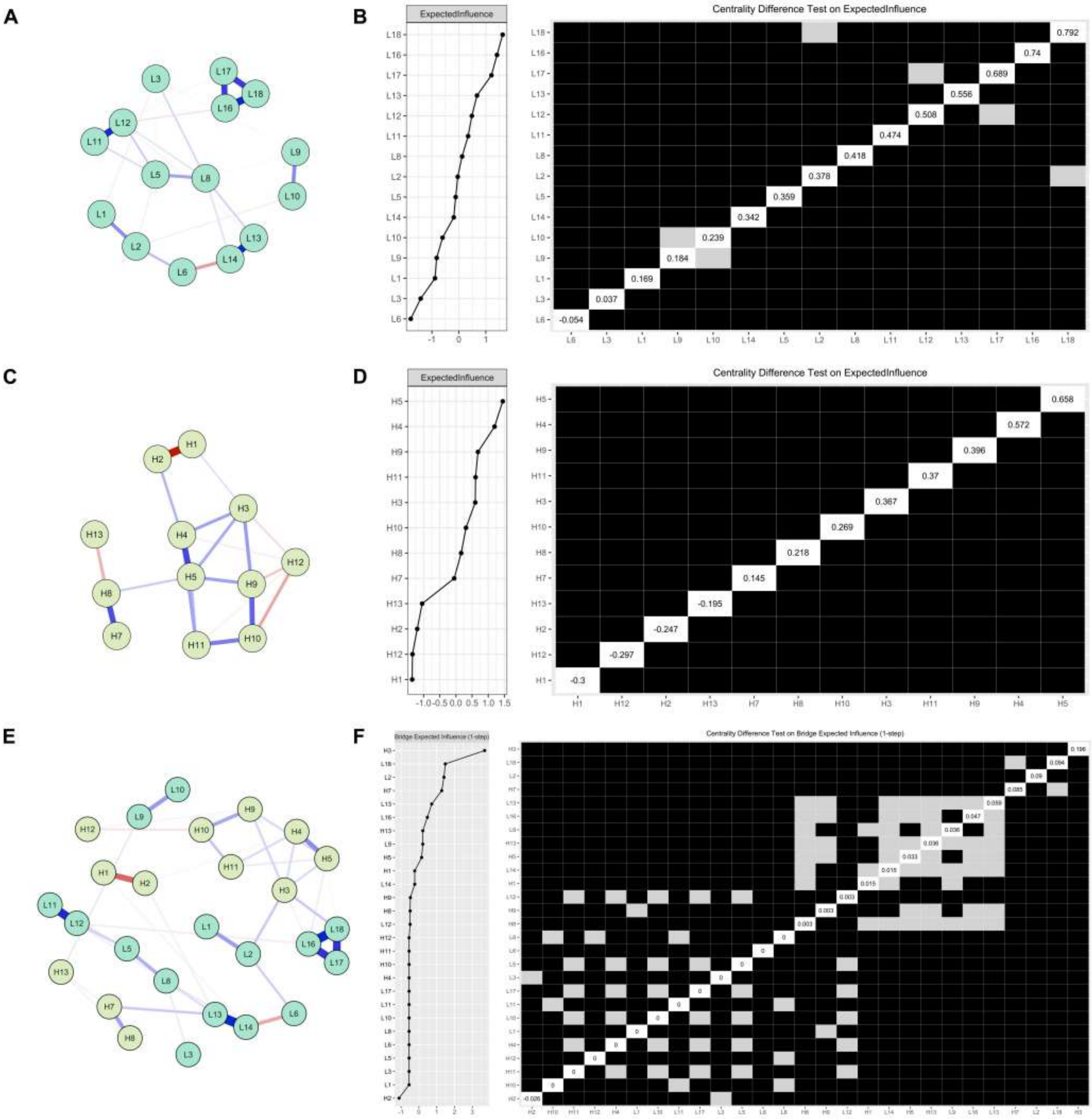

(k)Heart attack

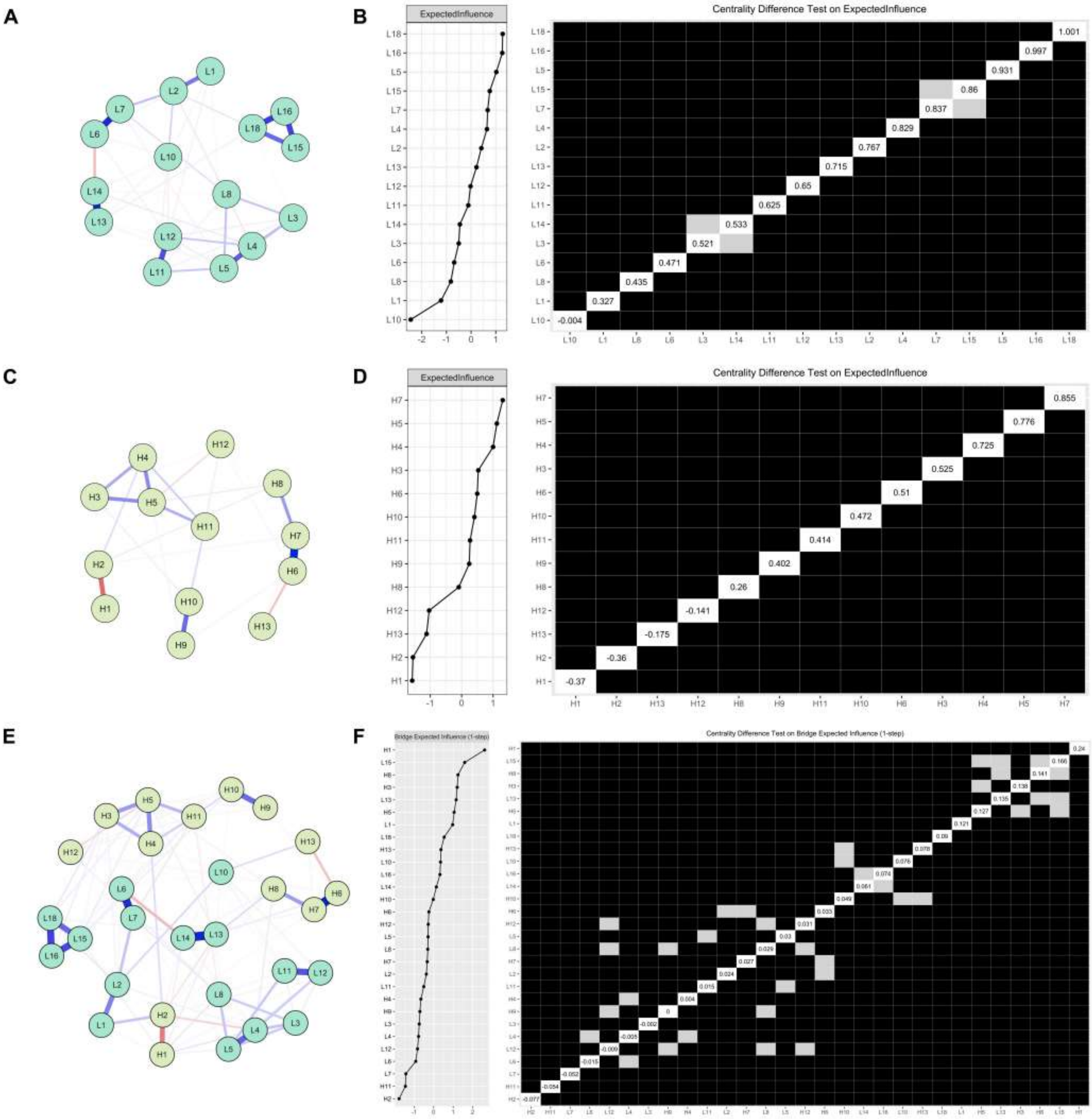

(I) High cholesterol

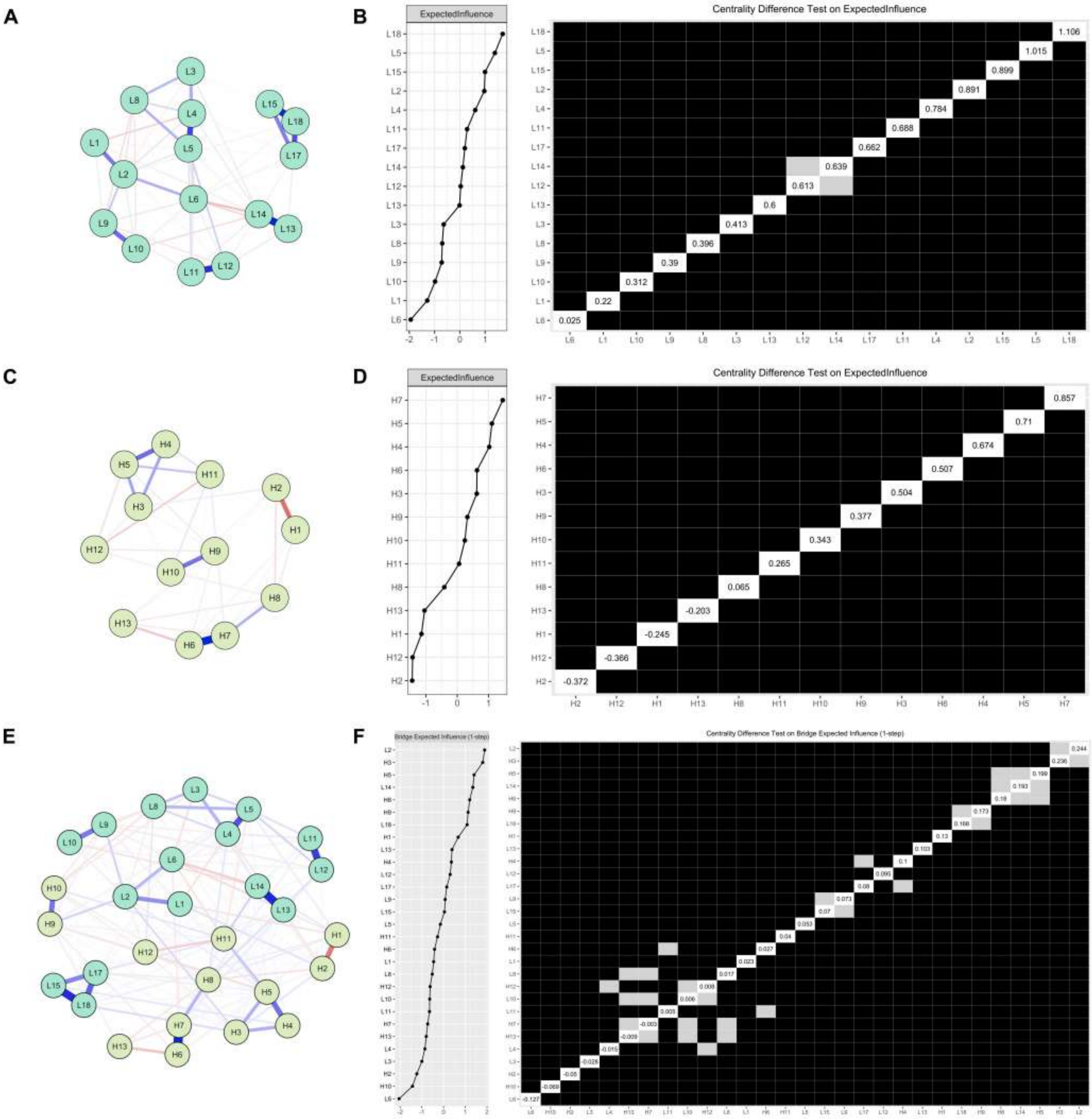

(m) Hypertension

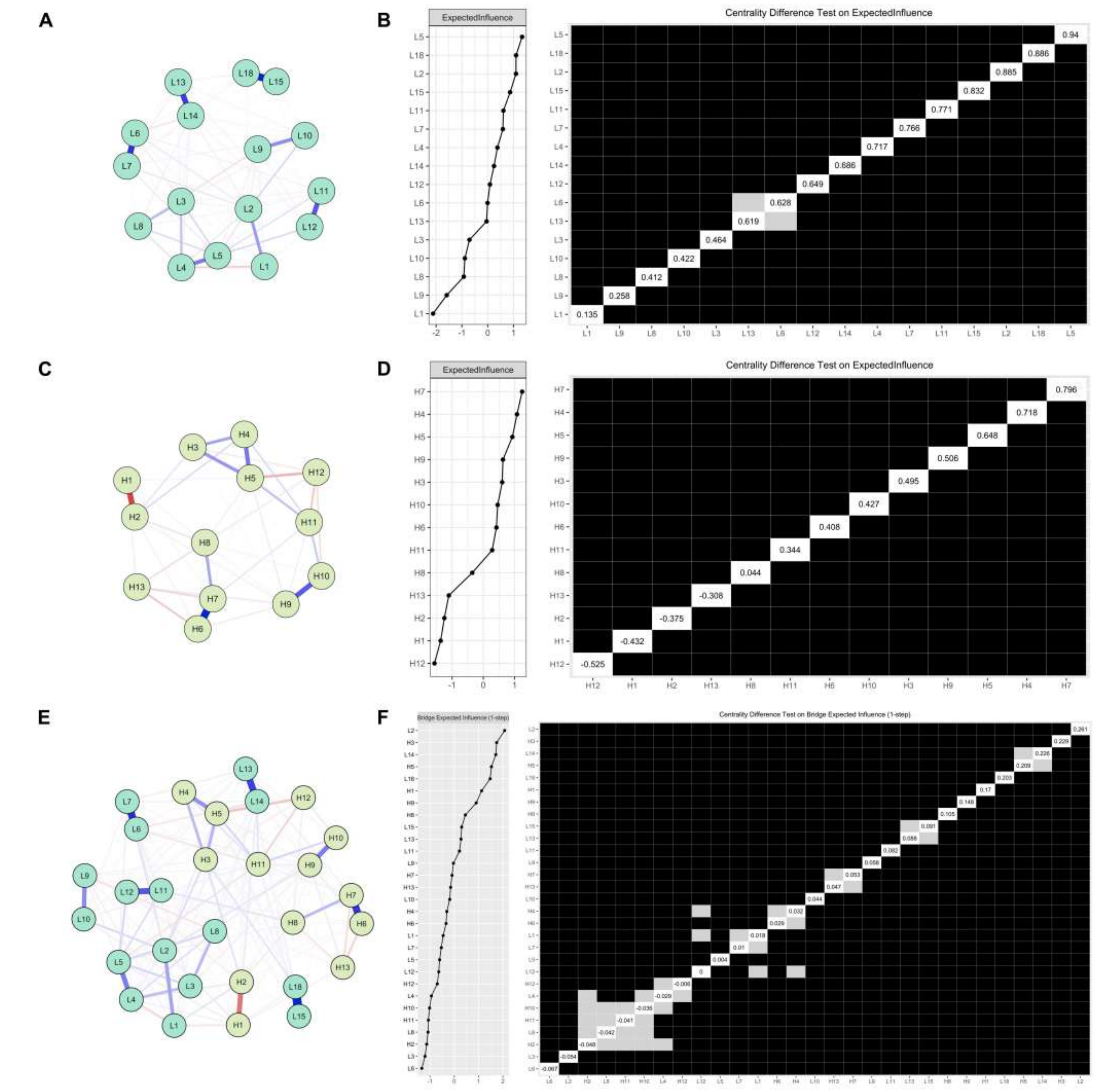

(n) Insomnia

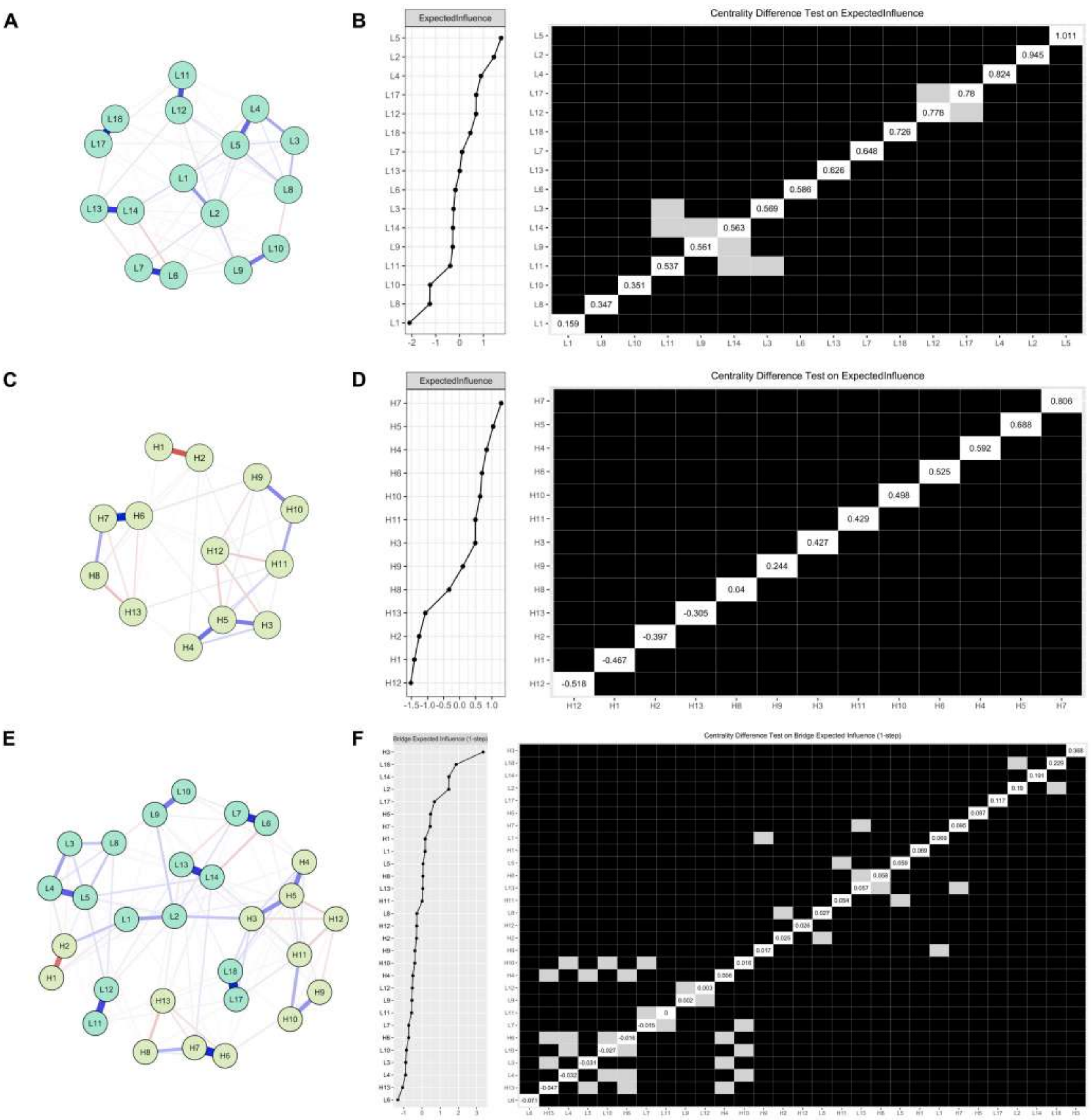
A
B
ExpectedInfluence
Centrality Difference Test on ExpectedInfluence
1.011
0.945
0.824
0.78
0.778
0.726
0.648
0.626
0.586
0.569
0.563
0.561
0.537
0.351
0.347
0.159
C
D
ExpectedInfluence
Centrality Difference Test on ExpectedInfluence
0.806
0.688
0.592
0.525
0.498
0.429
0.427
0.244
0.04
-0.305
-0.397
-0.467
-0.518
E
F
Bridge Expected Influence (1-step)
Centrality Difference Test on Bridge Expected Influence (1-step)

(o) Irritable bowel syndrome

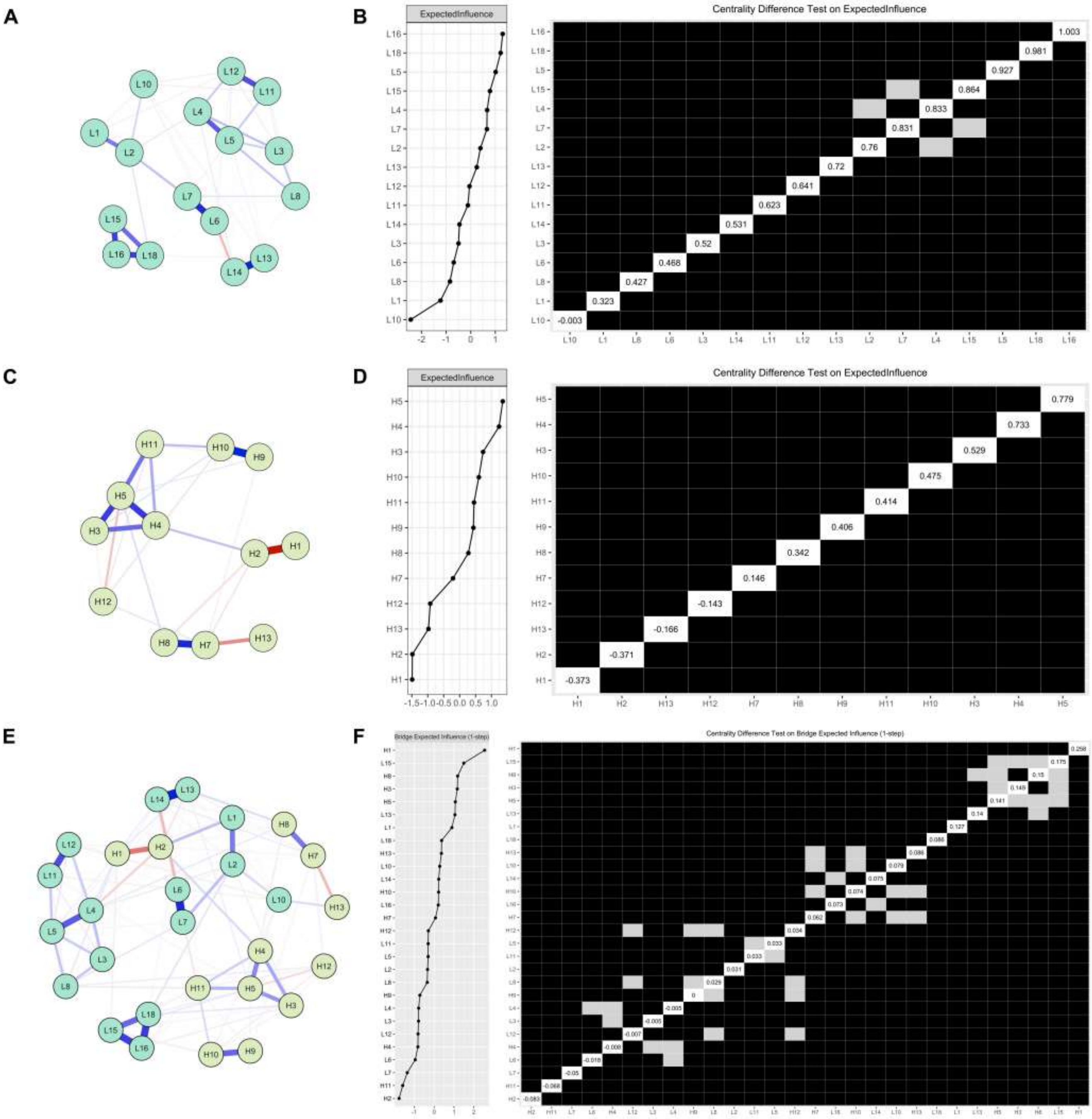

(p) Pre-diabetes

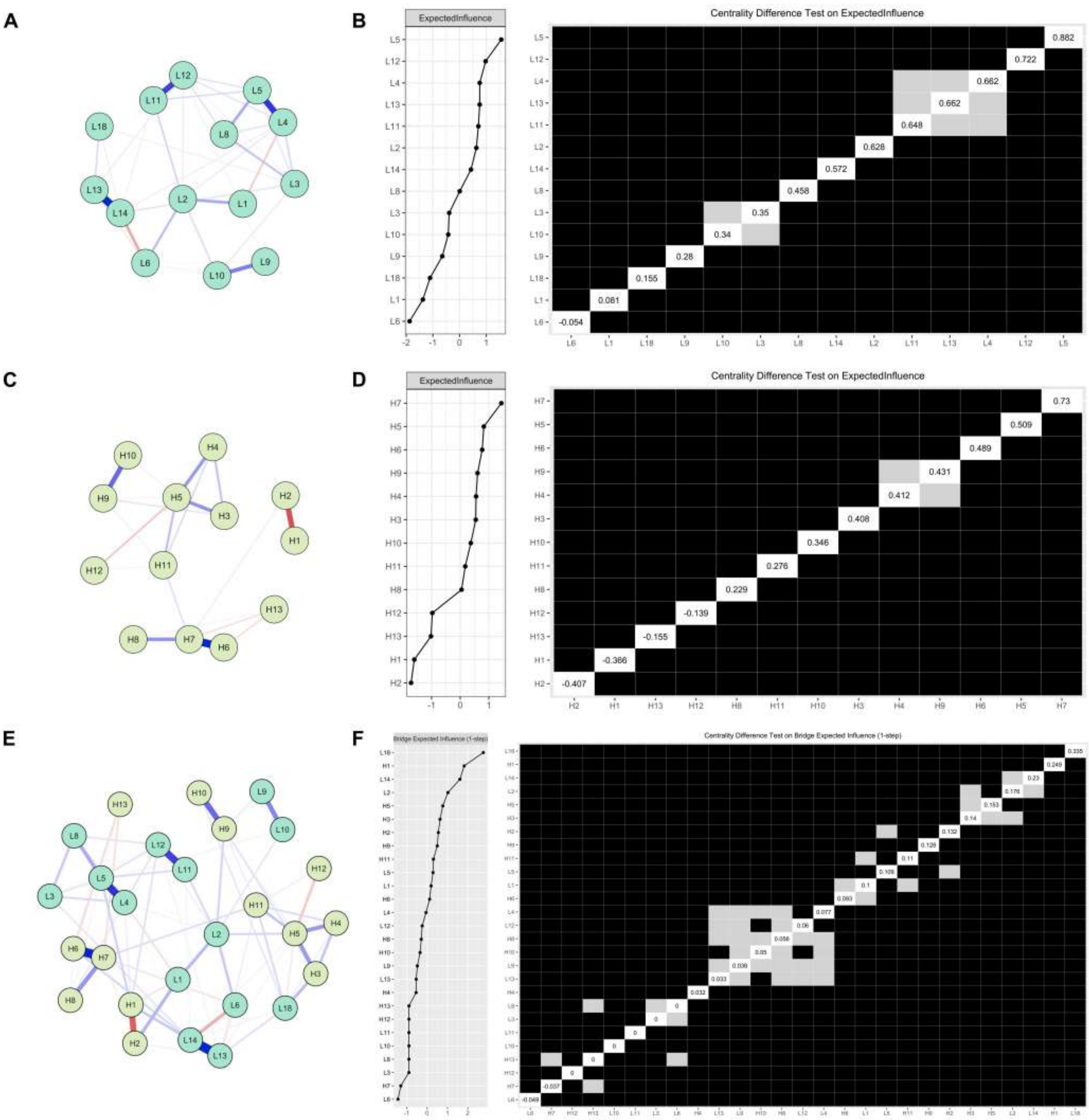

The abbreviation of nodes in Figures (a-p) refers to Supplementary Table 1. For Figures (a-p) B, D, and F, a gray cell indicates no significant difference between the corresponding two variables. A dark cell indicates significant difference between the corresponding two variables at 5% level of significance. A white cell shows the value of expected influence.

Figure S5. Bootstrapped confidence intervals of edge weights for the health outcome network across chronic disease subgroups.

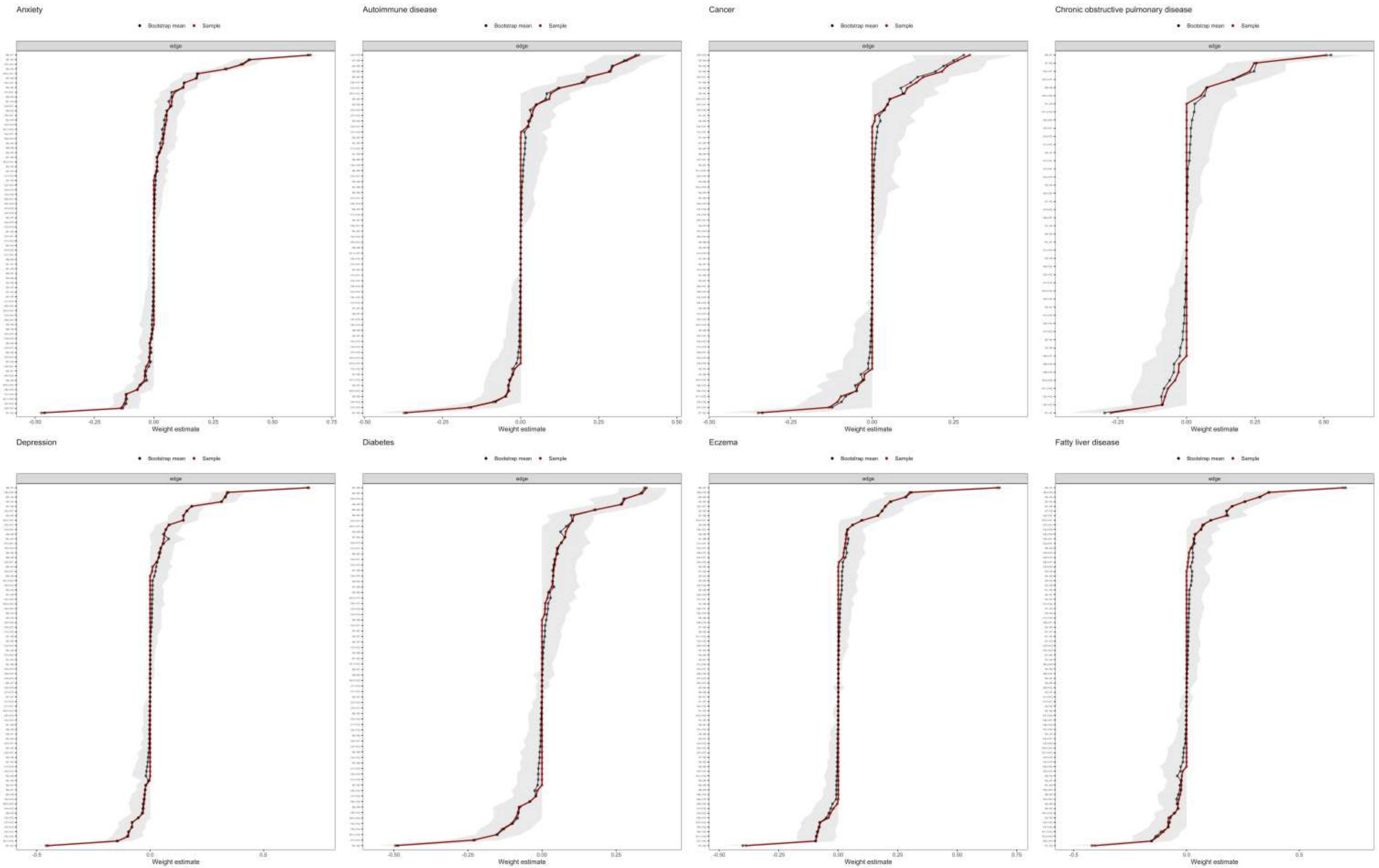

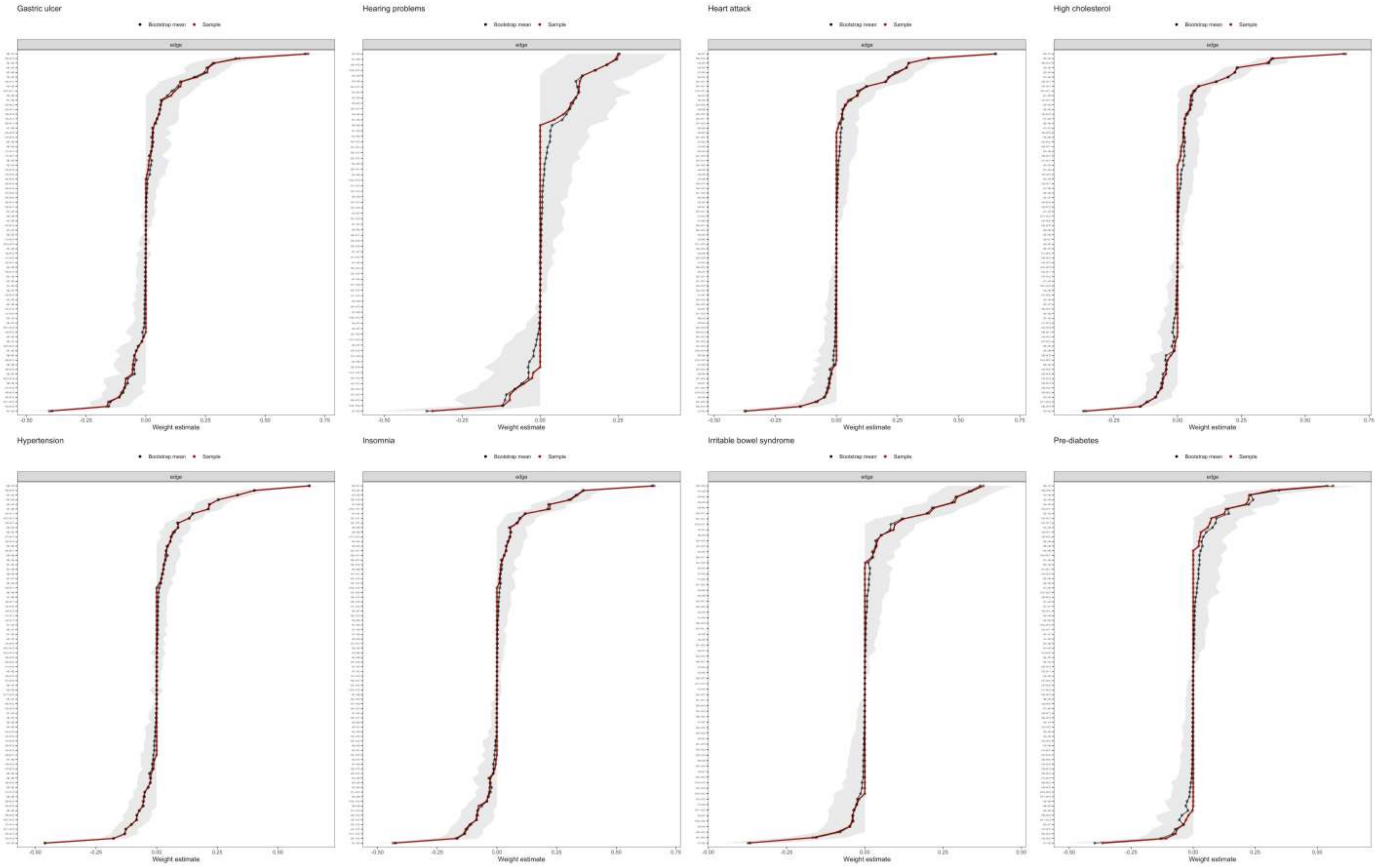

The red dots are sample means per edge, while the black dots are bootstrapped means, ordered from the highest to the lowest value. The gray area represents the 95% confidence intervals of edge weights, estimated with the non-parametric bootstrap procedure (Bootnet package). Wide intervals indicate lower stability and narrow intervals indicate higher stability.

Figure S6. The stability of expected influence centrality index in health outcomes network across chronic disease subgroups using case-dropping bootstrap.

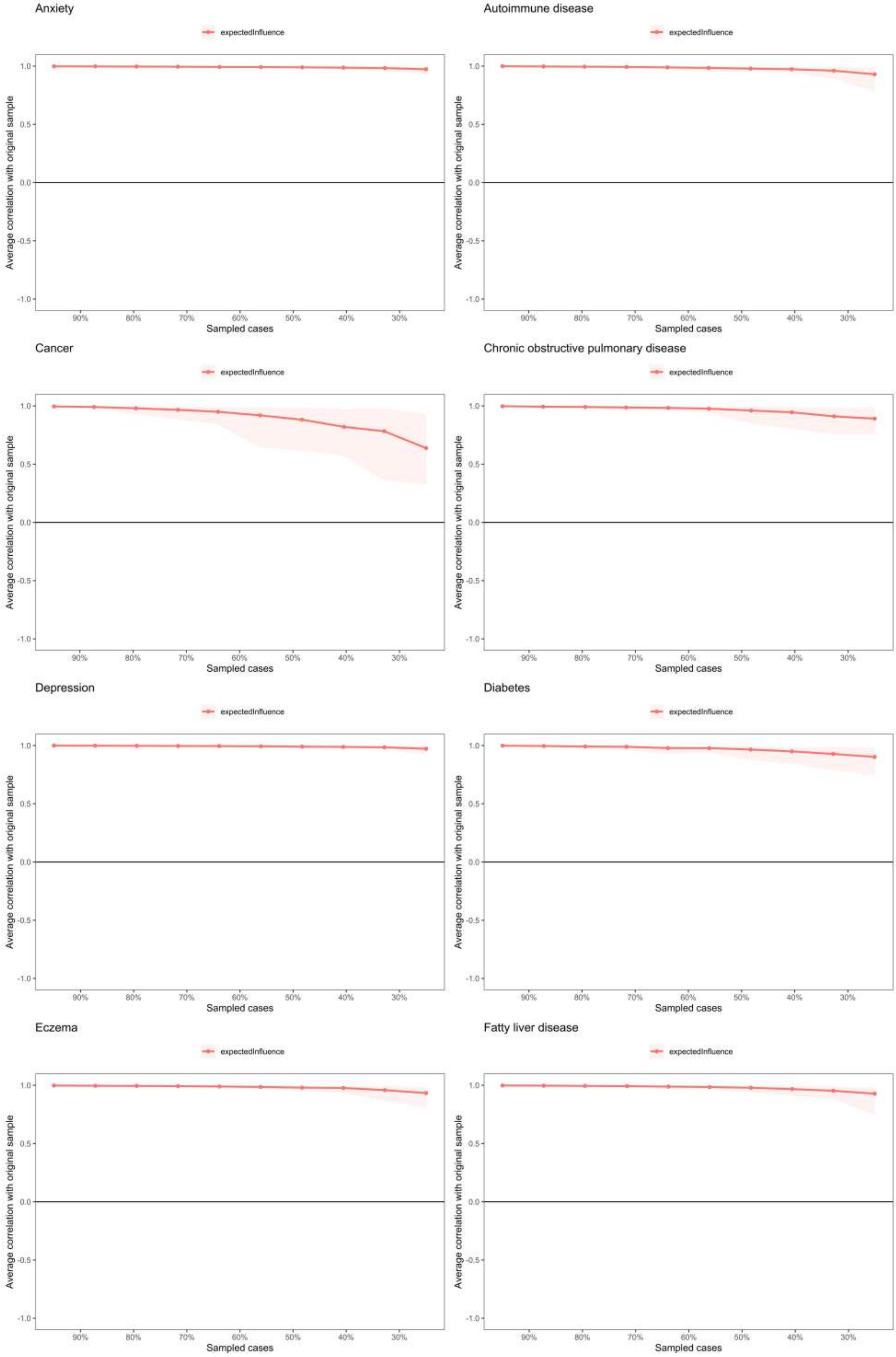

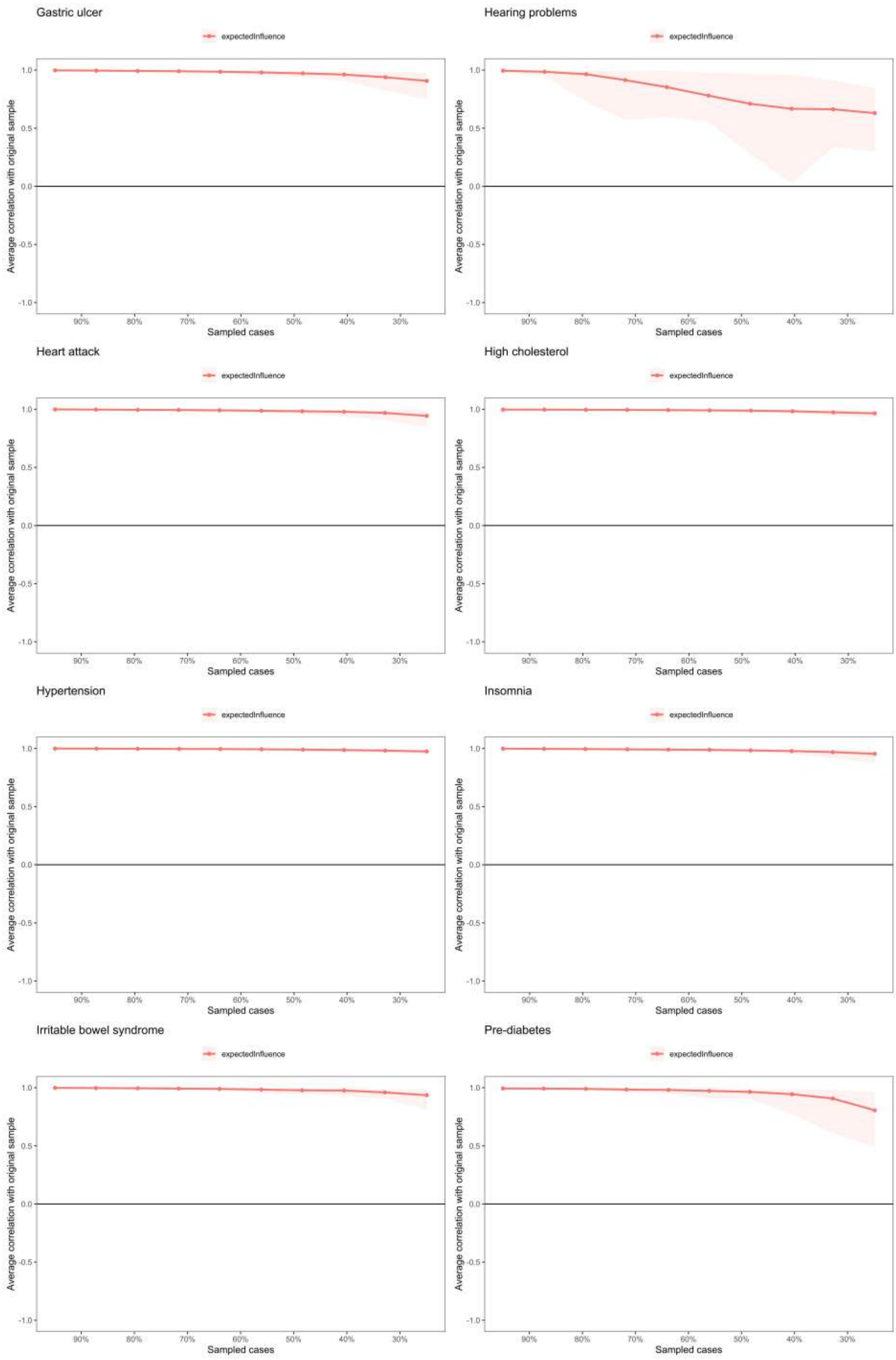

The x-axis indicates the percentage of cases of the original sample included at each step. The y-axis indicates the average of correlations between the expected influence centrality index from the original network and the expected influence centrality index from the networks that were re-estimated after excluding increasing percentages of cases.

Figure S7. Bootstrapped confidence intervals of edge weights for the bridge network across chronic disease subgroups.

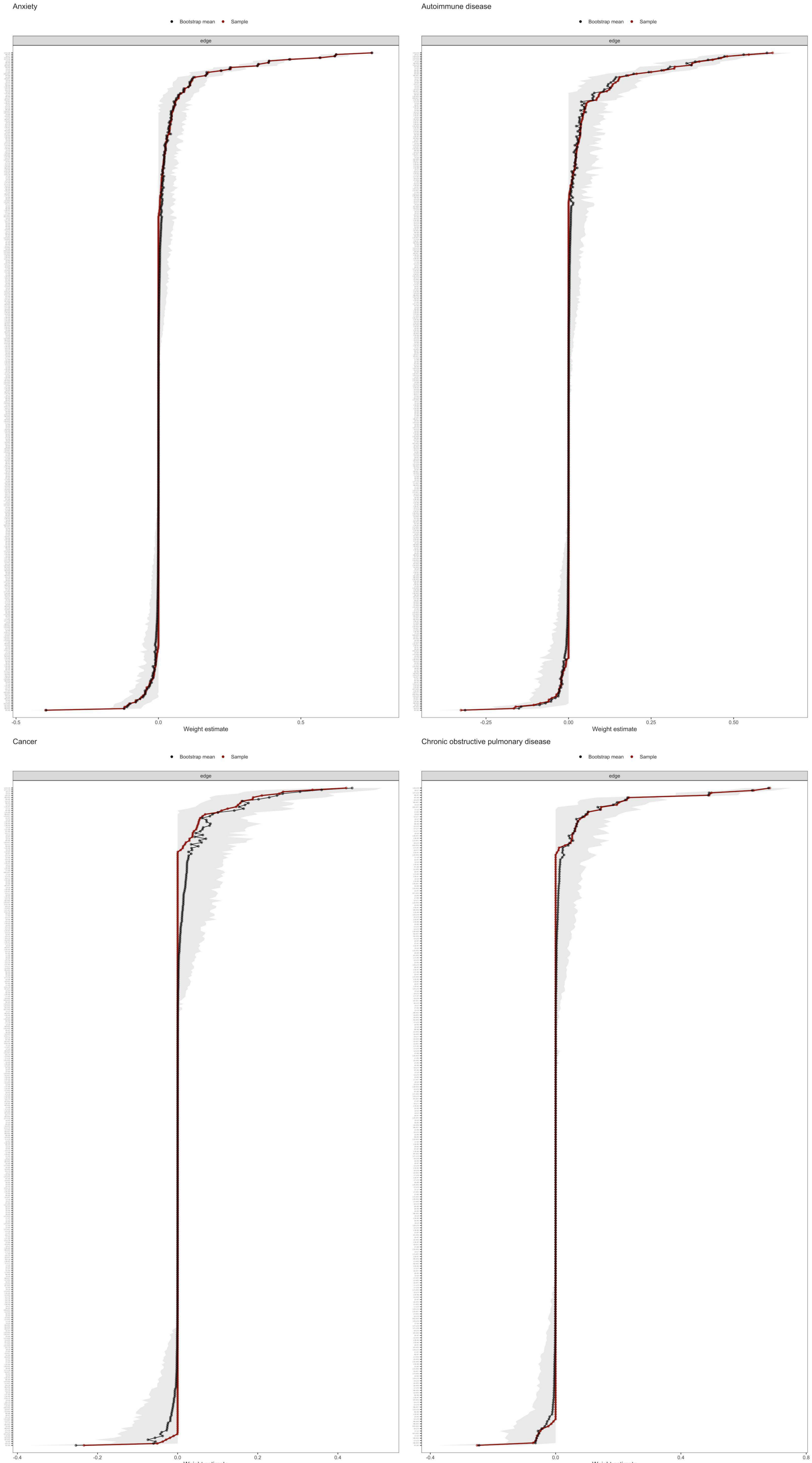

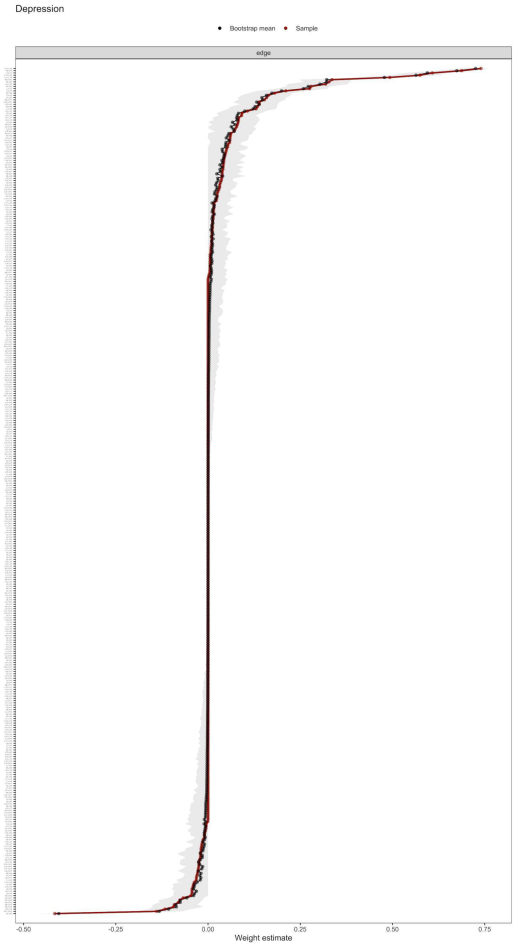
Depression
Bootstrap mean
Sample
edge
-0.50
-0.25
0.00
0.25
0.50
0.75
Weight estimate

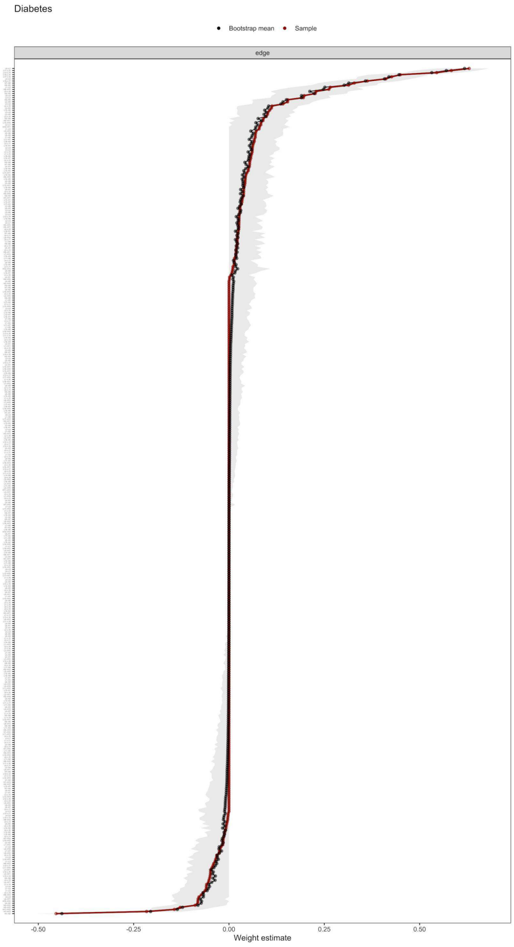
Diabetes
Bootstrap mean
Sample
edge
-0.50
-0.25
0.00
0.25
0.50
Weight estimate

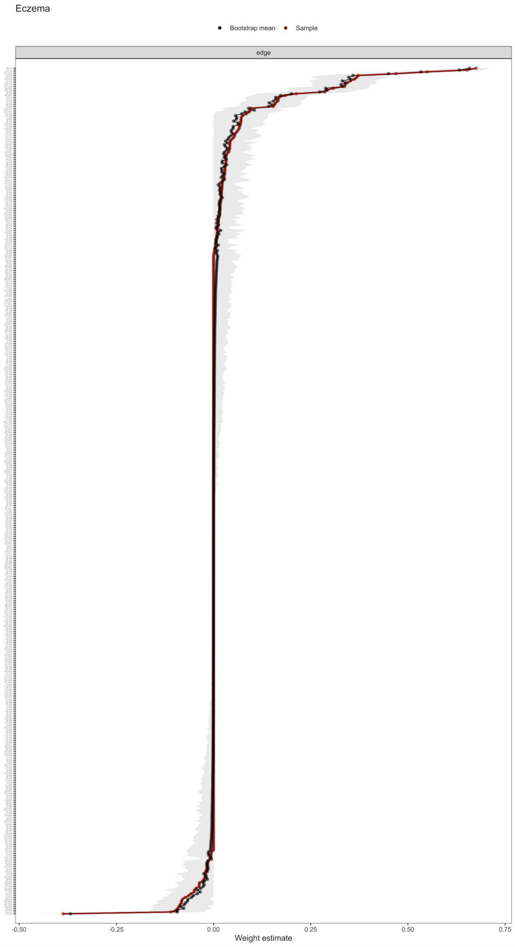
Eczema
Bootstrap mean
Sample
edge
-0.50
-0.25
0.00
0.25
0.50
0.75
Weight estimate

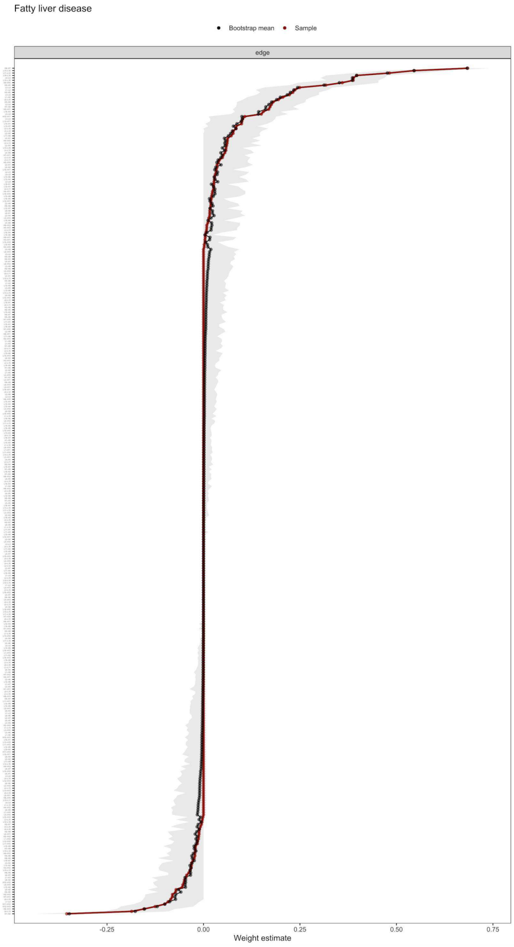
Fatty liver disease
Bootstrap mean
Sample
edge
-0.25
0.00
0.25
0.50
0.75
Weight estimate

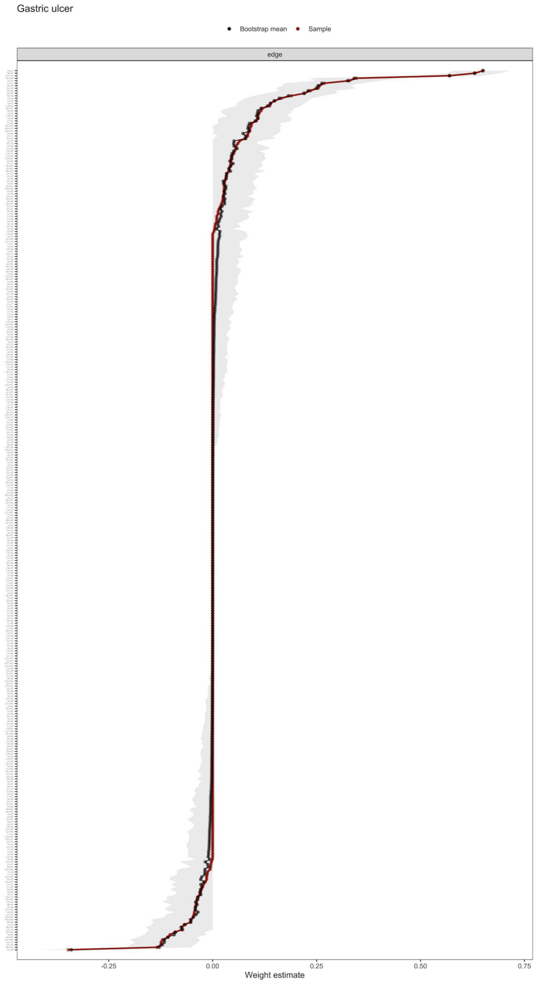
Gastric ulcer
Bootstrap mean
Sample
edge
-0.25
0.00
0.25
0.50
0.75
Weight estimate

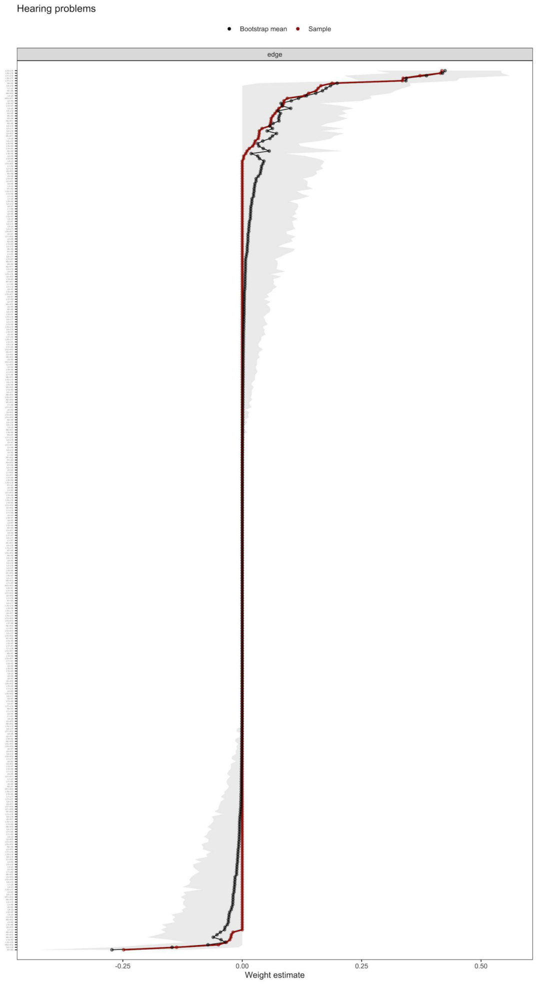
Hearing problems
Bootstrap mean
Sample
edge
-0.25
0.00
0.25
0.50
Weight estimate

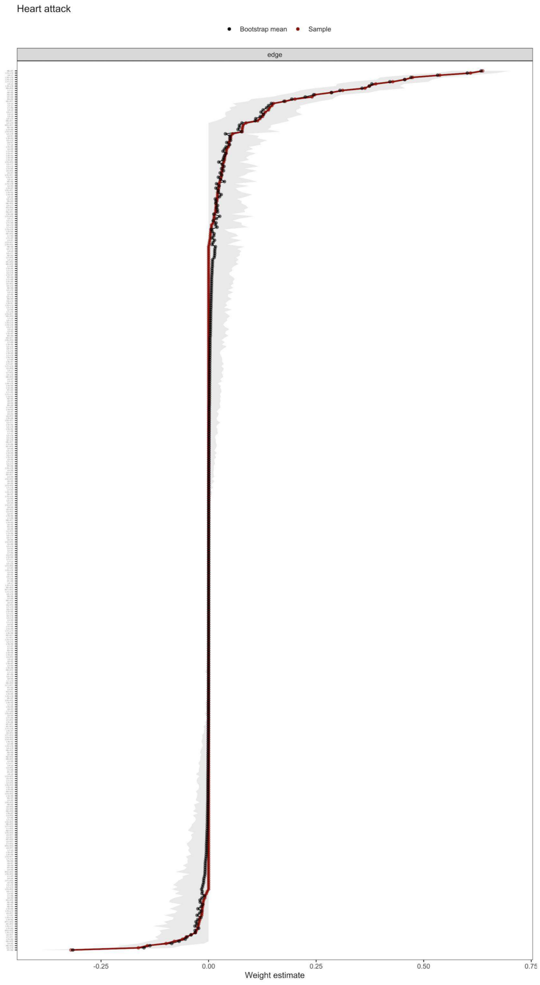
Heart attack
Bootstrap mean
Sample
edge
-0.25
0.00
0.25
0.50
0.75
Weight estimate

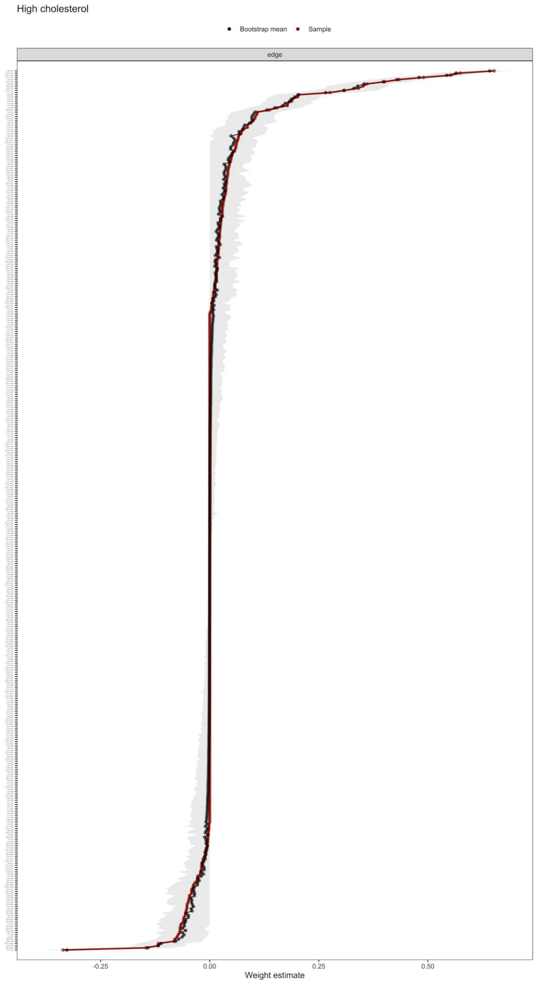
High cholesterol
Bootstrap mean
Sample
edge
-0.25
0.00
0.25
0.50
Weight estimate

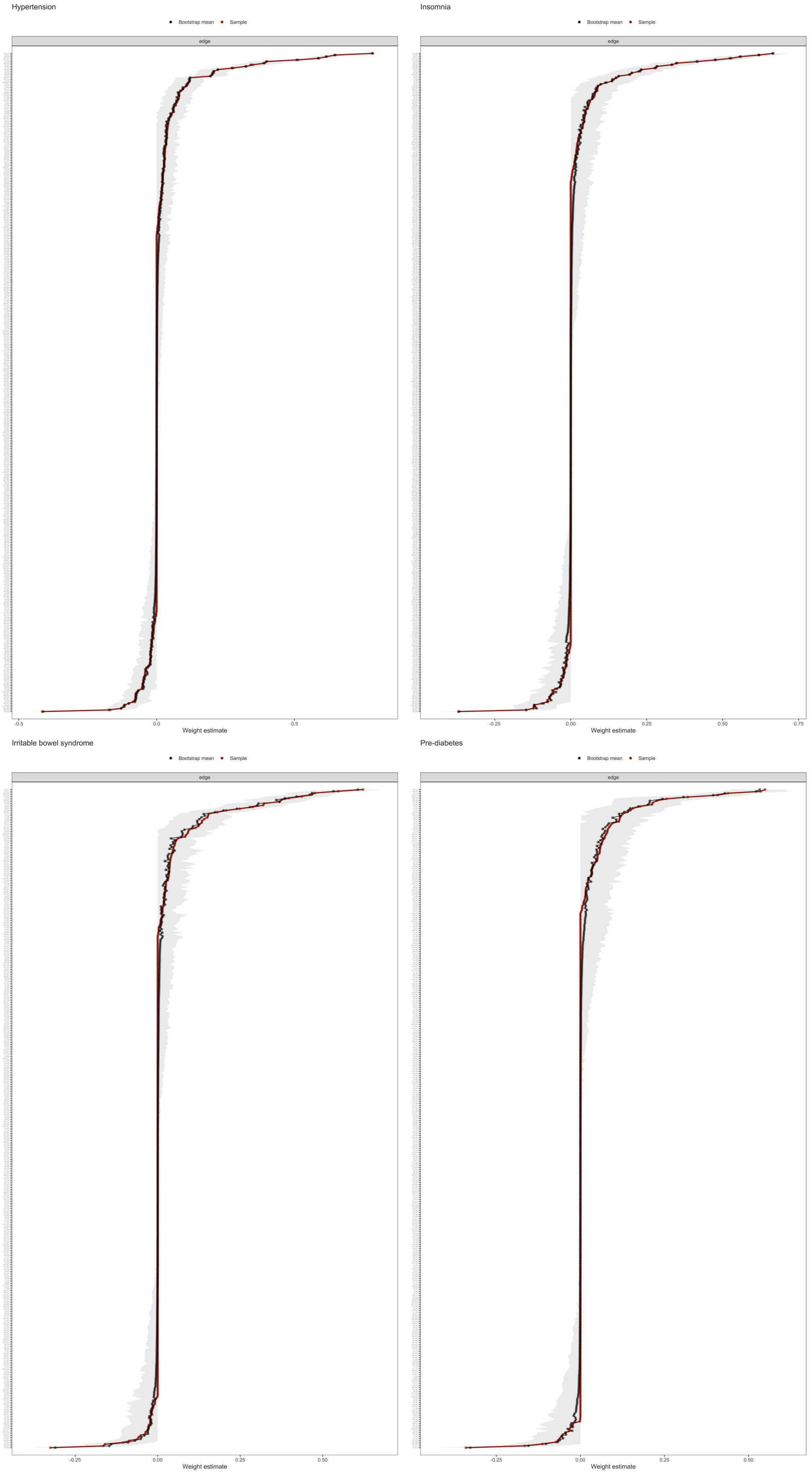

The red dots are sample means per edge, while the black dots are bootstrapped means, ordered from the highest to the lowest value. The gray area represents the 95% confidence intervals of edge weights, estimated with the non-parametric bootstrap procedure (Bootnet package). Wide intervals indicate lower stability and narrow intervals indicate higher stability.

Figure S8. The stability of bridge expected influence centrality index in bridge network across chronic disease subgroups using case-dropping bootstrap.

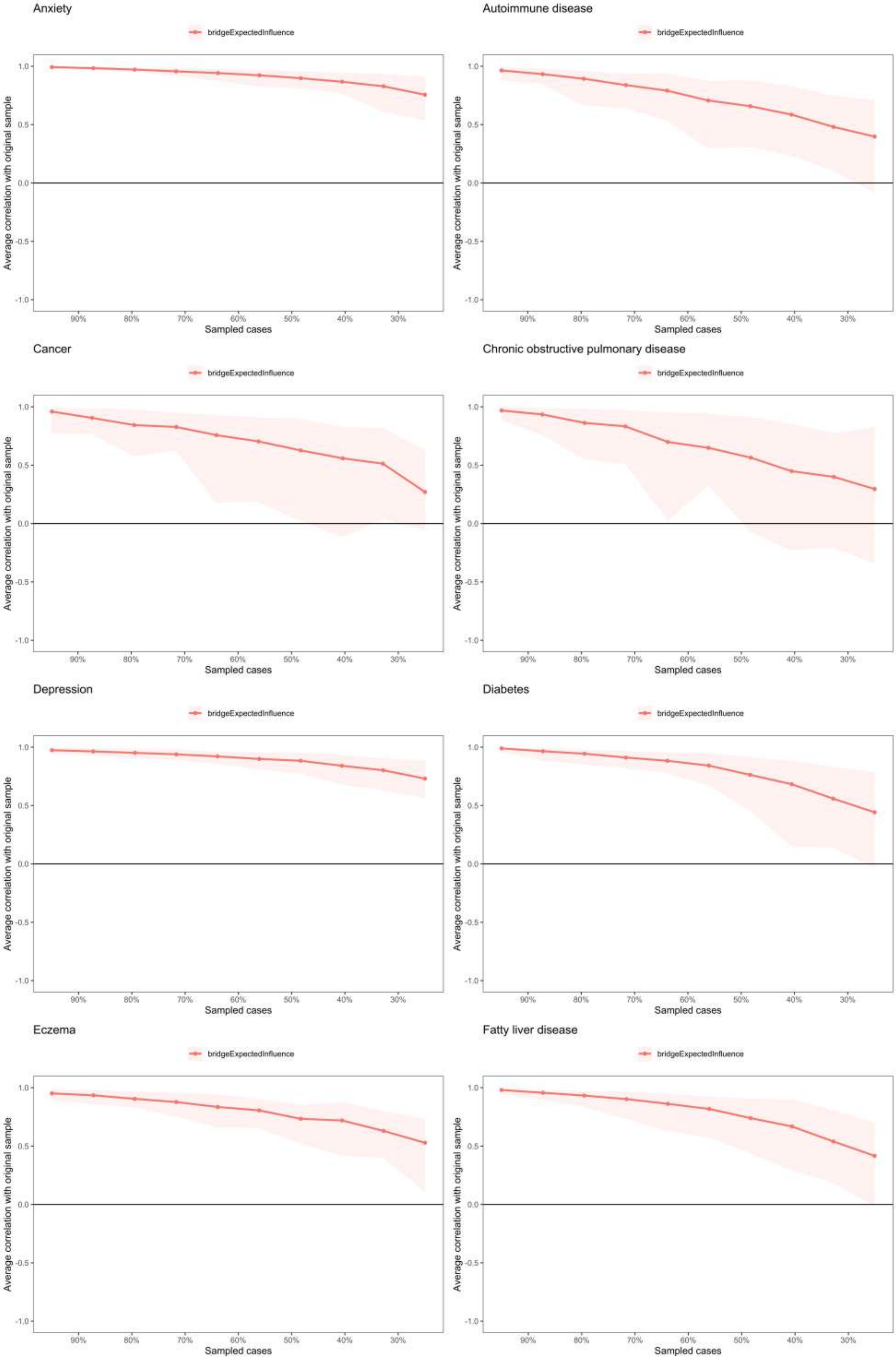

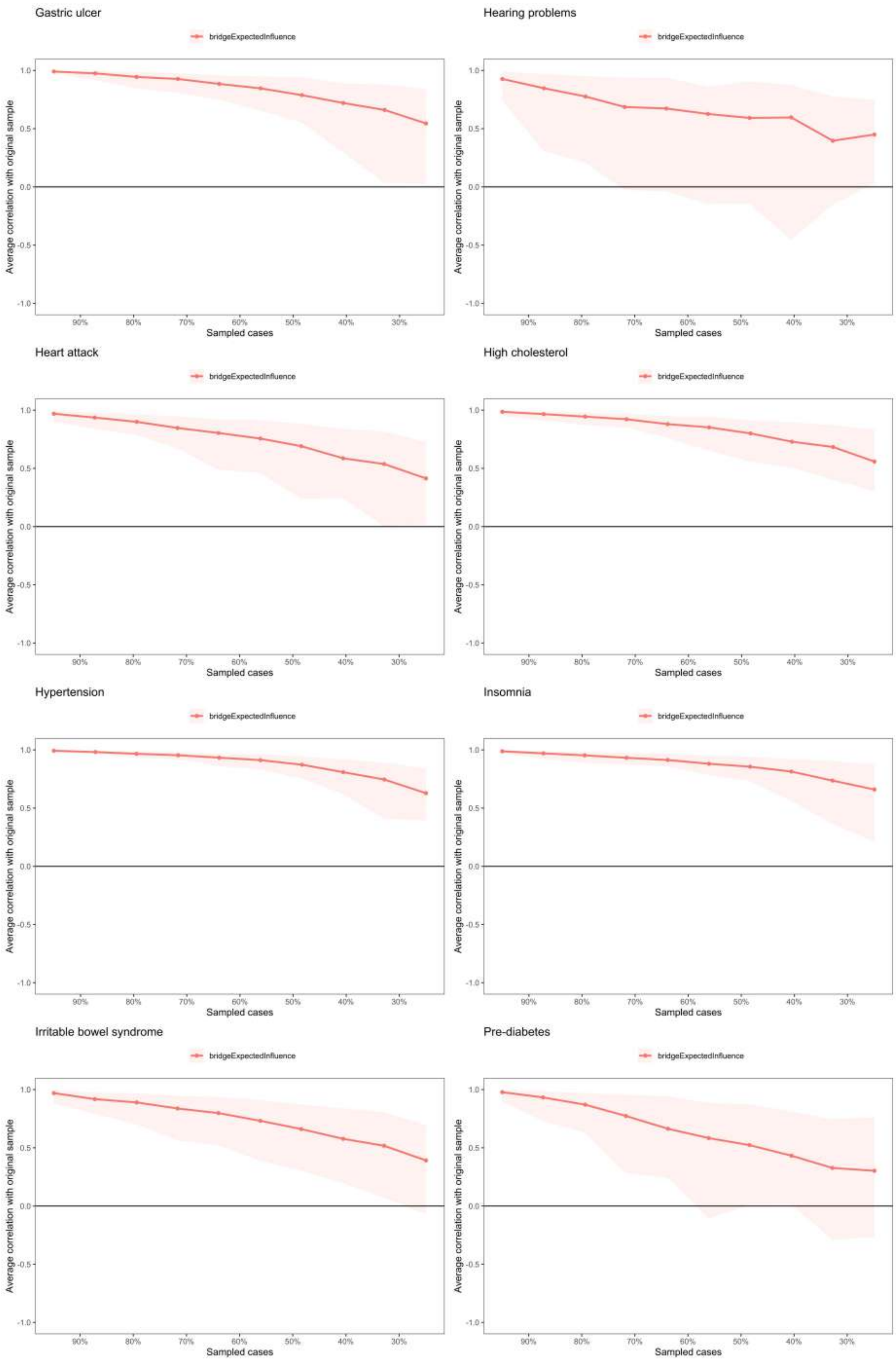

The x-axis indicates the percentage of cases of the original sample included at each step. The y-axis indicates the average of correlations between the bridge expected influence centrality index from the original network and the bridge expected influence centrality index from the networks that were re-estimated after excluding increasing percentages of cases.
